# A novel high-titer, bifunctional lentiviral vector for autologous hematopoietic stem cell gene therapy of sickle cell disease

**DOI:** 10.1016/j.omtm.2024.101254

**Published:** 2024-04-24

**Authors:** Kevyn L. Hart, Boya Liu, Devin Brown, Beatriz Campo-Fernandez, Kevin Tam, Katherine Orr, Roger P. Hollis, Christian Brendel, David A. Williams, Donald B. Kohn

**Affiliations:** 1 Department of Human Genetics, David Geffen School of Medicine, University of California, Los Angeles, Los Angeles, CA 90095, USA; 2 Division of Hematology/Oncology, Boston Children’s Hospital, Boston, MA 02115, USA; 3 Department of Microbiology, Immunology & Molecular Genetics, David Geffen School of Medicine at University of California, Los Angeles, Los Angeles, CA 90095, USA; 4 CSUN-UCLA Stem Cell Scientist Training Program, California State University, Northridge, Northridge, CA 91330, USA; 5 Department of Pediatrics, Harvard Medical School, Boston, MA 02115, USA; 6 Department of Pediatric Oncology, Dana-Farber Cancer Institute, Boston, MA 02215, USA; 7 Harvard Stem Cell Institute, Harvard University, Boston, MA 02138, USA; 8 Department of Molecular and Medical Pharmacology, David Geffen School of Medicine at University of California, Los Angeles, Los Angeles, CA 90095, USA; 9 Department of Pediatrics, David Geffen School of Medicine at University of California, Los Angeles, Los Angeles, CA 90095, USA; 10 The Eli & Edythe Broad Center of Regenerative Medicine & Stem Cell Research, University of California, Los Angeles, Los Angeles, CA 90095, USA

**Keywords:** sickle cell disease, lentiviral vector, shmiR, BCL11A, anti-sickling hemoglobin, gene therapy, hematopoietic stem cells

## Abstract

A major limitation of gene therapy for sickle cell disease (SCD) is the availability and access to a potentially curative one-time treatment, due to high treatment costs. We have developed a high-titer bifunctional lentiviral vector (LVV) in a vector backbone that has reduced size, high vector yields, and efficient gene transfer to human CD34^+^ hematopoietic stem and progenitor cells (HSPCs). This LVV contains locus control region cores expressing an anti-sickling β^AS3^-globin gene and two microRNA-adapted short hairpin RNA simultaneously targeting *BCL11A* and *ZNF410* transcripts to maximally induce fetal hemoglobin (HbF) expression. This LVV induces high levels of anti-sickling hemoglobins (HbA^AS3^ + HbF), while concurrently decreasing sickle hemoglobin (HbS). The decrease in HbS and increased anti-sickling hemoglobin impedes deoxygenated HbS polymerization and red blood cell sickling at low vector copy per cell in transduced SCD patient CD34^+^ cells differentiated into erythrocytes. The dual alterations in red cell hemoglobins ameliorated the SCD phenotype in the SCD Berkeley mouse model *in vivo*. With high titer and enhanced transduction of HSPC at a low multiplicity of infection, this LVV will increase the number of patient doses of vector from production lots to decrease costs and help improve accessibility to gene therapy for SCD.

## Graphical abstract

**Figure:**
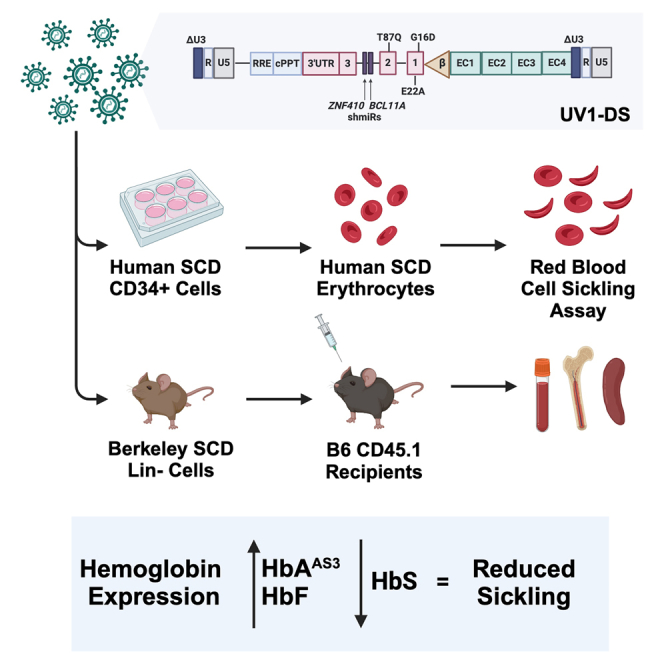

## Teaser

The bifunctional LVV of reduced size led to high vector yields and efficient gene transfer to human CD34^+^ HSPCs. In transduced SCD patient CD34^+^ cells differentiated into erythrocytes, this LVV induces high levels of anti-sickling hemoglobins while decreasing HbS, impeding RBC sickling at a low vector copy per cell.

## Introduction

Sickle cell disease (SCD) is a condition characterized by the production of abnormal hemoglobin (HGB) (sickle HGB [HbS]), caused by a specific genetic mutation in the β-globin gene. This mutation results in the substitution of glutamine for valine at position 6 (E6V) and triggers the polymerization HbS upon deoxygenation. SCD is associated with multiple complications including chronic hemolytic anemia, severe pain episodes (vaso-occlusive events [VOEs]), strokes, and organ damage.^1^ SCD affects approximately 300,000–400,000 newborns annually and around 20 million people worldwide, with an estimated 100,000 individuals affected in the United States.^2^ Current treatments include blood transfusions and medications aimed at reducing VOEs and hemolysis, which include hydroxyurea,^3^ L-glutamine,^4^ crizanlizumab,^5^ and voxelotor,^6^ but these options are non-curative. The only standard of care curative approach is allogeneic hematopoietic stem cell transplantation (HSCT) with a suitably-matched donor. Adverse side effects of allogeneic HSCT include acute and chronic conditioning toxicities, graft failure, and graft-vs-host disease. In addition, many patients lack appropriate donors. In recent years, autologous HSCT/gene therapy for SCD has moved from an attractive concept to clinical reality, with a variety of approaches currently in clinical testing appearing to provide sustained clinical benefits to SCD patients.

The development and manufacturing costs of gene therapy products are significant factors that may pose barriers to the accessibility of curative treatment for patients. The cost of allotransplantation or gene therapy treatment for SCD can vary depending on several factors including the type of therapy used and complications from the treatment, the location of the treatment center, and the cost of the cell product used. With the cost of recently approved gene therapy products being approximately $1–3 million per treatment including the manufacturing of the Medicinal Drug Product as well as the administration of therapy in the setting of a myeloablative transplant with associated medical costs, many individuals will face challenges in accessing approved therapies. To help address these accessibility issues and provide benefit to a larger patient population, it is crucial to develop strategies aimed at decreasing the production costs of complex autologous stem cell gene therapies. Research and development is critical to enhance vector production, increase vector yields, and develop more cost-effective approaches to scale up manufacturing.

One strategy to decrease costs of manufacturing includes engineering smaller lentiviral vectors (LVVs) and enhancing both the efficiency of hematopoietic stem and progenitor cell (HSPC) transduction at lower vector multiplicity of infection (MOI) and enhancing the biological effect of the payload at low MOI in the target cell population. LVVs in current clinical use for the treatment of β-hemoglobinopathies including SCD have relatively large genomes (e.g., 8–9 kb) that include essential enhancer elements from the β-globin locus control region (LCR), to obtain high-level erythroid-specific expression.^7^ These vectors are costly to produce and are relatively inefficient in transducing human CD34^+^ HSPC, in part due to high percentages of incomplete virion genomes, and may require a high MOI due to low expression/integrated vector genome.^8^ They have required the use of transduction enhancer compounds to improve their infectivity at clinical scale. Development of refined β-globin LVV with reduced sizes of the LCR based on bioinformatics design has led to significantly improved titers and CD34^+^ cell infectivity compared with current clinical vectors.^9^

Two successful approaches to genetic therapy for SCD include expressing a modified β-globin gene with anti-sickling characteristics (e.g., T87Q, βAS3), and inducing fetal HGB (HbF) by increasing expression of γ-globin. HbF has potent anti-sickling characteristics and induction via reversing the fetal-adult HGB switch has the additional benefit of concurrently and coordinately decreasing β^S^-globin production. Intracellular polymerization of deoxygenated HGB is exquisitely sensitive to the concentration of HbS in the red cell.^10^ Thus, a strategy to both increase the level of anti-sickling HGB and decrease the concentration of HbS may prove most efficient in decreasing HbS polymerization and thus cellular sickling phenotypes.

We developed an optimized anti-sickling β-globin LVV (UV1)^9^ of minimal size using a bifunctional approach to treating SCD to help address the barriers to accessibility imposed by high vector costs. The first mechanism in this approach incorporates a modified β-globin gene (β^AS3^-globin).^11^ β^AS3^-globin contains three amino acid substitutions (G16D, E22A, and T87Q) that give this β-globin variant anti-sickling properties similar to fetal γ-globin. These amino acid changes incorporated into the β^AS3^-globin polypeptide decrease sickle polymerization through disruption of axial and lateral contact with the canonical valine 6 of sickle β-globin and also confer a competitive advantage over the sickle β-globin chain for binding to α-globin chains to form HGB tetramers. Vectors carrying the β^AS3^-globin transgene corrected hematologic and clinical findings in the Townes Sickle Cell mouse model, and were also shown to transduce SCD patient BM CD34^+^ cells and induce therapeutic levels of HbA^AS3^–globin to correct red blood cell (RBC) physiology.^11^^,^^12^

The second approach incorporated in this vector utilizes microRNA-adapted short hairpin RNAs (shmiRs)^13^ to simultaneously target *BCL11A* and *ZNF410*, two independent repressors of γ-globin expression, to induce HbF. HbF induction is a strategy in current clinical testing to ameliorate SCD phenotypes based on the observation that elevated levels of HbF attenuate clinical severity of SCD.^14^^,^^15^^,^^16^ The prime example is co-inheritance of mutations causing hereditary persistence of HbF (HPFH) with SCD leads to marked attenuation of SCD phenotypes in comparison to individuals without HPFH.^17^ BCL11A was identified as an important repressor of fetal globin expression based on genome-wide association study mapping.^18^^,^^19^ Further studies showed that generating a *BCL11A* knockout in SCD mouse models corrected the pathogenic defects associated with SCD through increased HbF expression.^20^ An LVV expressing a *BCL11A* shmiR (BCH-BB694) only in the erythroid lineage under the control of the β-globin promoter and regulatory elements derived from HS2 and HS3 of the LCR ameliorated the sickle phenotype in mice and induced up to 40% HbF induction in erythroid differentiated SCD CD34^+^ cells.^21^ A Phase I clinical trial with BCH-BB694 showed a sustained increase of HbF levels with a median of 30.5% of all HGB levels in six patients with significant mitigation of sickle phenotype at an average *in vivo* vector copy number (VCN) of approximately 1 copy per diploid genome.^22^ In addition to BCL11A, ZNF410 has also been shown to be a repressor of γ-globin expression.^23^^,^^24^ Combining *BCL11A* and *ZNF410* shmiRs has been shown to increase HbF induction by approximately an additional 10% compared with knockdown of *BCL11A* alone, with enhanced anti-sickling results in SCD erythroid differentiated CD34^+^ cells.^13^ Brusson et al.^25^ reported a bifunctional LVV for SCD that expressed the same β^AS3^-globin gene described here combined with an artificial miR to HbS. They observed a higher level of correction of parameters of SCD by this bifunctional vector compared with one expressing only the β^AS3^-globin gene.

Here we have combined both approaches in a small, highly efficient vector for treating SCD. The bifunctional vector described here (UV1-DS) maintains high titers of production and high CD34^+^ cell transduction activity at reduced MOI, which may provide a lower cost of manufacturing for the LVV component of autologous hematopoietic stem cell gene therapy. We demonstrate that differentiation of hematopoietic stem/progenitor cells (HSPCs) transduced with the UV1-DS vector leads to reversal of the sickle cellular phenotype with cellular parameters equivalent to normal red blood cells.

## Results

### Design and assessment of the UV1-DS vector

**Figure 1:**
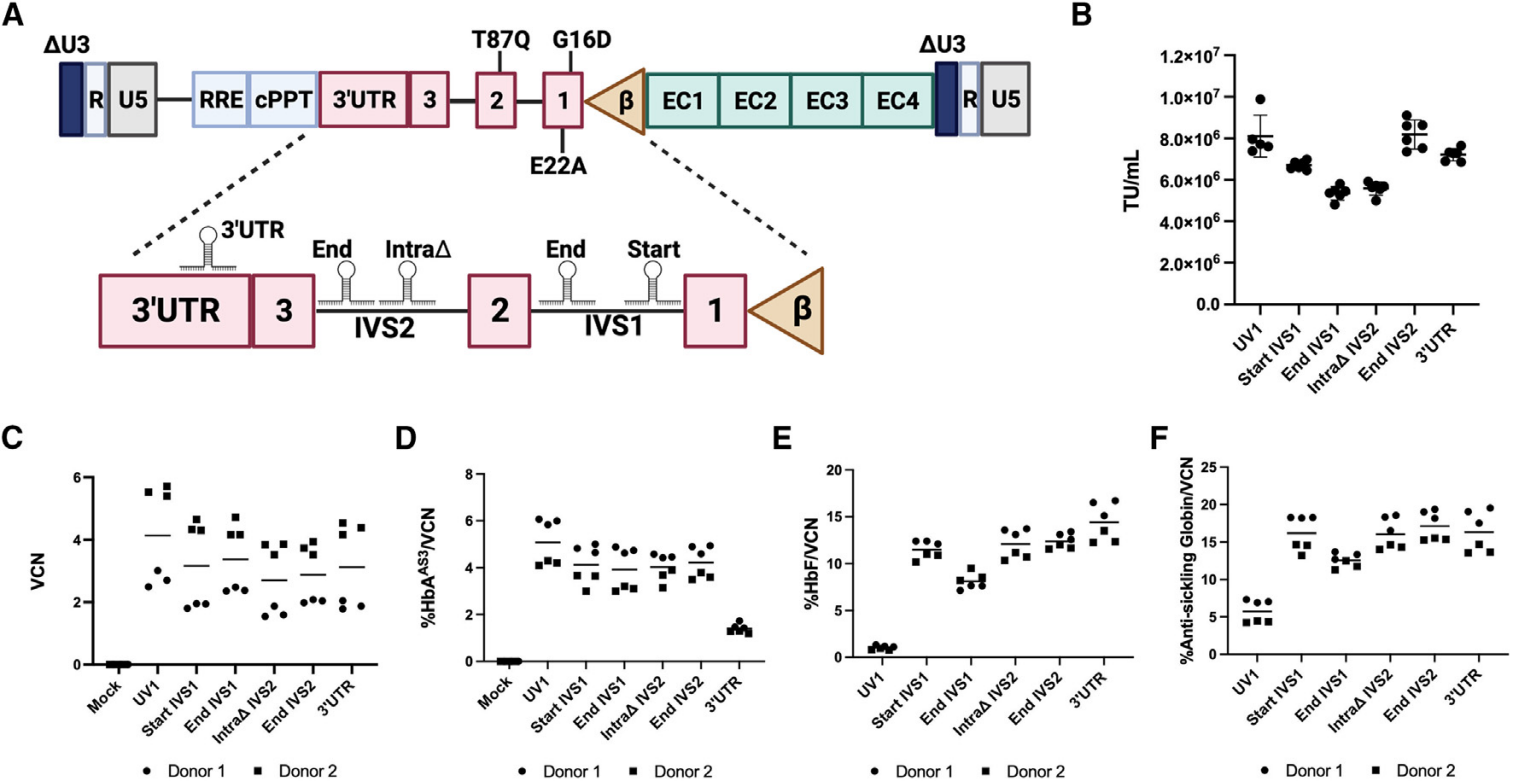
Assessment of the optimal location to incorporate the *BCL11A* shmiR into the UV1 vector (A) Schematic of UV1-shmiR LVV series and cloning design. The UV1 LVV containing a β^AS3^-globin cassette in reverse orientation expressed under the control of the β-globin promoter and Encode core (EC) enhancer regions derived and minimized from hypersensitive sites 1, 2, 3, and 4 (HS1, HS2, HS3, and HS4) of the β-globin LCR.^9^ Insertion DNA sequence sites in Table S1. (B) Unconcentrated viral titers of vectors in which the *BCL11A* shmiR^27^ was introduced into 5 locations including: two sites in intron 1 (Start, End), two sites in IVS2 (IntraΔ, End), and one site in the 3′UTR. Then X axis denotes different vectors and the Y axis shows titer as determined in materials and methods. (C–F) CD34^+^ HSPCs from two healthy donors were transduced in triplicate with vector constructs at 2 × 10^7^ TU/mL and differentiated *in vitro* under erythroid conditions. Cells were collected at day 14 for (C) VCN analysis measured by ddPCR, and day 18 for (D) HbA^AS3^ and (E) HbF by HPLC, (F) total anti-sickling globin (%HbA^AS3^-globin + %HbF) quantification measured by HPLC. Error bars represents means ± SD. (A) created with BioRender.

Previous studies have shown success in ameliorating the sickle cell phenotype with both *BCL11A* shmiR and β^AS3^-globin technologies. To determine whether combining these technologies could be accomplished with effective packaging and higher titers and improved gene transfer we cloned the *BCL11A* shmiR into the UV1 vector that expresses β^AS3^-globin. To define the optimal location to incorporate the *BCL11A* shmiR into the UV1 vector, we cloned the *BCL11A* shmiR sequences into five different locations throughout the β^AS3^-globin cassette. Multiple locations were selected due to the possibility of the shmiR disrupting β^AS3^-globin RNA processing. The locations consisted of two sites in intron 1 (at the start of IVS1 and the end IVS1), two locations in intron 2 (intraΔ and the end IVS2), and one location in the 3′UTR (Figure 1A). Intron 2 had been previously modified by removing sequence that were detrimental to high titer vector production.^26^ Removal of this region also decreased the length of the vector without significantly decreasing β^AS3^-globin expression. These locations were further screened bioinformatically to avoid mRNA splicing and branchpoint sequences.

The resulting vector plasmids were Sanger sequenced to confirm correct construction and packaged using a HEK293T *PKR* knockout cell line. A host *PKR* response is initiated when transfecting with opposite oriented expression cassettes leading to inhibited synthesis of viral proteins resulting in lower titers.^28^ This producer line is designed to yield higher titers from the vectors with reverse orientation expression cassettes and has been shown to increase titers of β^AS3^-globin vectors by 2- to 5-fold.^28^^,^^29^ We saw a minimal decrease in the unconcentrated viral titers with any of the five UV1-shmiR vectors in comparison to the UV1 control (Figure 1B). In particular, the shmiR in the end IVS2 position retained a comparable titer to the parental UV1 vector.

To assess the functionality of the vectors, we transduced CD34^+^ cells at an MOI of 20 from two different healthy donors, each in triplicate, and performed erythroid differentiation. There was a mean VCN of 4.1 ± 2.0 for UV1 and a range of mean VCN from 2.7 ± 1.5 to 3.4 ± 1.4 for all of the UV1-shmiR vectors (Figure 1C).

High-performance liquid chromatography (HPLC) analysis of HGB species in the differentiated erythrocytes demonstrated that each of the UV1-shmiR vectors induced similar mean expression of β^AS3^-globin per copy (3.9 ± 1.2% to 4.2 ± 0.8%) (Figure 1D), except for the vector with the *BCL11A* shmiR in the 3′UTR location. The UV1-shmiR vectors expressed 8.1 ± 0.9% to 14.4 ± 2.4% fetal globin per vector copy (Figure 1E). Percentages of total anti-sickling hemoglobins were calculated as Hbβ^AS3^ expression plus HbF expression. All UV1-shmiR combination vectors outperformed UV1 in the total anti-sickling hemoglobins produced.

From these data, we concluded that four out of the five locations performed well for inducing anti-sickling β^AS3^-globin expression. The location at the End-IVS2 position (**UV1-SS**) was selected for further studies as it was the candidate vector that retained a high titer and high anti-sickling HGB expression. At a VCN of 1, UV1-SS led to a mean expression of 17.1% ± 2.4% anti-sickling hemoglobins (HbA^AS3^ plus HbF) compared with 5.7% ± 1.9% for UV1 (Figure 1F).^16^

### UV1-DS, a double shmiR (DS) vector in the UV1 backbone incorporating the *ZNF410* shmiR with the *BCL11A* shmiR and β^AS3^-globin

**Figure 2:**
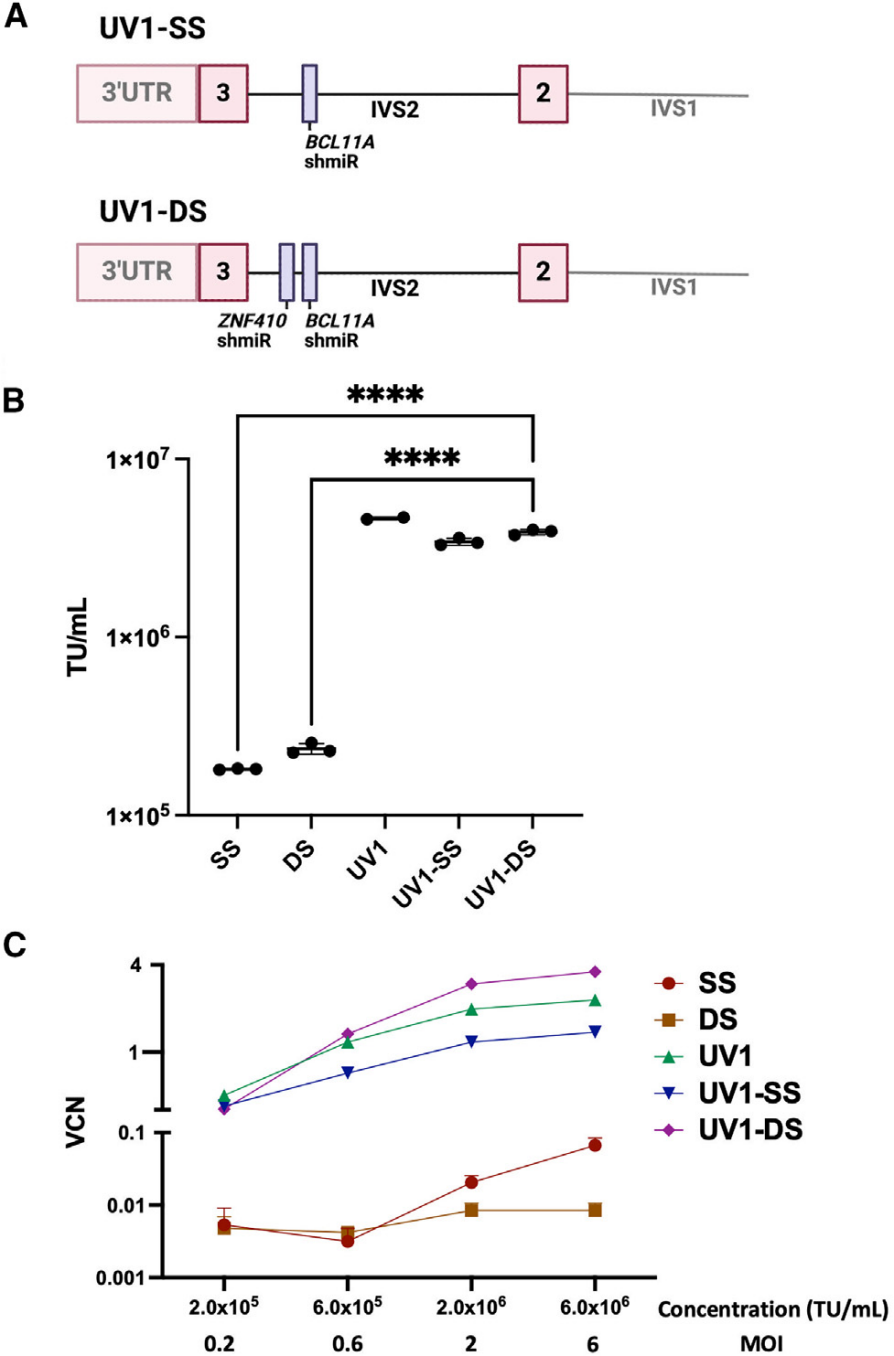
Maps, titers, and gene transfer efficiency of LVVs with SS or DS (A) Map of **UV1-SS** (single shmiR) LVV containing the *BCL11A* shmiR at the end of IVS2 in the β^AS3^-globin cassette. Map of **UV1-DS** (double shmiR) LVV containing both the *BCL11A* and *ZNF410* shmiR at the end of IVS2 in the β^AS3^-globin cassette. Maps show detail of intron 1, site of shmiR insertion, and surrounding β-globin exons 2 and 3. (B) Vectors were packaged as described in Materials and methods and titers were determined on HT-29 cells and quantified with ddPCR. Unconcentrated viral titer shown here. **SS** and **DS** represent vectors with SS or DS, expressed in a β-globin LCR-driven LVV previously described.^13^^,^^30^ Each point on the plot represents vector packaged and titered from an individual 10-cm plate. n = 3. (C) CD34^+^ cells from a healthy donor were transduced with constructs at 2 × 10^5^ TU/mL, 6 × 10^5^ TU/mL, 2 × 10^6^ TU/mL, and 6 × 10^6^ TU/mL (MOIs of 0.2, 0.6, 2, and 6) and cultured for 14 days in myeloid differentiation conditions to assess levels of infectivity. VCN was measured by ddPCR. n = 2. Error bars represent means ± SD; Tukey’s multiple comparisons test was performed on all arms with selected statistics shown. ∗∗∗∗*p* < 0.0001. (A) created with BioRender.

Incorporating the *ZNF410* shmiR with the *BCL11A* shmiR has been shown to increase fetal globin induction by approximately an additional 10%.^13^ A vector was cloned to incorporate both the *BCL11A* and *ZNF410* shmiR at the END IVS2 location, creating a DS vector in the UV1 backbone (**UV1-DS**) (Figure 2A). The performance of five candidate vectors were then compared, including UV1-DS, UV1-SS, UV1, DS^13^ (contains *ZNF410* and *BCL11A* shmiR), and single shmiR (SS)^30^ (contains *BCL11A* shmiR). The shmiR sequences targeting *BCL11A* and *ZNF410* in the UV1 vectors are the same shmiR sequences in DS and SS. DS and SS will induce HbF expression, but are not in the UV1 backbone and, therefore, do not encode the β-globin gene. Vectors were packaged in a HEK293T *PKR* knockout cell line^8^ and unconcentrated and concentrated viral titers were determined through transduction of the HT-29 cell line. Unconcentrated titers across all vectors in the UV1 backbone were comparable with mean titers between 3.4 ± 0.2e+06 TU/mL and 4.6 ± 0.1e+06 TU/mL (Figure 2B). The vectors containing shmiRs, but not in the UV1 backbone, had titers approximately 20-fold lower, with mean titers between 1.8 ± 0.0e+05 and 2.4 ± 0.0e+05 TU/mL (Figure 2B).

Gene transfer efficiency into healthy donor CD34^+^ cells was analyzed using all five vectors. CD34^+^ cells were transduced with concentrated viral supernatant at four different MOIs. Transduced CD34^+^ cells were differentiated using myeloid cytokine stimulation (IL-3, IL-6, and ckit ligand) for 14 days and VCNs were measured using a droplet digital PCR (ddPCR) assay. Myeloid differentiation was used instead of erythroid differentiation, as a myeloid cell VCN has been demonstrated to more closely predict the VCN seen *in vivo* in bone marrow after xenotransplantation of immune-deficient mice.^12^ Notably, the VCN of DS and SS plateaued, with increasing vector concentration not resulting in increased gene transfer. Vectors with the UV1 backbone had higher gene transfer to CD34^+^ cells than the SS and DS vectors across a range of vector concentrations (Figure 2C).

### *In vitro* assessment of the LVV using human SCD patient CD34^+^ cells

**Figure 3:**
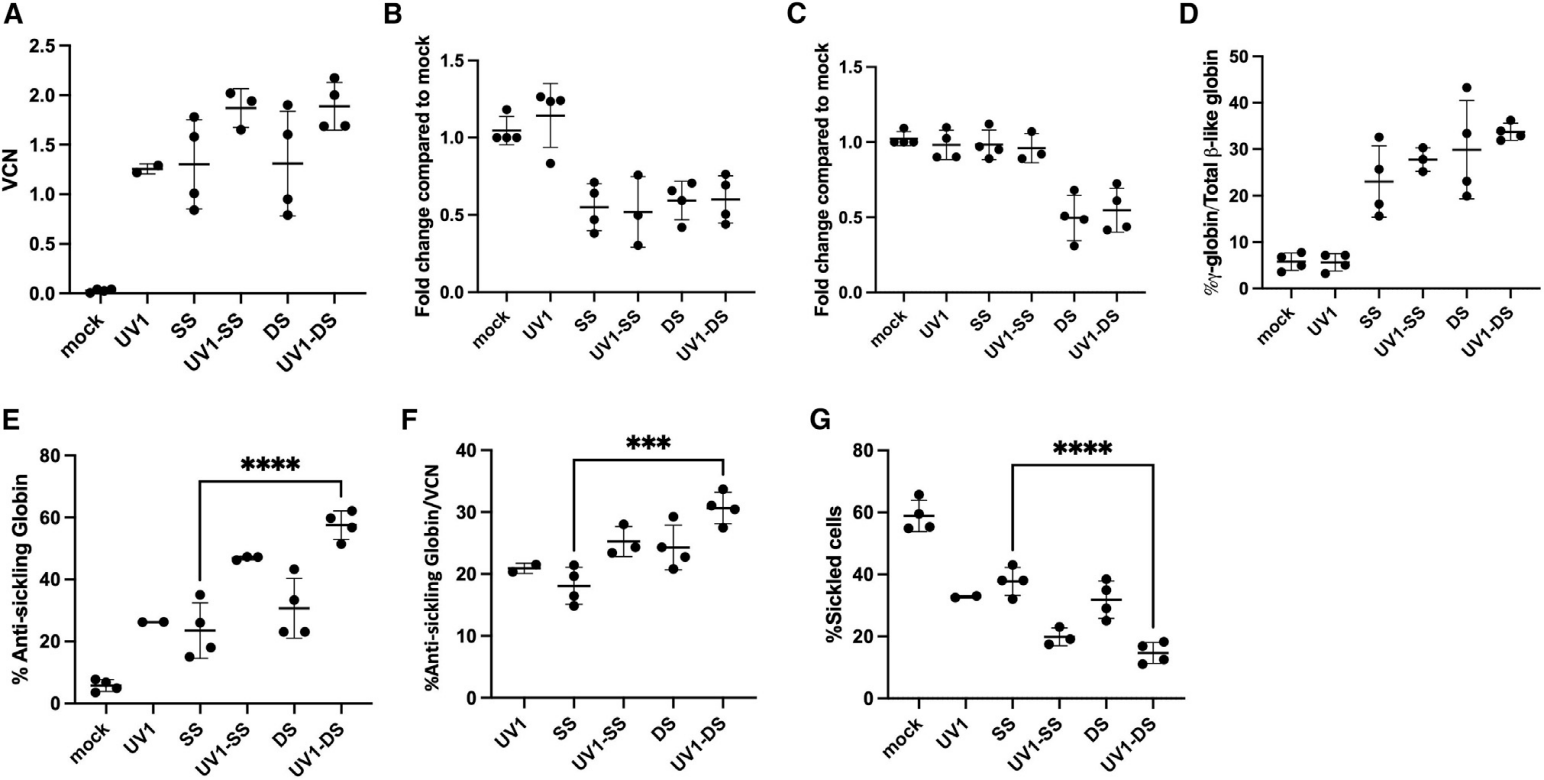
*In vitro* assessment of LVVs using human CD34^+^ cells derived from SCD patients (A) Plerixafor-mobilized CD34^+^ HPSCs from patients with SCD were transduced with vectors at 1 × 10^7^ TU/mL and then differentiated *in vitro* for 18 days in erythroid culture conditions. VCN of transduced cells were determined by qRT-PCR. (B) Expression was measured by qRT-PCR with GAPDH as control on day 11 of differentiation for *BCL11A* and (C) *ZNF410*. (D) Induction of gamma-globin mRNA and (E) anti-sickling globin (HBB^AS3^ + HBB) was determined on day 18 of differentiation by qRT-PCR. (F) Induction of anti-sickling globin (HBB^AS3^ + HBB) normalized to VCN (G) Enucleated red blood cells were enriched by fluorescence-activated cell sorting and treated with MBS to induce sickling. Quantification of % sickled cells of enucleated erythroid cells differentiated from transduced mock vector (control) and various test vectors was assessed by phase contrast microscopy 30 min after MBS treatment. Error bars represent means ± SD; each data point represents data from cells of an individual SCD patient; Tukey’s multiple comparisons test was performed on all arms with selected statistics shown. ∗∗∗*p* < 0.001, ∗∗∗∗*p* < 0.0001.

To evaluate the potential therapeutic impact of this series of LVVs in SCD patients, peripheral blood (PB) CD34^+^ cells from four different SCD donors were transduced with the five vectors at an MOI of 50. These cells were then subsequently differentiated *in vitro* using erythroid cytokines for 18 days after transduction. VCNs were determined by qPCR, anti-sickling HGB expression was determined by HPLC, and sickled cells were enumerated by microscopy after sodium metabisulfite (MBS) treatment. The VCNs generated with this MOI were in an appropriate range for comparison in subsequent analyses, with the VCNs of all experimental arms between an average of 1.3 ± 0.1 to 1.9 ± 0.2 (Figure 3A). To assess the functionality of UV1-DS in retaining the properties of *BCL11A* shmiR and *ZNF410* shmiR, we examined their knockdown efficiency by evaluating mRNA expression levels. As shown in Figures 3B and 3C, UV1 had no impact on the expression of *BCL11A* and *ZNF410*. In contrast, the groups that included *BCL11A* shmiR (UV1-SS and SS) effectively suppressed *BCL11A* expression without affecting *ZNF410*. Notably, the groups incorporating both *BCL11A* and *ZNF410* shmiRs (UV1-DS and DS) exhibited a significant decrease in the expression of both *BCL11A* and *ZNF410*.

Each of the vectors induced expression of anti-sickling hemoglobins; the UV1-DS vector induced the highest levels of anti-sickling HGB (HbA^AS3^ and HbF) at an average of 57.5% ± 4.6%, while the UV1, SS, UV1-SS, and DS vectors induced anti-sickling globin expression at an average of 26.3% ± 0.1%, 23.5% ± 9.0%, 46.9% ± 0.6%, and 30.7% ± 9.7% respectively (Figure 3B). When normalized to the VCN, the UV1-DS vector induced the highest anti-sickling HGB expression per VCN (30.7% ± 2.5%), which was significantly higher compared with SS (18.1% ± 3.0%) (Figure 3C).

Sodium MBS treatment of transduced enucleated erythroid cells was associated with decreased percentages of sickled erythrocytes with all vectors tested compared with non-transduced (“mock”) controls. Cells treated with UV1-DS showed the fewest sickled cells at an average of 14.6% ± 3.4%, while the average sickled cells in mock, UV1, SS, UV1-SS, DS were 58.8% ± 5.0%, 32.7% ± 0.4%, 37.8% ± 4.5%, 19.8% ± 2.9%, and 31.8% ± 6.0%, respectively (Figure 3D). Taken together, these data demonstrate that incorporating a DS vector in the UV1 backbone is feasible, further enhances anti-sickling HGB concentrations, and largely mitigates the cellular phenotype of erythrocyte sickling.

### *In vivo* analysis of PB from Berkeley SCD mouse model

To determine whether the UV1-DS vector can ameliorate characteristic SCD disease cellular phenotypes *in vivo*, we utilized a transplantation model with Berkeley SCD (BERK-SCD) mouse HSPCs as donor cells (Figure S1). We designed a UV1-DS(m) for murine BM cells, replacing the *ZNF410* shmiR with a *Zfp410* shmiR. *Zfp410* shmiR targeted the murine sequence and transduction led to the knock down of *Zfp410* and induction of Hbb-y mRNA expression in mouse erythroid leukemia cells (Figure S2). We conducted a direct comparison of UV1-DS, UV1-SS, UV1, DS, and SS vectors at equivalent VCN after transduction of lin^−^ CD45.2^+^ BM cells from BERK-SCD mice. Lin^−^ CD45.2^+^ BM cells from BERK-SCD mice were pre-stimulated for 36–40 h and were transduced with vectors at different MOIs to achieve a VCN of 2 (based on prior assays). The transduced cells were injected into lethally irradiated (11 Gy) CD45.^1^+ BL/6 (B6.SJL-Ptprca Pepcb/BoyJ) recipient animals 24 h after transduction. Two independent experiments were performed.

Engraftment was determined by flow cytometric enumeration of CD45.2^+^ donor cells; HGB, hematocrit (HCT), and reticulocyte concentrations were measured on PB samples and the frequencies of CD71^+^Ter119^+^ erythroid precursors were measured by flow cytometry. The percentages of sickled RBCS were determined after sodium MBS treatment of blood samples *ex vivo*. PB was collected at weeks 4, 8, 12, and 16 weeks after transplantation (Figure S3).

**Figure 4:**
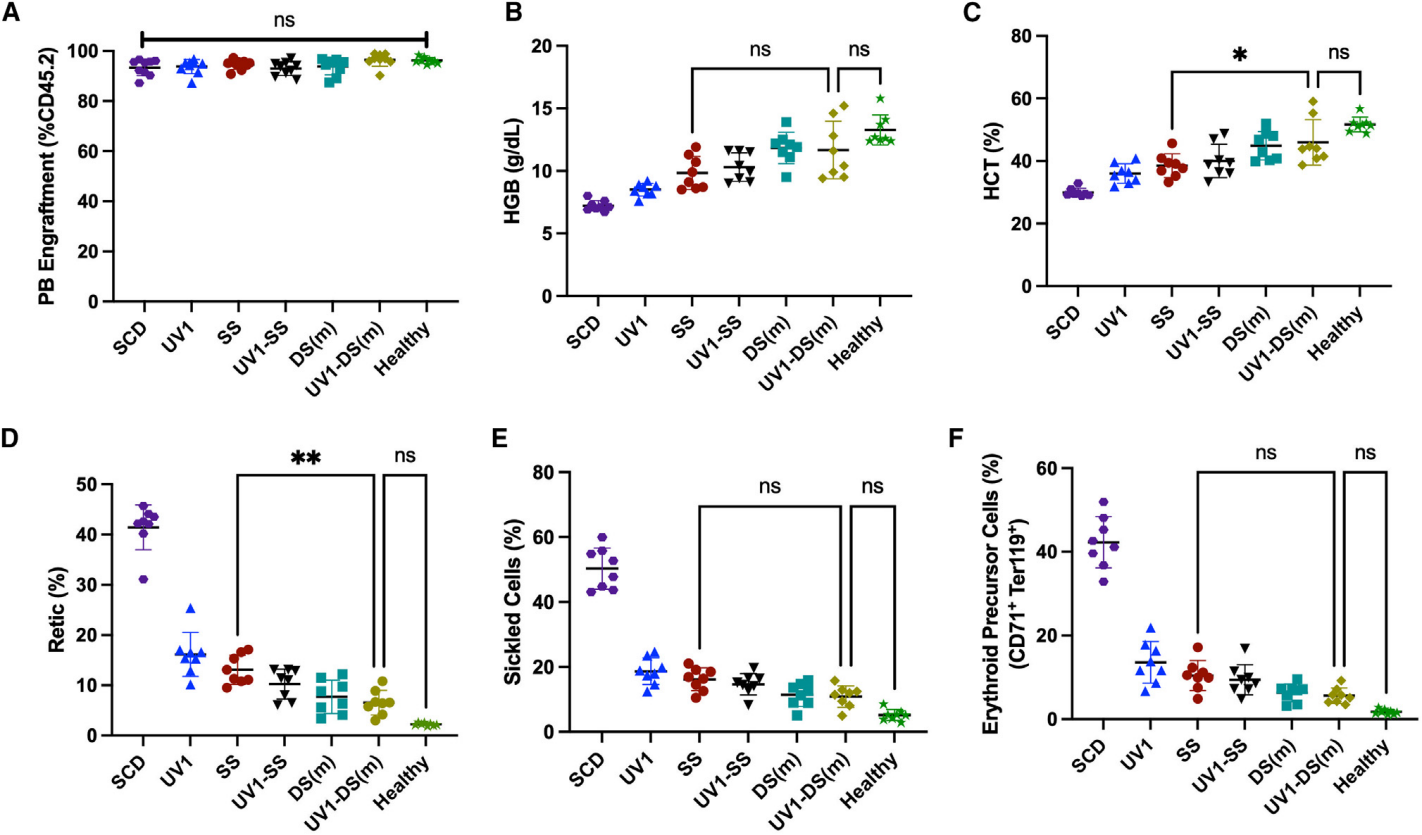
Correction of PB sickle cell hematologic parameters *in vivo* in Berkeley SCD mouse model lin^−^ bone marrow cells from BERK mice (CD45.2) were transduced with each vector at MOIs adjusted to achieve similar VCN, or mock-transduced as controls, and transplanted into irradiated CD45.1^+^ BL/6 mouse recipients. Mice were bled at 16 weeks after transplant and PB was analyzed. (A) Engraftment was assessed in PB by flow cytometry (%CD45.2^+^ cells). (B) HGB (g/dL), (C) HCT, and (D) reticulocyte counts (%) are shown. (E) PB was treated *ex vivo* with MBS for 30 min to induce sickling. Percentage of sickled RBCs from PB sample was quantified. (F) Percentages of CD71^+^ Ter119^+^ high erythroid precursor cell population in PB. Error bars represent mean ± SD. Symbols indicate mice transplanted with different shmiR vectors or non-transduced cells (SCD); each data point represents an individual mouse, n = 8, ns, not significant; Tukey’s multiple comparisons test was performed on all arms with selected statistics shown. ∗*p* < 0.05; ∗∗*p* < 0.01.

Engraftment across all experimental arms was greater than 87% and did not significantly differ between different vectors (Figure 4A). The HGB and HCT levels at 16 weeks were not significantly different when recipients of UV1-DS transduced cells were compared with the healthy donor arm (Figures 4B and 4C). The HGB of the UV1-DS recipient mice averaged 11.7 ± 2.3 g/dL, while the HCT of the SS, UV1, and SCD arms averaged 9.8 ± 1.3 g/dL, 8.5 ± 0.5 g/dL, and 7.2 ± 0.4 g/dL, respectively. The HCT of the UV1-DS recipient mice averaged 46.0 ± 7.2%, while the HCT of the SS, UV1, and SCD arms averaged 38.5 ± 3.8%, 36.0 ± 3.1%, and 30.0 ± 1.4%, respectively.

As a sensitive indicator of hemolysis, we measured reticulocyte and erythroid precursor cell populations in the PB. The reticulocyte percentages and the frequency of erythroid precursor cells of the UV1-DS experimental arm (6.5% ± 2.4%, 5.6% ± 1.9%) showed no statistically significant difference (*p* = 0.14, *p* = 0.39) with the healthy control (2.3% ± 0.3%, 1.7% ± 0.6%) (Figures 4D and 4F). The UV1-DS arm showed a significant decrease (*p* < 0.01) in reticulocytes in comparison to the SS arm (6.5% ± 2.4% vs. 13.1% ± 2.9%) (Figure 4D).

*Ex vivo* quantification of sickled cells in blood harvested from mice at 16 weeks was performed. Red blood cells were exposed to 2% sodium MBS and incubated under hypoxic conditions at 37°C for 30 min and imaged. Mice transplanted with UV1-DS transduced cells showed an average of 10.9% ± 3.3% sickled cells, which was not statistically significantly different (*p* = 0.07) compared with the healthy control arm with an average of 5.2% ± 1.7% abnormally shaped cells (Figure 4E). These values were obtained with an average VCN of 1.5 cdg. Overall, PB analysis suggested that the UV1-DS recipient animals demonstrated some significant improvement in hematologic parameters compared with the SS group, with no significant differences compared with animal transplanted with control cells from healthy donors.

### Analysis of bone marrow after gene therapy in the Berkeley SCD mouse model

**Figure 5:**
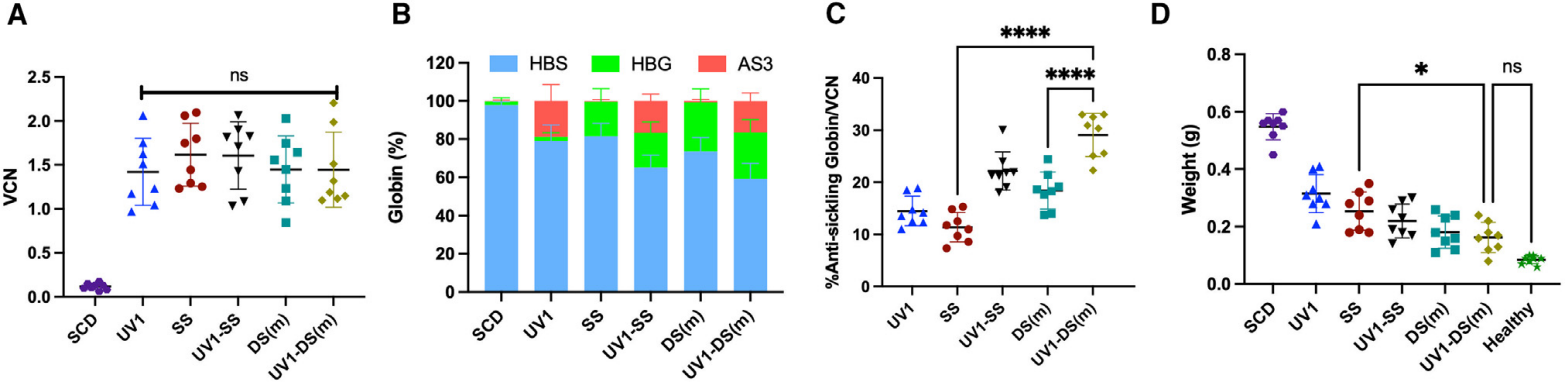
Anti-sickling HGB induction in bone marrow *in vivo* in the Berkeley mouse model Mice were euthanized at 16 weeks after transplant and whole bone marrow (BM) and spleen was harvested and analyzed individually. (A) VCN in BM was determined by qPCR. (B) Percentages of globin mRNA expression of erythroid cells in BM determined by qRT-PCR. (C) Anti-sickling globin mRNA expression in erythroid cells in BM adjusted for VCN. (D) Spleen weights. Error bars represent mean ± SD. Each data point represents an individual mouse. ns, not significant; Tukey’s multiple comparisons test was performed on all arms with selected statistics shown. ∗*p* < 0.05, ∗∗∗∗*p* < 0.0001.

Animals were sacrificed at 16 weeks for complete analyses, including BM and spleen studies. The average BM VCNs were similar among all of the experimental arms with a median of 1.5 VCN/diploid genome and a range of 0.5 (Figure 5A).

To determine if UV1-DS demonstrated therapeutic levels of anti-sickling HGB expression in BM cells, we measured β^AS3^-globin and γ-globin RNA transcript levels by RT-qPCR in erythroid cells derived from BM purified by flow cytometry. We confirmed the SS and DS groups demonstrated increased γ-globin, while the UV1 group expressed β^AS3^-globin and UV1-SS and UV1-DS induced both γ-globin and expressed β^AS3^-globin (Figure 5B). The total anti-sickling globin mRNA content (γ-globin + β^AS3^-globin) was determined, and the expression was normalized to the VCN in BM for each mouse. The highest level of anti-sickling globin mRNA expression was seen the UV1-DS group with an average of 29.0% ± 4.2% anti-sickling globin per 1 VCN/dg This expression was significantly higher than the DS and SS group at 18.4% ± 3.5% and 11.4% ± 2.8%, respectively (*p* < 0.0001) (Figure 5C).

Spleen weights were measured as an indication of compensatory erythroid expansion. The UV1-DS treated group showed an average spleen mass of 0.16 ± 0.05 g and was not significantly different than the healthy control arm of 0.09 ± 0.01 g. UV1-DS-treated spleen mass were significantly lower than the SS (*p* < 0.05) group at 0.25 ± 0.07 g (Figure 5D). Taken together, these data demonstrate that UV1-DS transduced cells led to a robust rescue of all SCD RBC phenotypes examined.

## Discussion

Gene therapies for SCD have made excellent advances in the past decade, with approaches using LVVs and CRISPR-Cas9 showing excellent clinical efficacy. A major limitation of gene therapy for SCD is the availability and access to a one-time potentially curative therapy for patients. With the cost of treatment being high ($1–$3 million per treatment) and the future reimbursement strategy for these expensive therapies not being clear, many individuals will face challenges in accessing approved therapies. One major factor is the high costs to produce LVVs that are produced at low titer and require a relatively high MOI for effective transduction of CD34^+^ HSPCs. The results presented here demonstrate the advantages in combining two technologies not only leading to increased efficacy, but also to increased titers and gene transfer, which may lead to lower production costs providing more accessible therapies.

Brendel et al.^21^ previously showed high induction of fetal globin expression per copy number with a *BCL11A* shmiR, while Morgan et al.^9^ showed that engineering smaller β^AS3^-globin vectors can lead to higher titers. We wanted to investigate whether combining shmiR and β^AS3^-globin technologies could lead to a superior therapy that had the combined benefits of potent anti-sickling globin expression with significantly increased titers and gene transfer. We first showed proof of concept with the incorporation of the *BCL11A* shmiR into the UV1 backbone. shmiRs integrated into the intronic regions of the β^AS3^-globin cassette had minimal impact on β^AS3^-globin protein expression, while incorporation into the 3′UTR region led to diminished expression of β^AS3^-globin. We hypothesize that, when the shmiR is processed out from the transcript in the 3′UTR, the transcript is degraded, thereby decreasing the amount of β^AS3^-globin expression. Since the other shmiRs are located in intronic regions, the introns will be spliced out before translation and we hypothesize that this is before the shmiR will be processed out, therefore having minimal impact on the β^AS3^-globin transcript. With the addition of the *BCL11A* shmiR, we witnessed fetal globin induction leading to an increase of total anti-sickling hemoglobins with the combination vectors. We did see that the incorporation of the shmiR led to a small decrease in gene transfer when transduced at equal transduction units in comparison with UV1. We hypothesized that the incorporation of the shmiR, which increases the length and complexity of the vector, could be leading to an increase of incomplete viral genomic RNAs. This concept has been previously studied, and the data have shown that β-globin LVV viral genomic RNAs can be incomplete and released in vector particles, leading to lower gene transfer in HSPCs.^8^ We extracted viral RNA from UV1 and the UV1-shmiR unconcentrated viral supernatants and performed a ddPCR assay to assess the concentration of complete viral RNAs.^8^ The data show a lower concentration of complete viral RNA in the UV1-shmiR vectors, which could explain lower gene transfer (Figure S4).

We formulated a hypothesis suggesting that shmiRs present in UV1 would exhibit greater titer and gene transfer than in their original backbones. Upon evaluating the titer and gene transfer of the UV1-DS vector, we found both to be comparable with those of the UV1 control. This indicates that the additional shmiR sequence did not significantly affect titer and gene transfer. Moreover, the variation in backbone sequence of UV1, characterized by its smaller size and different complexity, yielded advantages in terms of higher titer and gene transfer, compared with SS and DS in a longer backbone. Additionally, UV1-DS also showed a 2- to 8-fold increase in titer when packaged and titered head to head with alternative β^AS3^-globin LVVs^11^^,^^31^ (Figure S5). The UV1 backbone has a substantial benefit because lower transduction concentrations can be used to achieve the same VCN, meaning lower viral volumes will be needed, leading to less potential toxicity for the cells and reduced cost per patient dose. For example, a 5,00.-fold scale up from 1 × 10^5^ to 5 × 10^8^ for a hypothetical 75-kg patient and a CD34^+^ cell dose of 6 × 10^6^/kg would require the DS vector produced from 4 L for one patient dose versus 0.2 L for the UV1-DS vector; thus UV1-DS would yield 20-fold more patient doses per vector lot.

A crucial aspect in the development of this therapy involved evaluating whether the combination would result in improved efficacy. Previous research has demonstrated that achieving approximately 20% expression of anti-sickling hemoglobins leads to an improvement in the SCD phenotype.^32^ Based on this, our hypothesis was that the UV1-DS vector would exhibit the highest expression of anti-sickling hemoglobins by combining β^AS3^-globin expression and γ-globin induction, potentially allowing for a lower VCN requirement to ameliorate the disease. To evaluate the efficacy of the UV1-DS vector, we utilized human SCD CD34^+^ cells and conducted erythroid differentiation. We observed the highest induction of anti-sickling hemoglobins in UV1-DS-treated cells (approximately 30% per VCN), resulting in a significant decrease in the sickling phenotype when compared with the UV1, SS, or DS vectors. Based on this experiment we would expect that human SCD CD34^+^ cells transduced with UV1-DS at VCN of 1 would achieve therapeutic levels. This finding supports the notion that the combination therapy holds promise for effectively addressing SCD at a lower VCN, which in turn can lead to fewer safety concerns regarding insertional mutagenesis. In contrast, the inclusion of multiple shmiRs may increase the chance of off-target effects. Through sequence homology analysis, the probability of BCL11A and ZNF410 shmiRs having off-target effects seems to be low (data not shown). We previously reported differentially expressed genes after BCL11A and ZNF410 knockdown in erythroid cells^13^^,^^21^^,^^27^ (Figure S6), but were unable to identify any undesirable side effects on cellular erythroid differentiation or cell physiology *in vitro* or *in vivo.*

We then assessed amelioration of hematologic parameters of the sickle phenotype in the BERK SCD mouse model. We excluded mice with less than 97% donor engraftment at week 16 from the analysis to prevent confounding of phenotype correction by residual wild-type erythrocytes. We made the decision to perform *in vivo* experimentation at equal VCN to allow for intrinsic anti-sickling activity of each vector to be determined. However, using different amounts of each vector to achieve a similar VCN obscures the potential advantages of smaller, higher titer vectors to transduce higher percentages of cells and achieve sufficient VCNs to impede sickling. Of note, experimental arms using LVV in the UV1 backbone required less vector volume for transduction to achieve the same VCN as the shmiR only vectors in a longer backbone (SS and DS), which suggests not only less potential cell toxicity when translated clinically, but also decreased the cost of therapy. All hematologic parameters assessed in the PB of recipients treated with UV1-DS showed significant improvements when compared with mock-treated and UV1-treated lineage-negative (lin^−^) cells. Significant improvements in HCT and reticulocyte percentages were also seen when compared with the SS arm. When assessing BM, there was a significant increase in percentages of anti-sickling hemoglobins per VCN, as well as a significant decrease in the spleen weights of the UV1-DS-treated group in comparison with the UV1 and SS arms. These data emphasize that the UV1-DS vector has potential to be advantageous in a clinical setting in comparison with β^AS3^-globin and shmiR technologies separately.

We also performed *in vivo* experimentation in the Townes SCD mouse model (Figures S7–S9), but saw minimal to no induction of HbF in mice treated with vectors containing shmiRs. A recent article that has characterized the Townes mouse model by long-range sequencing showed the lack of distal gene-regulatory elements that may be necessary for HbF induction.^33^ Several human assay for transposase-accessible chromatin sequencing (ATAC-seq) signals seen in the human β-globin locus were not included the Townes mouse transgenes and are also not conserved in mice. These elements may be involved with HGB switching, a mechanism that does not naturally occur in mice, leading to the potential suboptimal expression of the *HBG1* transgene.^33^ In addition, human ATAC-seq peaks for known gamma-globin regulators were also not seen in the Townes mouse transgene including: *HBBP1*^34^ *and BGLT3*.^35^^,^^33^ While *Bcl11a* and *Zfp410* may have been successfully downregulated in our Townes model, we hypothesize that lack of key regulatory elements may be the reason we witnessed minimal HbF induction. Our results suggest that the Townes mouse is not an optimal model when assessing HbF induction by *BCL11A* and *Zfp410* shmiRs.

The incorporation of shmiRs into the UV1 backbone has yielded a vector that not only induces high anti-sickling HGB expression, but also exhibits higher titer and gene transfer capabilities, compared with earlier constructs. It is worth noting that previous clinical trials involving β^AS3^-globin and *BCL11A* shmiR technology have shown considerable success, but by utilizing the UV1-DS vector, we can capitalize on the remarkable efficacy observed with shmiR technology, while simultaneously maintaining the high titers and gene transfer capabilities associated with the UV1 vector. Consequently, UV1-DS holds the potential for significant advantages in terms of efficacy, clinical scale production, and cost reduction for autologous gene therapy. These promising features make UV1-DS an exciting prospect for further exploration and potential implementation in future therapeutic approaches.

## Materials and methods

### Cloning and vector production

The UV1 vector, *BCL11A* shmiR, and *ZNF410* shmiR, *Zfp410* shmiR have been described previously.^9^^,^^13^^,^^21^^,^^27^ To introduce the BCL11A shmiR, five sets of reverse-oriented primers with extended homology sequence were used to PCR amplify the UV1 plasmid backbone. The *BCL11A* shmiR oligos (Integrated DNA Technologies, San Diego, CA, USA) were combined and an oligo duplex was generated. With homology between the shmiR sequences and linearized plasmids, the shmiR sequences were joined using the NEBuilder HiFi DNA Assembly kit (New England Biolabs, Ipswich, MA, USA). All plasmids were sequence verified by Sanger sequencing (Laragen Inc, Culver City, CA, USA).

HEK293T *PKR* knockout cells^8^ were plated on 10-cm plates at a density of 1 × 10^7^ cells/mL and vectors were packaged through transient transfection, using third-generation lentiviral packaging plasmids.^36^ Raw viral supernatant was collected 3 days after transfection, samples were used for titer determination, and the remainder of the viral supernatants were concentrated through ultracentrifugation. Titers were determined by performing transduction of HT-29 human colorectal carcinoma cell line at multiple dilutions of both raw and concentrated viral supernatant. Three days after transduction, cells were harvested and titers were calculated through VCN determination by ddPCR assay using primers and probes for HIV-1 PSI (forward 5′-AAGTAGTGTGTGCCCGTCTG-3′, reverse 5′-CCTCTGGTTTCCCTTTCGCT-3′, 5′-56-FAM-AGCTCTCTC-ZEN-GACGCAGGACTCGGC-3IABkFQ-3′) and the Human Syndecan 4 gene (SCD4) as a reference (forward 5′-CAGGGTCTGGGAGCCAAGT-3′, reverse 5′-GCACAGTGCTGGACATTGACA-3′, 5′-5HEX-CCCACCGAA-ZEN-CCCAAGAAACTAGAGGAGAAT-3IABkFQ-3′).

### PB healthy CD34^+^ transduction and erythroid differentiation

PB CD34^+^ samples were obtained from healthy donors by plerixafor and granulocyte colony-stimulating factor (G-CSF) mobilization. Cells were thawed and plated at 1 × 10^6^ cells/mL on non-tissue culture treated 96 well plates pre-coated with retronectin (20 μg/mL, Takara Shuzo, Otsu, Japan). Cells were pre-stimulated for 24 h in X-Vivo 15 medium (Lonza, Basel, Switzerland) supplemented with 1× glutamine, penicillin, and streptomycin (Gemini Bio-Products, Sacramento, CA, USA), human Flt-3 ligand (50 ng/mL), human stem cell factor (SCF) (50 ng/mL), human thrombopoietin (TPO) (50 ng/mL), and human IL-3 (20 ng/mL) (cytokines: PeproTech, Rocky Hill, NJ, USA). CD34^+^ cells were transduced with concentrated viral supernatants at a transduction concentration of 2 × 10^7^ TU/mL (MOI of 20), without additional transduction enhancers. At 24 h after transduction, the cells were washed and plated under erythroid culture conditions. At days 2–7 after transduction, the cells were cultured in erythroid differentiation base medium (EDM) consisting of Iscove modified Dulbecco’s medium (Lonza), 1× glutamine, penicillin, and streptomycin (Gemini Bio-Products), Holo-human transferrin (330 μg/mL) (Sigma-Aldrich, Burlington, MA), recombinant human insulin (10 μg/mL) (Sigma-Aldrich), heparin (2 IU/mL) (Sigma-Aldrich), 5% human solvent detergent pooled plasma AB (Octapharma USA Inc., Paramus, NJ, USA) supplemented with hydrocortisone (1 μM) (Sigma-Aldrich), human IL-3 (5 ng/mL) (PeproTech), human SCF (100 ng/mL) (PeproTech), and erythropoietin (3 IU/mL) (Sigma-Aldrich). At days 8–10 after transduction, the cells were cultured in EDM supplemented with 3 IU/mL erythropoietin. At days 11–21 after transduction, the cells were cultured in EDM without added cytokines. Cells were collected 14 days after transduction and ddPCR assays were used to analyze VCN and mRNA expression for γ-globin and β^AS3^-globin. At day 21, cells were collected for protein analysis using HPLC to assess adult HGB (HbA), HbF, and β^AS3^-globin (Hbβ^AS3^).

### Myeloid dose response

PB CD34^+^ samples were obtained from healthy donors from plerixafor and G-CSF mobilization. Cells were thawed and plated at 1 × 10^6^ cells/mL on non-tissue culture-treated 96-well plates pre-coated with retronectin (20 μg/mL; Takara Shuzo). Cells were pre-stimulated for 24 h in X-Vivo 15 medium (Lonza) supplemented with 1× glutamine, penicillin, and streptomycin (Gemini Bio-Products), human Flt-3 ligand (50 ng/mL), human SCF (50 ng/mL), human TPO (50 ng/mL), and human IL-3 (20 ng/mL) (cytokines: PeproTech). CD34^+^ cells were transduced with concentrated viral supernatants at transduction concentrations of 2 × 10^5^ TU/mL, 6 × 10^5^ TU/mL, 2 × 10^6^ TU/mL, and 6 × 10^6^ TU/mL with transduction enhancer Poloxamer 338 (1 mg/mL) (BASF, Ludwigshafen, Germany). At 24 h after transduction, cells were washed and plated under myeloid culture conditions. Cells were cultured for 2 weeks after transduction in basal bone marrow media consisting of Iscove-modified Dulbecco’s medium (Lonza) 1× glutamine, penicillin, and streptomycin (Gemini Bio-Products), 20% fetal bovine serum, and 0.52% BSA (Sigma-Aldrich) supplemented with human SCF (25 ng/mL), human IL-3 (5 ng/mL), and human IL-6 (10 ng/mL).

### Transduction of human SCD patient CD34^+^ cells

SCD patient CD34^+^ HSPCs were isolated from unmobilized PB following receiving Boston Children’s Hospital institutional review board approval and informed patient consent. The SCD CD34^+^ HSPCs were enriched using the Miltenyi CD34 Microbead kit (Miltenyi Biotec, Auburn, CA, USA). CD34^+^ cells were prestimulated for 36–40 h at 1 × 10^6^ cells/mL in Stem Cell Growth Medium (CellGenix, Portsmouth, NH, USA) supplemented with SCF, FMS-like tyrosinekinase 3 ligand, and TPO, all from Peprotech. Cells were then enumerated and transduced with the vector at an MOI as indicated in presence of LentiBOOST enhancer (SIRION Biotech, Gräfelfing, Germany) for 24 h before downstream processing.

### *In vitro* erythroid differentiation of SCD CD34^+^ cells

The *in vitro* erythroid differentiation protocol used is based on a three phase protocol adapted from Giarratana et al.^37^ The cells were cultured in erythroid differentiation medium (EDM) consisting of Iscove modified Dulbecco’s medium (Cellgro, Manassas, VA, USA) supplemented with 1% L-glutamine (Thermo Fisher Scientific, Waltham, MA, USA), and 1% penicillin-streptomycin (Thermo Fisher Scientific), 330 mg/mL holo-human transferrin (Sigma-Aldrich), 10 mg/mL recombinant human insulin (Sigma-Aldrich), 2 IU/mL heparin (Sigma-Aldrich), 5% human solvent detergent pooled plasma AB (Rhode Island Blood Center, Providence, RI, USA), and 3 IU/mL erythropoietin (Amgen, Thousand Oaks, CA, USA). During the first phase of expansion (days 0–7), CD34^+^ cells were cultured in EDM in the presence of 10^6^ mol/L hydrocortisone (Sigma-Aldrich), 100 ng/mL SCF (Peprotech), 5 ng/mL IL-3 (R&D Systems, Minneapolis, MN, USA), as EDM-1. In the second phase (days 7–11), the cells were resuspended in EDM supplemented with SCF, as EDM-2. For the third phase (days 11–18), the cells were cultured in EDM without additional supplements, as EDM-3.

### *In vitro* sickling assay

At the completion of erythroid differentiation, enucleated RBCs were sorted, with the use of Hoechst 33342 (5 mg/mL; Invitrogen, Waltham, MA, USA), and subjected to an *in vitro* sickling assay. Sickling was induced by adding 500 mL freshly prepared 2% sodium MBS (Sigma-Aldrich) solution prepared in PBS into enucleated cells resuspended with 500 mL EDM-3 in a 24-well plate, followed by incubation at 37°C for 30 min. Live cell images were acquired using a Nikon Eclipse Ti inverted microscope (Nikon, Tokyo, Japan). More than 500 cells were counted for each sample; cells with an irregular structure, protruding spikes, or sickle shape were counted as sickling cells.

### *In vivo* experiment in the SCD mouse model

Lin^−^ mouse BM cells were isolated by flushing femurs, tibias, and iliac crests of 6- to 8-week-old CD45.2 C57BL/6 or CD45.2 Berkeley SCD mice (BERK-SCD, JAX stock #003342) followed by lineage depletion using the Mouse Lineage Cell Depletion Kit (Miltenyi Biotec, Bergisch Gladbach, Germany). Lin− cells were pre-stimulated at 1 × 10^6^ cells/mL in Stem Cell Growth Medium (CellGenix) supplemented with mouse SCF (100 ng/mL), hTPO (100 ng/mL), mouse IL-3 (mIL-3) (20 ng/mL), and hFlt3-L (100 ng/mL), all from Peprotech. Following a 36- 40-h pre-stimulation, cells were transduced at a density of 1 × 10^6^ cells/mL in the presence of LentiBOOST enhancer, and transduced cells (without sorting) were transplanted by retro-orbital injection into lethally irradiated (7 + 4 Gy, split dose) CD45.1 recipients (B6.SJL-Ptprca Pepcb/BoyJ, Jax Strain #002014) 24 h after transduction. PB samples were collected at weeks 4, 8, 12, and 16 to measure engraftment by flow cytometry (CD45.2/CD45.1), determine RBC indices, and quantitate sickled cells. At week 16, mice were euthanized, and BM cells were used to measure engraftment by flow cytometry (CD45.2/CD45.1), VCN, and mRNA expression, spleens were collected to weigh. All animal experiments were approved by the Boston Children's Hospital's Institutional Animal Care and Use Committee.

### VCN assay for the BERK mouse studies

Genomic DNA was extracted using the QIAGEN DNeasy protocol. VCN was assessed by qRT-PCR, performed with the use of TaqMan Fast Advanced Master Mix (Applied Biosystems, Waltham, MA, USA). VCN was calculated by using primers and probes HIV-1 PSI (forward 5′-CAGGACTCGGCTTGCTGAAG-3′, reverse 5′-TCCCCCGCTTAATACTGACG-3′, probe FAM-50-CGCACGGCAAGAGGCGAGG-3′) as a target and the human glycosyltransferase Like Domain Containing 1 gene (GTDC1) as an internal reference standard (forward 5′-GAAGTTCAGGTTAATTAGCTGCTG-3′, reverse 5′-TGGCACCTTAACATTTGGTTCTG-3′, probe VIC-5′-ACGAACTTCTTGGAGTTGTTTGCT-3′). Standard curves were obtained by serial dilutions of a plasmid containing one copy of PSI and GTDC1 sequences. The number of PSI and GTDC1 copies in test samples was extrapolated from the standard curves.

## Data and code availability

Data available from Dr. Donald B. Kohn upon request.

## Supplemental information

Document S1. Figures S1–S9 and Tables S1 and S14

Document S2. Article plus supplemental information
