# Supplementary material for: A novel high-titer, bifunctional lentiviral vector for autologous hematopoietic stem cell gene therapy of sickle cell disease
Source: Mol Ther Methods Clin Dev. 2024 Apr 24;32(2):101254. doi: 10.1016/j.omtm.2024.101254 (PMC11091523; doi:10.1016/j.omtm.2024.101254)
Supplement: Document S1. Figures S1–S9 and Tables S1 and S14 [file mmc1.pdf]

OMTM, Volume *32*

Supplemental information

A novel high-titer, bifunctional lentiviral vector for autologous hematopoietic stem cell gene therapy of sickle cell disease

Kevyn L. Hart, Boya Liu, Devin Brown, Beatriz Campo-Fernandez, Kevin Tam, Katherine Orr, Roger P. Hollis, Christian Brendel, David A. Williams, and Donald B. Kohn

The Townes SCD mouse model shows poor induction of HbF.

We performed a study to assess the activity of the UV1-SS and UV1-DS vectors in the Townes mouse SCD murine model, which reproduce several hematologic and pathophysiologic aspects of clinical disease.[1,2] We first cloned fetal-globin positive control vectors that only express gamma-globin, by knocking out the β^{AS3}-globin open reading frame in the UV1-SS and UV1-DS vectors, creating UV1-SS (No AS3) and UV1-DS (No AS3). To generate the controls all methionine codons (ATG) in the β^{AS3}-globin open reading frame were mutated. Site-directed mutagenesis was used to change the ATG start codon as well as a downstream methionine codon in exon 2 (Figure S7A). The single or double shmiR^{s} were left unchanged.

We validated the fetal globin positive controls by performing an erythroid differentiation using the parental HUDEP-2 cell line. The cells were transduced with vectors at $3.0x10^{5}$ TU/mL without transduction enhancers to aim for a VCN of 1-2. The cells were differentiated into erythrocytes and were collected on day 7 for VCN analysis and on day 12 for protein. Protein was analyzed by HPLC (Figure S7B, S7C). The HPLC data shows that UV1-SS (No AS3) and UV1-DS (No AS3) do not express Hbβ^{AS3}, while HbF is induced, suggesting that β^{AS3}-globin has been knocked out and will be appropriate controls in the UV1 backbone.

The control vectors were packaged and concentrated virus was generated through ultra-centrifugation. Titer was determined for raw and concentrated supernatant through HT-29 transduction (Figure S7D). A myeloid dose response with Lin- cells was performed to determine which concentration to use for each vector (UV1, UV1-SS, UV1-DS, UV1-SS (No AS3), UV1-DS (No AS3)) to obtain a VCN around 4 for Townes mouse studies Figure S7E).

Lin- Townes mouse BM cells were pre-stimulated at $1x10^{6}$ cells/mL and transduced 24 hours later. 24 hours post transduction the cells were transplanted by retro-orbital injection into lethally irradiated B6 CD45.1 recipients. PB, BM, and spleen were collected at week 16 for analysis.

There was a small induction of HbF in red blood cells in mice treated with vectors containing the single or double shmiR^{s} after intracellular staining of HbF and flow cytometry analysis (Figure S8B). However, there were not significant differences with the hematologic indices or spleen weights in untransduced SCD recipients (Figure S8C).

These data demonstrate that the Townes mouse model does not induce HbF expression to a functional level when knocking down *Bcl11a* and *Zfp410* with shmiR^{s}, compared to the degree of HbF induction achieved in human HSPC. Due to minimal induction of HbF, we did not see correction of the erythroid manifestations of SCD in the Townes mouse model. Woodard et al.[3] observed a similar blunted induction of HbF in Townes mouse HSPC that were treated with CRISPR/Cas9 nuclease to disrupt gamma-globin gene promoter sites.

Table S1: *BCL11A* shmiR insertion sequences in the β^{AS3}-globin cassette.

ShmiR Location	DNA Insertion Site: shmiR Sequence + 20 Base Flanking Sequences
Start-IVS1	caagacaggtttaaggagac**GATCTCACTTCCCCACAGAAGCTCTTGGCCTGGCCTC**<br>**CTGCAGTGCCACGCTGCGCGATCGAGTGTTGAATAACTCCATGTGGTAGAG**<br>**TTATTCAACACTCGATCGCGCAGTGCGGCACATGCTTACCAGCTCTAGGCC**<br>**AGGGCAGATGGGATATGACGAATGGACTGCCAGCTGGATACAAGGATGCT**<br>**CACC**caatagaaactgggcatgtg
End-IVS1	actctctctgcctattggtc**GATCTCACTTCCCCACAGAAGCTCTTGGCCTGGCCTCCT**<br>**GCAGTGCCACGCTGCGCGATCGAGTGTTGAATAACTCCATGTGGTAGAGTT**<br>**ATTCAACACTCGATCGCGCAGTGCGGCACATGCTTACCAGCTCTAGGCCAG**<br>**GGCAGATGGGATATGACGAATGGACTGCCAGCTGGATACAAGGATGCTCA**<br>**CC**tattttcccacccttaggct
IntraΔ-IVS2	gaaggggagaagtaacaggg**GATCTCACTTCCCCACAGAAGCTCTTGGCCTGGCCT**<br>**CCTGCAGTGCCACGCTGCGCGATCGAGTGTTGAATAACTCCATGTGGTAGA**<br>**GTTATTCAACACTCGATCGCGCAGTGCGGCACATGCTTACCAGCTCTAGGC**<br>**CAGGGCAGATGGGATATGACGAATGGACTGCCAGCTGGATACAAGGATGC**<br>**TCACC**tatttctgcatataaattgt
End-IVS2	tttgctaatcatgttcatac**GATCTCACTTCCCCACAGAAGCTCTTGGCCTGGCCTCCTG**<br>**CAGTGCCACGCTGCGCGATCGAGTGTTGAATAACTCCATGTGGTAGAGTTA**<br>**TTCAACACTCGATCGCGCAGTGCGGCACATGCTTACCAGCTCTAGGCCAGG**<br>**GCAGATGGGATATGACGAATGGACTGCCAGCTGGATACAAGGATGCTCAC**<br>**C**ctcttatcttcctcccacag
3'UTR	tccaactactaaactggggg**GATCTCACTTCCCCACAGAAGCTCTTGGCCTGGCCTCC**<br>**TGCAGTGCCACGCTGCGCGATCGAGTGTTGAATAACTCCATGTGGTAGAGT**<br>**TATTCAACACTCGATCGCGCAGTGCGGCACATGCTTACCAGCTCTAGGCCA**<br>**GGGCAGATGGGATATGACGAATGGACTGCCAGCTGGATACAAGGATGCTC**<br>**ACC**atattatgaagggccttgag

Table S2: Tukey's multiple comparisons test of vector titers (Figure 2B).

Tukey's multiple comparisons test	Mean Diff.	95.00% CI of diff.	Below threshold?	Summary	Adjusted P Value
SS vs. DS	-55000	-339596 to 229596	No	ns	0.9624
SS vs. UV1	-4453333	-4771522 to -4135145	Yes	****	<0.0001
SS vs. UV1-SS	-3241667	-3526263 to -2957070	Yes	****	<0.0001
SS vs. UV1-DS	-3705000	-3989596 to -3420404	Yes	****	<0.0001
DS vs. UV1	-4398333	-4716522 to -4080145	Yes	****	<0.0001
DS vs. UV1-SS	-3186667	-3471263 to -2902070	Yes	****	<0.0001
DS vs. UV1-DS	-3650000	-3934596 to -3365404	Yes	****	<0.0001
UV1 vs. UV1-SS	1211667	893478 to 1529855	Yes	****	<0.0001
UV1 vs. UV1-DS	748333	430145 to 1066522	Yes	***	0.0002
UV1-SS vs. UV1-DS	-463333	-747930 to -178737	Yes	**	0.0027

Table S3: Tukey's multiple comparisons test of anti-sickling globin induction in human SCD CD34+ HSPCs (Figure 3B).

Tukey's multiple comparisons test	Mean Diff.	95.00% CI of diff.	Below threshold?	Summary	Adjusted P Value
mock vs. UV1	-20.47	-38.20 to -2.734	Yes	*	0.0195
mock vs. SS	-17.72	-32.20 to -3.239	Yes	*	0.0126
mock vs. UV1-SS	-41.12	-56.76 to -25.48	Yes	****	<0.0001
mock vs. DS	-24.94	-39.42 to -10.46	Yes	***	0.0006
mock vs. UV1-DS	-51.73	-66.21 to -37.25	Yes	****	<0.0001
UV1 vs. SS	2.750	-14.98 to 20.48	No	ns	0.9952
UV1 vs. UV1-SS	-20.65	-39.34 to -1.957	Yes	*	0.0264
UV1 vs. DS	-4.467	-22.20 to 13.27	No	ns	0.9595
UV1 vs. UV1-DS	-31.26	-49.00 to -13.53	Yes	***	0.0005
SS vs. UV1-SS	-23.40	-39.04 to -7.760	Yes	**	0.0023
SS vs. DS	-7.216	-21.70 to 7.264	No	ns	0.5994
SS vs. UV1-DS	-34.01	-48.49 to -19.53	Yes	****	<0.0001
UV1-SS vs. DS	16.18	0.5434 to 31.82	Yes	*	0.0405
UV1-SS vs. UV1-DS	-10.61	-26.25 to 5.030	No	ns	0.2914
DS vs. UV1-DS	-26.79	-41.27 to -12.31	Yes	***	0.0003

Table S4: Tukey’s multiple comparisons test of anti-sickling globin induction normalized to VCN in human SCD CD34+ HSPCs (Figure 3C).

Tukey's multiple comparisons test	Mean Diff.	95.00% CI of diff.	Below threshold?	Summary	Adjusted P Value
UV1 vs. SS	2.820	-5.048 to 10.69	No	ns	0.7819
UV1 vs. UV1-SS	-4.329	-12.62 to 3.966	No	ns	0.4890
UV1 vs. DS	-3.368	-11.24 to 4.501	No	ns	0.6596
UV1 vs. UV1-DS	-9.740	-17.61 to -1.871	Yes	*	0.0136
SS vs. UV1-SS	-7.149	-14.09 to -0.2093	Yes	*	0.0425
SS vs. DS	-6.188	-12.61 to 0.2364	No	ns	0.0610
SS vs. UV1-DS	-12.56	-18.98 to -6.135	Yes	***	0.0003
UV1-SS vs. DS	0.9604	-5.979 to 7.900	No	ns	0.9911
UV1-SS vs. UV1-DS	-5.411	-12.35 to 1.528	No	ns	0.1584
DS vs. UV1-DS	-6.372	-12.80 to 0.05328	No	ns	0.0523

Table S5: Tukey's multiple comparisons test of sickled cells quantification in human SCD CD34+ HSPCs (Figure 3D).

Tukey's multiple comparisons test	Mean Diff.	95.00% CI of diff.	Below threshold?	Summary	Adjusted P Value
mock vs. UV1	26.08	13.57 to 38.58	Yes	****	<0.0001
mock vs. SS	21.08	10.87 to 31.28	Yes	****	<0.0001
mock vs. UV1-SS	38.99	27.97 to 50.02	Yes	****	<0.0001
mock vs. DS	27.00	16.79 to 37.21	Yes	****	<0.0001
mock vs. UV1-DS	44.20	33.99 to 54.41	Yes	****	<0.0001
UV1 vs. SS	-5.000	-17.50 to 7.502	No	ns	0.7810
UV1 vs. UV1-SS	12.92	-0.2612 to 26.09	No	ns	0.0563
UV1 vs. DS	0.9250	-11.58 to 13.43	No	ns	0.9999
UV1 vs. UV1-DS	18.13	5.623 to 30.63	Yes	**	0.0031
SS vs. UV1-SS	17.92	6.891 to 28.94	Yes	**	0.0011
SS vs. DS	5.925	-4.283 to 16.13	No	ns	0.4463
SS vs. UV1-DS	23.13	12.92 to 33.33	Yes	****	<0.0001
UV1-SS vs. DS	-11.99	-23.02 to -0.9662	Yes	*	0.0294
UV1-SS vs. UV1-DS	5.208	-5.817 to 16.23	No	ns	0.6491
DS vs. UV1-DS	17.20	6.992 to 27.41	Yes	***	0.0007

Table S6: Tukey's multiple comparisons test of engraftment of lineage negative bone marrow from BERK mouse (CD45.2) (Figure 4A).

Tukey's multiple comparisons test	Mean Diff.	95.00% CI of diff.	Below threshold?	Summary	Adjusted P Value
SCD vs. UV1	-0.3799	-4.213 to 3.453	No	ns	>0.9999
SCD vs. SS	-1.178	-5.011 to 2.655	No	ns	0.9642
SCD vs. UV1-SS	0.4108	-3.422 to 4.244	No	ns	0.9999
SCD vs. DS	-0.3919	-4.225 to 3.441	No	ns	>0.9999
SCD vs. UV1-DS	-3.110	-6.943 to 0.7231	No	ns	0.1860
SCD vs. Healthy	-2.831	-6.664 to 1.002	No	ns	0.2824
UV1 vs. SS	-0.7985	-4.632 to 3.035	No	ns	0.9952
UV1 vs. UV1-SS	0.7908	-3.042 to 4.624	No	ns	0.9955
UV1 vs. DS	-0.01198	-3.845 to 3.821	No	ns	>0.9999
UV1 vs. UV1-DS	-2.730	-6.563 to 1.103	No	ns	0.3237
UV1 vs. Healthy	-2.451	-6.284 to 1.382	No	ns	0.4539
SS vs. UV1-SS	1.589	-2.244 to 5.422	No	ns	0.8634
SS vs. DS	0.7865	-3.047 to 4.620	No	ns	0.9956
SS vs. UV1-DS	-1.932	-5.765 to 1.902	No	ns	0.7192
SS vs. Healthy	-1.652	-5.485 to 2.181	No	ns	0.8406
UV1-SS vs. DS	-0.8028	-4.636 to 3.030	No	ns	0.9951
UV1-SS vs. UV1-DS	-3.521	-7.354 to 0.3122	No	ns	0.0916
UV1-SS vs. Healthy	-3.242	-7.075 to 0.5915	No	ns	0.1500
DS vs. UV1-DS	-2.718	-6.551 to 1.115	No	ns	0.3288
DS vs. Healthy	-2.439	-6.272 to 1.394	No	ns	0.4599
UV1-DS vs. Healthy	0.2792	-3.554 to 4.112	No	ns	>0.9999

Table S7: Tukey's multiple comparisons test of hemoglobin in BERK SCD mouse model (Figure 4B).

Tukey's multiple comparisons test	Mean Diff.	95.00% CI of diff.	Below threshold?	Summary	Adjusted P Value
SCD vs. UV1	-1.288	-3.280 to 0.7048	No	ns	0.4360
SCD vs. SS	-2.625	-4.617 to -0.6327	Yes	**	0.0032
SCD vs. UV1-SS	-3.100	-5.092 to -1.108	Yes	***	0.0003
SCD vs. DS	-4.625	-6.617 to -2.633	Yes	****	<0.0001
SCD vs. UV1-DS	-4.463	-6.455 to -2.470	Yes	****	<0.0001
SCD vs. Healthy	-6.063	-8.055 to -4.070	Yes	****	<0.0001
UV1 vs. SS	-1.338	-3.330 to 0.6548	No	ns	0.3898
UV1 vs. UV1-SS	-1.813	-3.805 to 0.1798	No	ns	0.0968
UV1 vs. DS	-3.338	-5.330 to -1.345	Yes	****	<0.0001
UV1 vs. UV1-DS	-3.175	-5.167 to -1.183	Yes	***	0.0002
UV1 vs. Healthy	-4.775	-6.767 to -2.783	Yes	****	<0.0001
SS vs. UV1-SS	-0.4750	-2.467 to 1.517	No	ns	0.9898
SS vs. DS	-2.000	-3.992 to -0.007737	Yes	*	0.0485
SS vs. UV1-DS	-1.838	-3.830 to 0.1548	No	ns	0.0886
SS vs. Healthy	-3.438	-5.430 to -1.445	Yes	****	<0.0001
UV1-SS vs. DS	-1.525	-3.517 to 0.4673	No	ns	0.2406
UV1-SS vs. UV1-DS	-1.363	-3.355 to 0.6298	No	ns	0.3675
UV1-SS vs. Healthy	-2.963	-4.955 to -0.9702	Yes	***	0.0006
DS vs. UV1-DS	0.1625	-1.830 to 2.155	No	ns	>0.9999
DS vs. Healthy	-1.438	-3.430 to 0.5548	No	ns	0.3050
UV1-DS vs. Healthy	-1.600	-3.592 to 0.3923	No	ns	0.1934

Table S8: Tukey's multiple comparisons test of hematocrit in BERK SCD mouse model (Figure 4C).

Tukey's multiple comparisons test	Mean Diff.	95.00% CI of diff.	Below threshold?	Summary	Adjusted P Value
SCD vs. UV1	-6.050	-12.75 to 0.6488	No	ns	0.1013
SCD vs. SS	-8.550	-15.25 to -1.851	Yes	**	0.0048
SCD vs. UV1-SS	-10.06	-16.76 to -3.364	Yes	***	0.0005
SCD vs. DS	-14.94	-21.64 to -8.239	Yes	****	<0.0001
SCD vs. UV1-DS	-16.01	-22.71 to -9.314	Yes	****	<0.0001
SCD vs. Healthy	-21.74	-28.44 to -15.04	Yes	****	<0.0001
UV1 vs. SS	-2.500	-9.199 to 4.199	No	ns	0.9099
UV1 vs. UV1-SS	-4.013	-10.71 to 2.686	No	ns	0.5277
UV1 vs. DS	-8.888	-15.59 to -2.189	Yes	**	0.0030
UV1 vs. UV1-DS	-9.963	-16.66 to -3.264	Yes	***	0.0006
UV1 vs. Healthy	-15.69	-22.39 to -8.989	Yes	****	<0.0001
SS vs. UV1-SS	-1.513	-8.211 to 5.186	No	ns	0.9923
SS vs. DS	-6.388	-13.09 to 0.3113	No	ns	0.0708
SS vs. UV1-DS	-7.463	-14.16 to -0.7637	Yes	*	0.0200
SS vs. Healthy	-13.19	-19.89 to -6.489	Yes	****	<0.0001
UV1-SS vs. DS	-4.875	-11.57 to 1.824	No	ns	0.2953
UV1-SS vs. UV1-DS	-5.950	-12.65 to 0.7488	No	ns	0.1122
UV1-SS vs. Healthy	-11.68	-18.37 to -4.976	Yes	****	<0.0001
DS vs. UV1-DS	-1.075	-7.774 to 5.624	No	ns	0.9988
DS vs. Healthy	-6.800	-13.50 to -0.1012	Yes	*	0.0445
UV1-DS vs. Healthy	-5.725	-12.42 to 0.9738	No	ns	0.1402

Table S9: Tukey's multiple comparisons test of reticulocytes in BERK SCD mouse model (Figure 4D).

Tukey's multiple comparisons test	Mean Diff.	95.00% CI of diff.	Below threshold?	Summary	Adjusted P Value
SCD vs. UV1	25.28	20.29 to 30.26	Yes	****	<0.0001
SCD vs. SS	28.34	23.36 to 33.32	Yes	****	<0.0001
SCD vs. UV1-SS	31.18	26.20 to 36.16	Yes	****	<0.0001
SCD vs. DS	33.73	28.75 to 38.71	Yes	****	<0.0001
SCD vs. UV1-DS	34.89	29.91 to 39.87	Yes	****	<0.0001
SCD vs. Healthy	39.15	34.17 to 44.13	Yes	****	<0.0001
UV1 vs. SS	3.063	-1.920 to 8.045	No	ns	0.4967
UV1 vs. UV1-SS	5.906	0.9240 to 10.89	Yes	*	0.0108
UV1 vs. DS	8.454	3.471 to 13.44	Yes	****	<0.0001
UV1 vs. UV1-DS	9.615	4.633 to 14.60	Yes	****	<0.0001
UV1 vs. Healthy	13.88	8.895 to 18.86	Yes	****	<0.0001
SS vs. UV1-SS	2.844	-2.139 to 7.826	No	ns	0.5839
SS vs. DS	5.391	0.4090 to 10.37	Yes	*	0.0261
SS vs. UV1-DS	6.553	1.570 to 11.53	Yes	**	0.0033
SS vs. Healthy	10.82	5.833 to 15.80	Yes	****	<0.0001
UV1-SS vs. DS	2.548	-2.435 to 7.530	No	ns	0.7004
UV1-SS vs. UV1-DS	3.709	-1.274 to 8.691	No	ns	0.2703
UV1-SS vs. Healthy	7.971	2.989 to 12.95	Yes	***	0.0002
DS vs. UV1-DS	1.161	-3.821 to 6.144	No	ns	0.9909
DS vs. Healthy	5.424	0.4415 to 10.41	Yes	*	0.0247
UV1-DS vs. Healthy	4.263	-0.7198 to 9.245	No	ns	0.1394

Table S10: Tukey's multiple comparisons test of sickled cells in BERK SCD mouse model (Figure 4E).

Tukey's multiple comparisons test	Mean Diff.	95.00% CI of diff.	Below threshold?	Summary	Adjusted P Value
SCD vs. UV1	31.67	25.67 to 37.67	Yes	****	<0.0001
SCD vs. SS	34.13	28.13 to 40.13	Yes	****	<0.0001
SCD vs. UV1-SS	35.67	29.67 to 41.67	Yes	****	<0.0001
SCD vs. DS	38.91	32.91 to 44.91	Yes	****	<0.0001
SCD vs. UV1-DS	39.44	33.44 to 45.44	Yes	****	<0.0001
SCD vs. Healthy	45.14	39.14 to 51.14	Yes	****	<0.0001
UV1 vs. SS	2.459	-3.541 to 8.459	No	ns	0.8664
UV1 vs. UV1-SS	3.996	-2.004 to 9.995	No	ns	0.3995
UV1 vs. DS	7.242	1.242 to 13.24	Yes	**	0.0089
UV1 vs. UV1-DS	7.771	1.771 to 13.77	Yes	**	0.0040
UV1 vs. Healthy	13.47	7.466 to 19.47	Yes	****	<0.0001
SS vs. UV1-SS	1.536	-4.464 to 7.536	No	ns	0.9852
SS vs. DS	4.782	-1.218 to 10.78	No	ns	0.2004
SS vs. UV1-DS	5.312	-0.6879 to 11.31	No	ns	0.1144
SS vs. Healthy	11.01	5.007 to 17.01	Yes	****	<0.0001
UV1-SS vs. DS	3.246	-2.754 to 9.246	No	ns	0.6430
UV1-SS vs. UV1-DS	3.776	-2.224 to 9.776	No	ns	0.4683
UV1-SS vs. Healthy	9.471	3.471 to 15.47	Yes	***	0.0002
DS vs. UV1-DS	0.5296	-5.470 to 6.530	No	ns	>0.9999
DS vs. Healthy	6.225	0.2249 to 12.22	Yes	*	0.0373
UV1-DS vs. Healthy	5.695	-0.3048 to 11.69	No	ns	0.0731

Table S11: Tukey's multiple comparisons test of erythroid precursor cells in BERK SCD mouse model (Figure 4F).

Tukey's multiple comparisons test	Mean Diff.	95.00% CI of diff.	Below threshold?	Summary	Adjusted P Value
SCD vs. UV1	28.70	23.00 to 34.40	Yes	****	<0.0001
SCD vs. SS	31.85	26.15 to 37.55	Yes	****	<0.0001
SCD vs. UV1-SS	32.87	27.17 to 38.57	Yes	****	<0.0001
SCD vs. DS	35.97	30.27 to 41.67	Yes	****	<0.0001
SCD vs. UV1-DS	36.67	30.97 to 42.37	Yes	****	<0.0001
SCD vs. Healthy	40.50	34.80 to 46.20	Yes	****	<0.0001
UV1 vs. SS	3.154	-2.546 to 8.854	No	ns	0.6188
UV1 vs. UV1-SS	4.171	-1.529 to 9.871	No	ns	0.2891
UV1 vs. DS	7.273	1.572 to 12.97	Yes	**	0.0048
UV1 vs. UV1-DS	7.969	2.269 to 13.67	Yes	**	0.0015
UV1 vs. Healthy	11.80	6.101 to 17.50	Yes	****	<0.0001
SS vs. UV1-SS	1.018	-4.683 to 6.718	No	ns	0.9979
SS vs. DS	4.119	-1.581 to 9.819	No	ns	0.3034
SS vs. UV1-DS	4.815	-0.8851 to 10.52	No	ns	0.1494
SS vs. Healthy	8.648	2.947 to 14.35	Yes	***	0.0005
UV1-SS vs. DS	3.101	-2.599 to 8.801	No	ns	0.6370
UV1-SS vs. UV1-DS	3.798	-1.903 to 9.498	No	ns	0.3990
UV1-SS vs. Healthy	7.630	1.930 to 13.33	Yes	**	0.0027
DS vs. UV1-DS	0.6962	-5.004 to 6.396	No	ns	0.9998
DS vs. Healthy	4.529	-1.171 to 10.23	No	ns	0.2035
UV1-DS vs. Healthy	3.833	-1.868 to 9.533	No	ns	0.3880

Table S12: Tukey’s multiple comparisons test of VCN in BERK SCD mouse model (Figure 5A).

Tukey's multiple comparisons test	Mean Diff.	95.00% CI of diff.	Below threshold?	Summary	Adjusted P Value
UV1 vs. SS	3.099	-1.859 to 8.056	No	ns	0.3914
UV1 vs. UV1-SS	-7.673	-12.63 to -2.715	Yes	***	0.0008
UV1 vs. DS	-3.921	-8.879 to 1.036	No	ns	0.1775
UV1 vs. UV1-DS	-14.60	-19.55 to -9.637	Yes	****	<0.0001
SS vs. UV1-SS	-10.77	-15.73 to -5.814	Yes	****	<0.0001
SS vs. DS	-7.020	-11.98 to -2.062	Yes	**	0.0022
SS vs. UV1-DS	-17.69	-22.65 to -12.74	Yes	****	<0.0001
UV1-SS vs. DS	3.751	-1.206 to 8.709	No	ns	0.2127
UV1-SS vs. UV1-DS	-6.923	-11.88 to -1.965	Yes	**	0.0026
DS vs. UV1-DS	-10.67	-15.63 to -5.716	Yes	****	<0.0001

Table S13: Tukey's multiple comparisons test of anti-sickling globin induction normalized to VCN in BERK SCD mouse model (Figure 5C).

Tukey's multiple comparisons test	Mean Diff.	95.00% CI of diff.	Below threshold?	Summary	Adjusted P Value
UV1 vs. SS	3.099	-1.859 to 8.056	No	ns	0.3914
UV1 vs. UV1-SS	-7.673	-12.63 to -2.715	Yes	***	0.0008
UV1 vs. DS	-3.921	-8.879 to 1.036	No	ns	0.1775
UV1 vs. UV1-DS	-14.60	-19.55 to -9.637	Yes	****	<0.0001
SS vs. UV1-SS	-10.77	-15.73 to -5.814	Yes	****	<0.0001
SS vs. DS	-7.020	-11.98 to -2.062	Yes	**	0.0022
SS vs. UV1-DS	-17.69	-22.65 to -12.74	Yes	****	<0.0001
UV1-SS vs. DS	3.751	-1.206 to 8.709	No	ns	0.2127
UV1-SS vs. UV1-DS	-6.923	-11.88 to -1.965	Yes	**	0.0026
DS vs. UV1-DS	-10.67	-15.63 to -5.716	Yes	****	<0.0001

Table S14: Tukey's multiple comparisons test of spleen mass in BERK SCD mouse model (Figure 5C).

Tukey's multiple comparisons test	Mean Diff.	95.00% CI of diff.	Below threshold?	Summary	Adjusted P Value
SCD vs. UV1	0.2325	0.1495 to 0.3155	Yes	****	<0.0001
SCD vs. SS	0.2938	0.2108 to 0.3767	Yes	****	<0.0001
SCD vs. UV1-SS	0.3275	0.2445 to 0.4105	Yes	****	<0.0001
SCD vs. DS	0.3663	0.2833 to 0.4492	Yes	****	<0.0001
SCD vs. UV1-DS	0.3850	0.3020 to 0.4680	Yes	****	<0.0001
SCD vs. Healthy	0.4625	0.3795 to 0.5455	Yes	****	<0.0001
UV1 vs. SS	0.06125	-0.02172 to 0.1442	No	ns	0.2794
UV1 vs. UV1-SS	0.09500	0.01203 to 0.1780	Yes	*	0.0154
UV1 vs. DS	0.1338	0.05078 to 0.2167	Yes	***	0.0002
UV1 vs. UV1-DS	0.1525	0.06953 to 0.2355	Yes	****	<0.0001
UV1 vs. Healthy	0.2300	0.1470 to 0.3130	Yes	****	<0.0001
SS vs. UV1-SS	0.03375	-0.04922 to 0.1167	No	ns	0.8705
SS vs. DS	0.07250	-0.01047 to 0.1555	No	ns	0.1236
SS vs. UV1-DS	0.09125	0.008283 to 0.1742	Yes	*	0.0225
SS vs. Healthy	0.1688	0.08578 to 0.2517	Yes	****	<0.0001
UV1-SS vs. DS	0.03875	-0.04422 to 0.1217	No	ns	0.7799
UV1-SS vs. UV1-DS	0.05750	-0.02547 to 0.1405	No	ns	0.3518
UV1-SS vs. Healthy	0.1350	0.05203 to 0.2180	Yes	***	0.0001
DS vs. UV1-DS	0.01875	-0.06422 to 0.1017	No	ns	0.9923
DS vs. Healthy	0.09625	0.01328 to 0.1792	Yes	*	0.0135
UV1-DS vs. Healthy	0.07750	-0.005467 to 0.1605	No	ns	0.0815

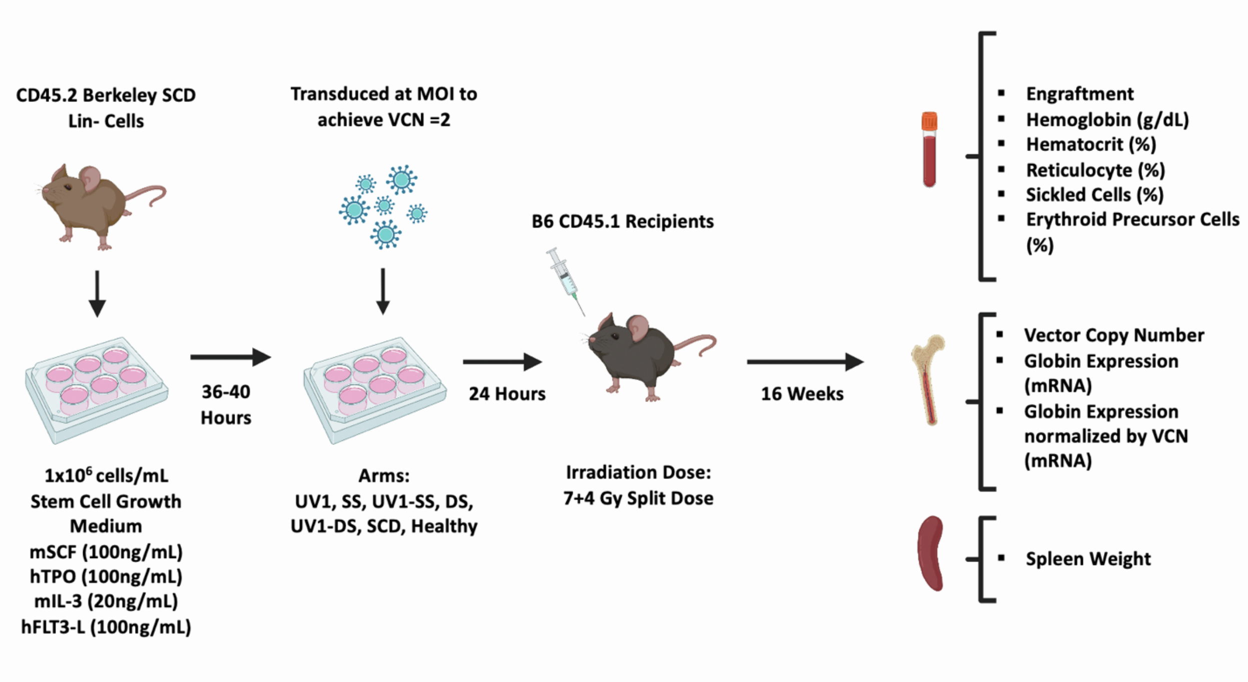

Figure S1. Schematic of experimental plan for the *in vivo* Berkeley SCD mouse model. Image created with BioRender.

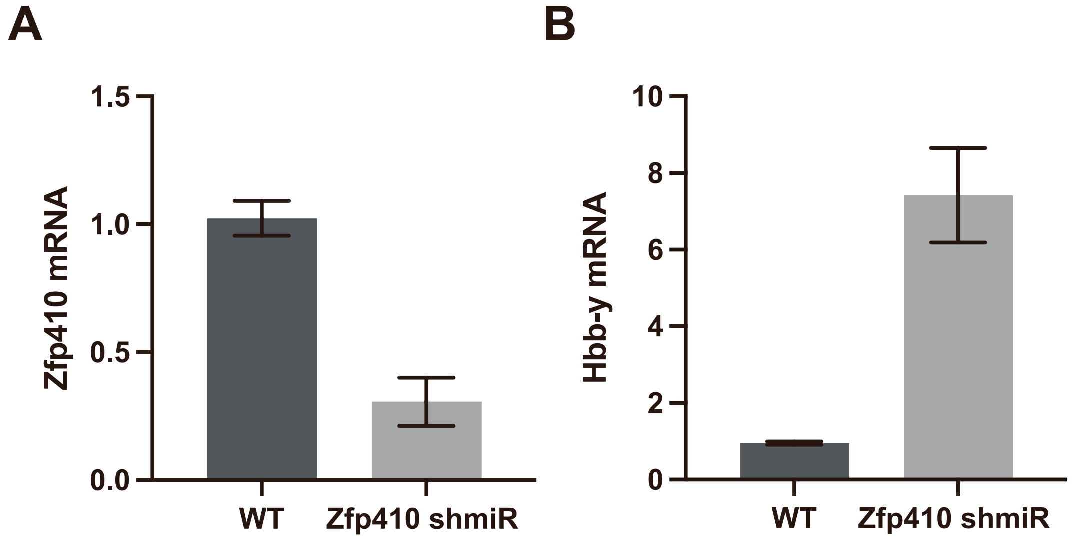

Figure S2. Efficient knockdown of Zfp410 by Zfp410 shmiR vector leads to high Hbb-y induction in erythroid differentiated MEL cells *in vitro*. Zfp410 and Hbb-y mRNA expression as measured by RT–qPCR with Gapdh as control. Data represent mean ± SD.

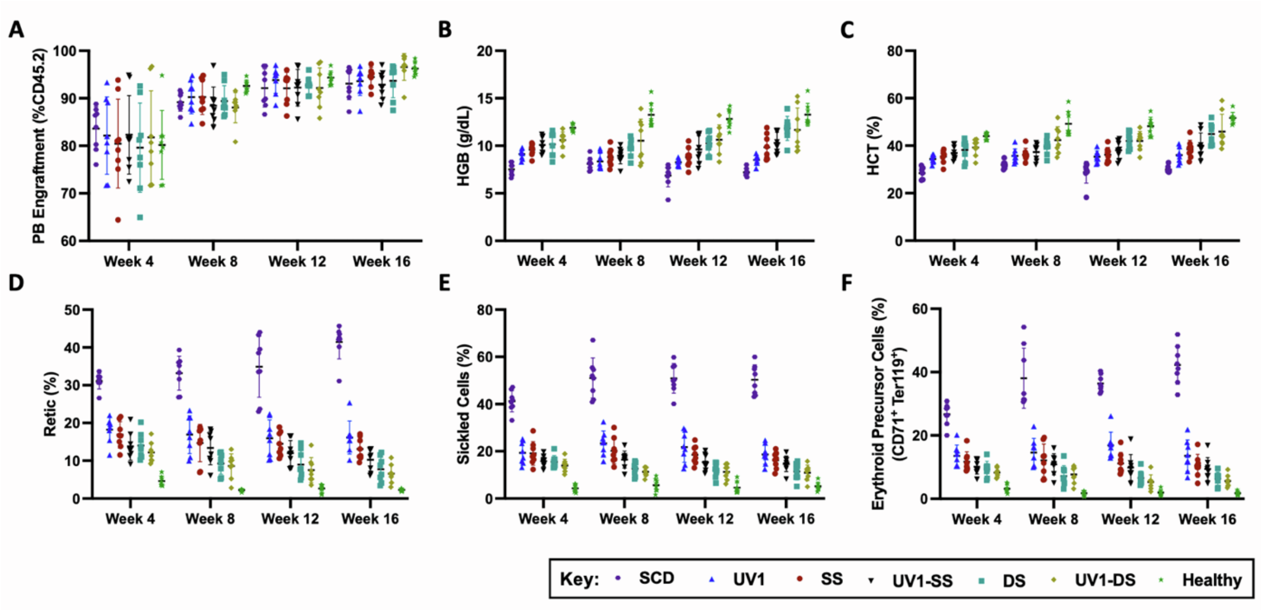

Figure S3. Correction of peripheral blood sickle cell hematologic parameters *in vivo* in Berkeley SCD mouse model (Weeks 4, 8, 12, 16). Lineage-negative (lin-) bone marrow cells from BERK mice (CD45.2) were transduced with each vector or mock-transduced as control and transplanted into irradiated CD45.1+ BL/6 mouse recipients. Mice were bled at 4, 8, 12, and 16 weeks after transplant and peripheral blood (PB) was analyzed. (A) Engraftment was assessed in PB by flow cytometry (%CD45.2+ cells). (B) Hemoglobin (g/dL) , (C) hematocrit and (D) reticulocyte counts (%) are shown. (E) PB was treated with sodium metabisulfite for 30 min to induce sickling. Percentage of sickled RBCs from PB sample was quantified. (F) Percentages of CD71+ Ter119+ high erythroid precursor cell population in PB. Error bars represent mean ± SD. Symbols indicate mice transplanted with different shmiR vectors or non-transduced cells (SCD); each data point represents an individual mouse, N=8

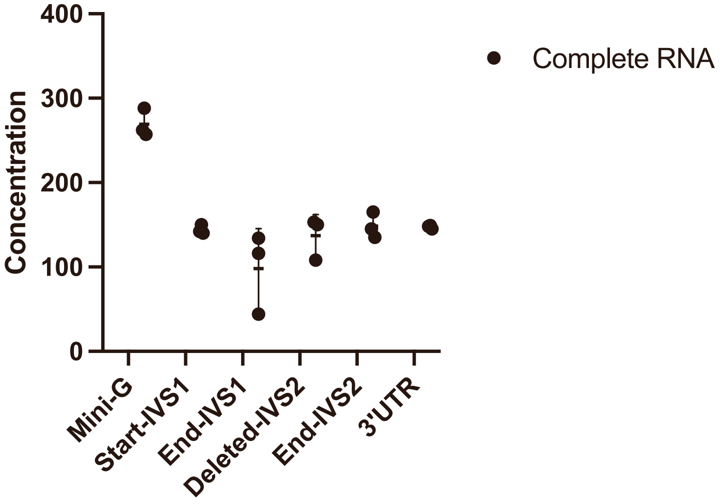

Figure S4: Assessment of complete viral RNA from unconcentrated viral supernatant. Vectors were packaged with an HEK293T *PKR* knock-out cell line. Unconcentrated viral supernatants were collected, and viral RNA was extracted and quantified with ddPCR. Each point on the plot represents a vector packaged from an individual 10cm plate. Error bars represent mean ± SD.

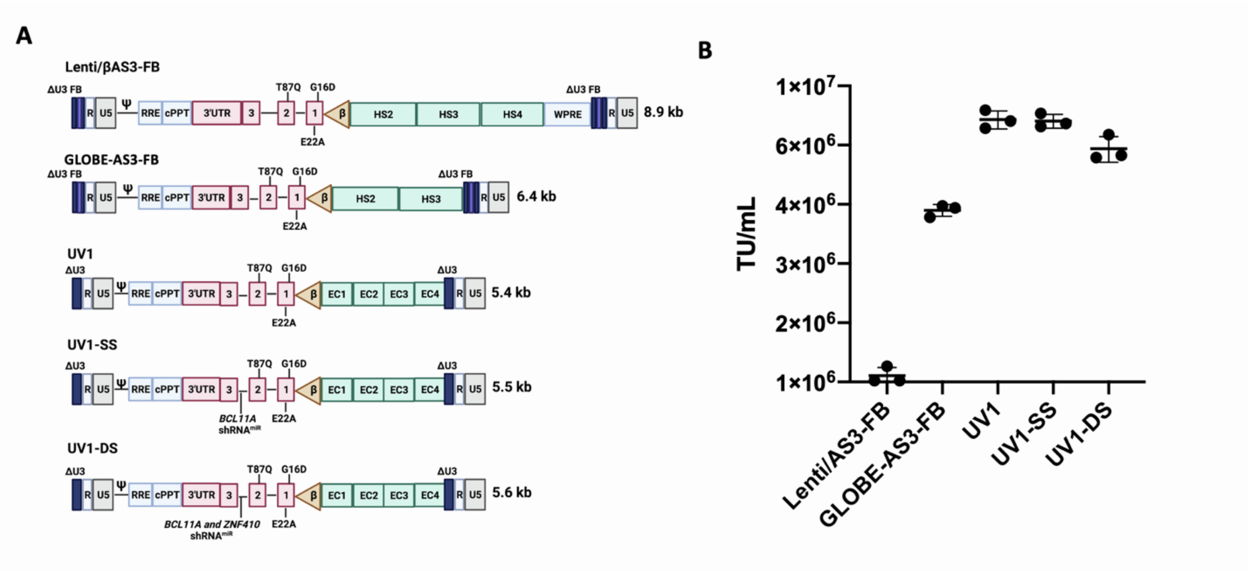

Figure S5: UV1-shmiR vectors maintain high titers compared to other β^{AS3}-globin LVs. (A) Schematics of β^{AS3}-globin LVs: Lenti/β^{AS3}-FB[2] (8.9kb), GLOBE-AS3-FB[4] (6.4kb), UV1[5] (5.4kb), UV1-SS (5.5kb), UV1-DS (5.6kb). Vectors were packaged with an HEK293T *PKR* knock-out cell line and titers were determined by HT-29 cell line transduction, using unconcentrated viral supernatant, and quantified with ddPCR. Each point on the plot represents vector packaged and titered from an individual 10cm plate. Error bars represent mean ± SD. Image created with BioRender.

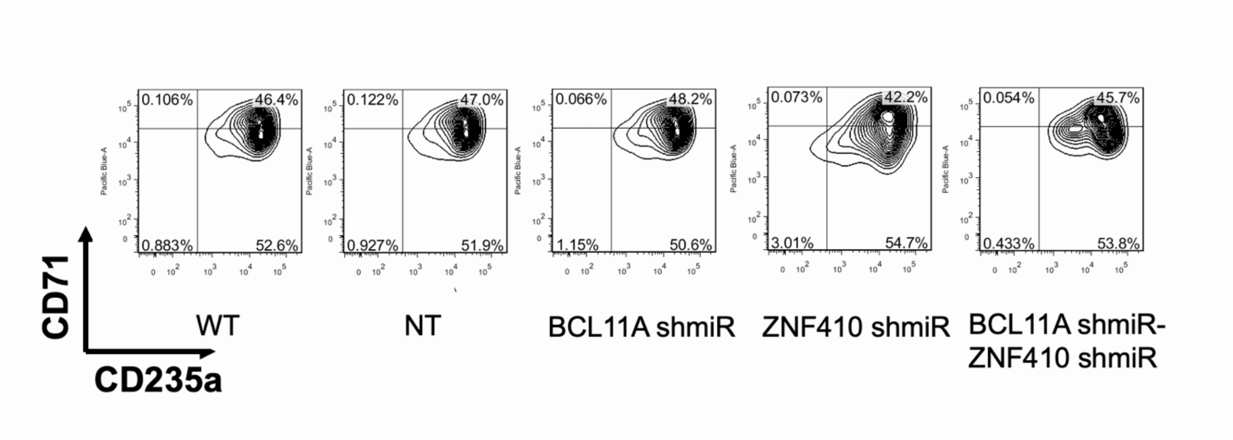

Figure S6. Differentiation status of erythroid cells after 18 days in culture using CD71 and CD235a, WT is wild type cells, NT is CD34+ cells transduced with lentivirus contain non-target shmiR, BCL11A shmiR is CD34+ cells transduced with lentivirus contain BCL11A shmiR, ZNF410 shmiR is CD34+ cells transduced with lentivirus contain ZNF410 shmiR, BCL11A shmiR-ZNF410 shmiR is CD34+ cells transduced with lentivirus contain BCL11A shmiR and ZNF410 shmiR.

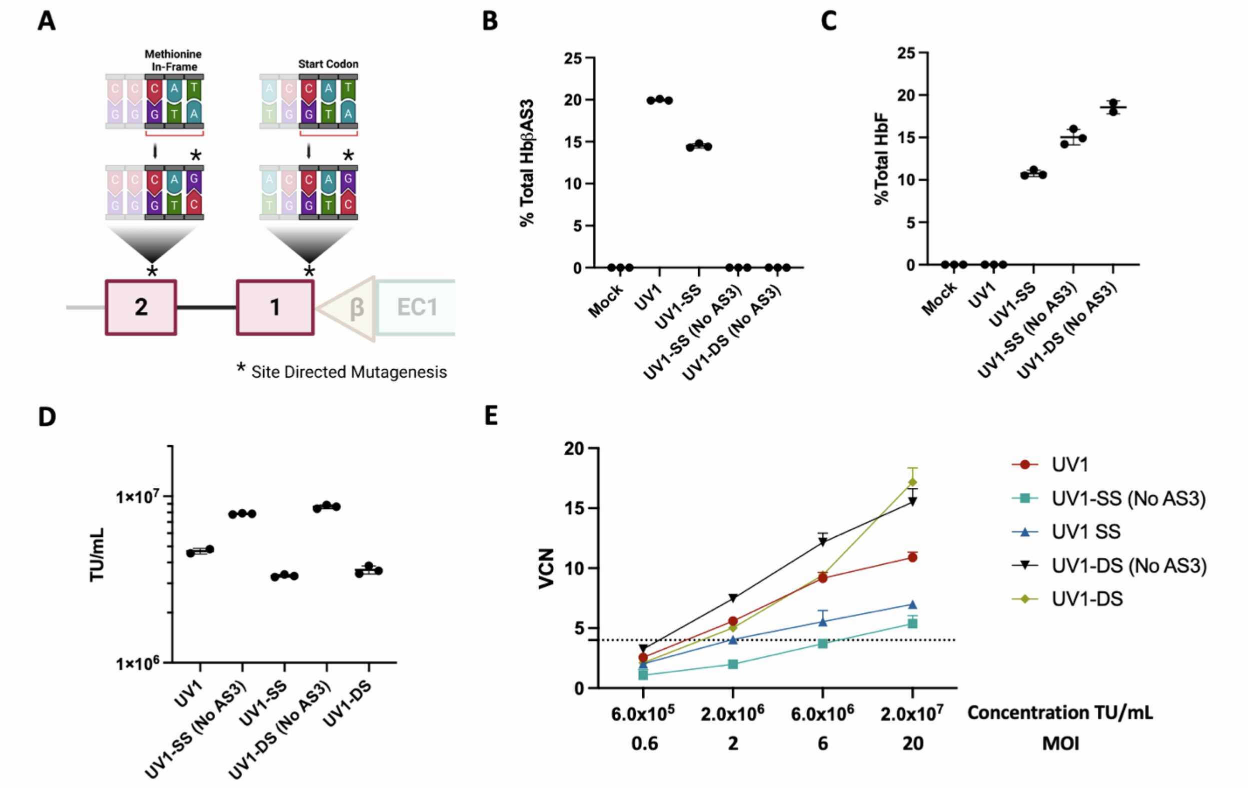

Figure S7. Fetal globin positive control vector design, titer, and gene transfer. (A) Site-directed mutagenesis strategy to change methionine codons (ATG) in the β^{AS3}-globin open reading frame (B) HUDEP-2 parental cells were transduced with vectors at $3.0x10^5$ TU/mL and then subjected to erythroid differentiation *in vitro* for 12 days and protein was assessed by HPLC. Induction of HbB^{AS3} (C) Induction of HbF (D) Vectors were packaged with an HEK293T *PKR* knock-out cell line and titers were determined by HT-29 cell line transduction, using raw viral supernatant, and quantified with ddPCR. Each point on the plot represents vector packaged and titered from an individual 10cm plate. (E) Lin- cells from Townes mice were transduced with constructs at $6x10^5$ TU/mL, $2x10^6$ TU/mL, $6x10^6$ TU/mL and $2x10^7$ TU/mL (MOI: 0.6, 2, 6, and 20) and cultured for 14 days under myeloid differentiation conditions to assess levels of infectivity. Vector copy number (VCN) was measured by ddPCR. Error bars represent mean ± SD. Image created with BioRender.

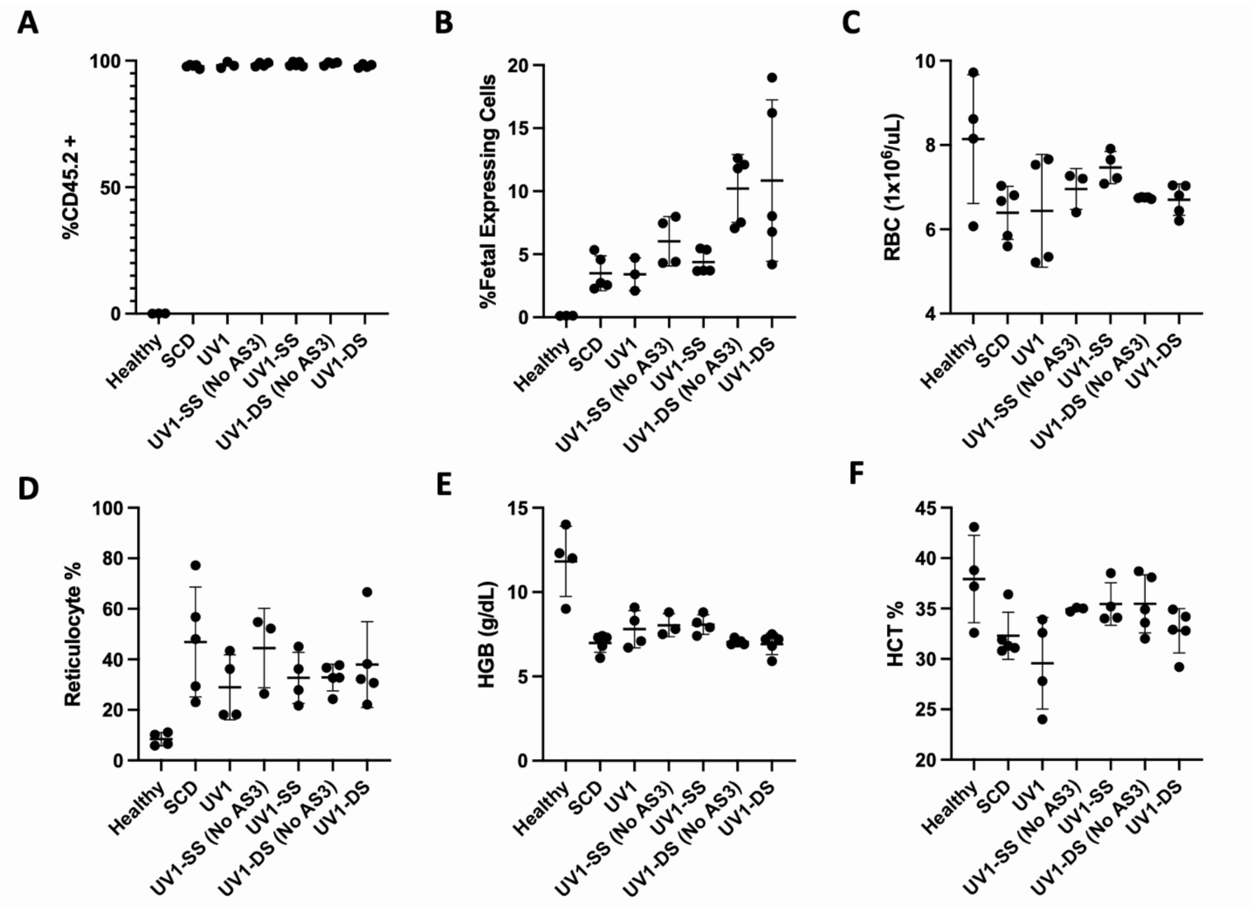

Figure S8. Peripheral blood sickle cell disease erythroid cell parameters *in vivo* in Townes SCD mouse model. Lineage negative (lin-) bone marrow cells from Townes mice (CD45.2) were transduced with each vector or mock-transduced as control and transplanted into irradiated B6 CD45.1+ (Pep Boy) mouse recipients. Mice were bled at 16 weeks after transplant and PB was analyzed. (A) Engraftment was assessed in PB by flow cytometry (%CD45.2+ cells). (B) Percentage of fetal globin expressing cells was assessed by intracellular staining and flow cytometry. (C) Red blood cell counts ($1x10^6$/uL) (D) Reticulocyte counts (%). (E) Hemoglobin (g/dL). (F) Hematocrit. Error bars represent mean ± SD. Each data point represents an individual mouse.

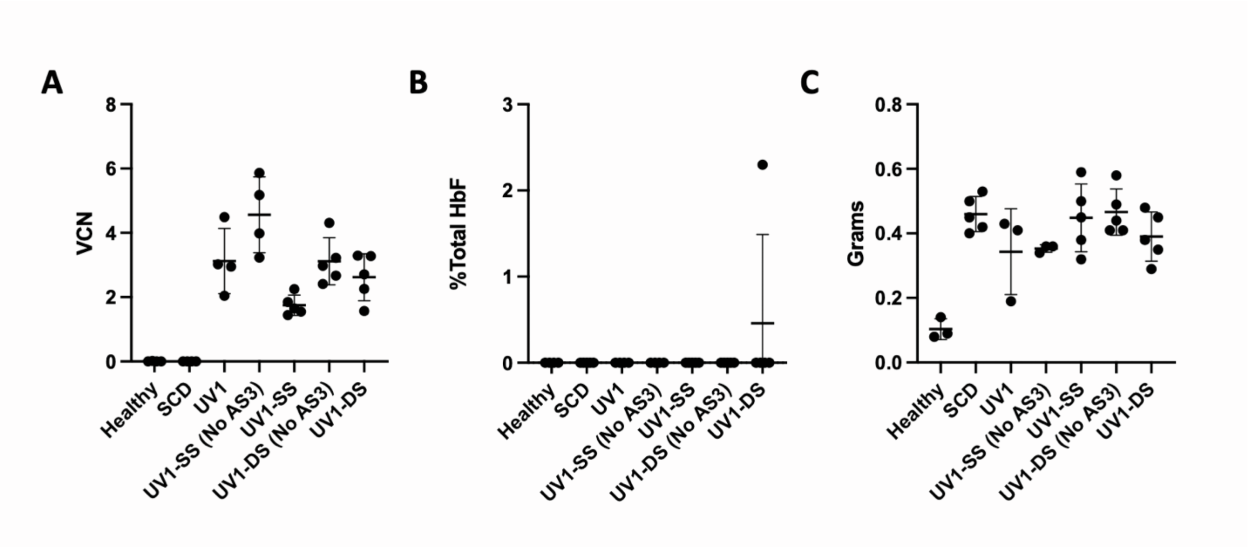

Figure S9. Vector Copy Number, HbF Expression by HPLC, and Spleen Weight

Mice were euthanized at 16 weeks after transplant and whole bone marrow (BM) and spleen was harvested and analyzed individually. (A) VCN in BM was determined by ddPCR. (B) Percentages of HbF expression was determined by HPLC. (C) Spleen weights. Error bars represent mean ± SD. Each data point represents an individual mouse.

References

1. Ryan, T.M., Ciavatta, D.J., and Townes, T.M. (1997). Knockout-Transgenic Mouse Model of Sickle Cell Disease. Science *278*, 873–876. 10.1126/science.278.5339.873.

2. Levasseur, D.N., Ryan, T.M., Pawlik, K.M., and Townes, T.M. (2003). Correction of a mouse model of sickle cell disease: lentiviral/antisickling beta-globin gene transduction of unmobilized, purified hematopoietic stem cells. Blood *102*, 4312–4319. 10.1182/blood-2003-04-1251.

3. Woodard, K.J., Doerfler, P.A., Mayberry, K.D., Sharma, A., Levine, R., Yen, J., Valentine, V., Palmer, L.E., Valentine, M., and Weiss, M.J. (2022). Limitations of mouse models for sickle cell disease conferred by their human globin transgene configurations. Disease Models & Mechanisms *15*, dmm049463. 10.1242/dmm.049463.

4. Poletti, V., Urbinati, F., Charrier, S., Corre, G., Hollis, R.P., Campo Fernandez, B., Martin, S., Rothe, M., Schambach, A., Kohn, D.B., et al. (2018). Pre-clinical Development of a Lentiviral Vector Expressing the Anti-sickling βAS3 Globin for Gene Therapy for Sickle Cell Disease. Mol Ther Methods Clin Dev *11*, 167–179. 10.1016/j.omtm.2018.10.014.

5. Morgan, R.A., Unti, M.J., Aleshe, B., Brown, D., Osborne, K.S., Koziol, C., Ayoub, P.G., Smith, O.B., O'Brien, R., Tam, C., et al. (2020). Improved Titer and Gene Transfer by Lentiviral Vectors Using Novel, Small β-Globin Locus Control Region Elements. Molecular Therapy *28*, 328–340. 10.1016/j.ymthe.2019.09.020.
