# Supplementary material for: A novel high-titer, bifunctional lentiviral vector for autologous hematopoietic stem cell gene therapy of sickle cell disease
Source: Mol Ther Methods Clin Dev. 2024 Apr 24;32(2):101254. doi: 10.1016/j.omtm.2024.101254 (PMC11091523; doi:10.1016/j.omtm.2024.101254)
Supplement: Document S2. Article plus supplemental information [file mmc2.pdf]

Molecular Therapy
Methods & Clinical Development
Original Article

A novel high-titer, bifunctional lentiviral vector for autologous hematopoietic stem cell gene therapy of sickle cell disease

Kevyn L. Hart,[1,11] Boya Liu,[2,5,11] Devin Brown,[3] Beatriz Campo-Fernandez,[3] Kevin Tam,[3] Katherine Orr,[4] Roger P. Hollis,[3] Christian Brendel,[2,5,6,7] David A. Williams,[2,5,6,7] and Donald B. Kohn[3,8,9,10]

[1]Department of Human Genetics, David Geffen School of Medicine, University of California, Los Angeles, Los Angeles, CA 90095, USA; [2]Division of Hematology/Oncology, Boston Children's Hospital, Boston, MA 02115, USA; [3]Department of Microbiology, Immunology & Molecular Genetics, David Geffen School of Medicine at University of California, Los Angeles, Los Angeles, CA 90095, USA; [4]CSUN-UCLA Stem Cell Scientist Training Program, California State University, Northridge, Northridge, CA 91330, USA; [5]Department of Pediatrics, Harvard Medical School, Boston, MA 02115, USA; [6]Department of Pediatric Oncology, Dana-Farber Cancer Institute, Boston, MA 02215, USA; [7]Harvard Stem Cell Institute, Harvard University, Boston, MA 02138, USA; [8]Department of Molecular and Medical Pharmacology, David Geffen School of Medicine at University of California, Los Angeles, Los Angeles, CA 90095, USA; [9]Department of Pediatrics, David Geffen School of Medicine at University of California, Los Angeles, Los Angeles, CA 90095, USA; [10]The Eli & Edythe Broad Center of Regenerative Medicine & Stem Cell Research, University of California, Los Angeles, Los Angeles, CA 90095, USA

A major limitation of gene therapy for sickle cell disease (SCD) is the availability and access to a potentially curative one-time treatment, due to high treatment costs. We have developed a high-titer bifunctional lentiviral vector (LVV) in a vector backbone that has reduced size, high vector yields, and efficient gene transfer to human CD34$^+$ hematopoietic stem and progenitor cells (HSPCs). This LVV contains locus control region cores expressing an anti-sickling β^{AS3}-globin gene and two microRNA-adapted short hairpin RNA simultaneously targeting *BCL11A* and *ZNF410* transcripts to maximally induce fetal hemoglobin (HbF) expression. This LVV induces high levels of anti-sickling hemoglobins (HbA^{AS3} + HbF), while concurrently decreasing sickle hemoglobin (HbS). The decrease in HbS and increased anti-sickling hemoglobin impedes deoxygenated HbS polymerization and red blood cell sickling at low vector copy per cell in transduced SCD patient CD34$^+$ cells differentiated into erythrocytes. The dual alterations in red cell hemoglobins ameliorated the SCD phenotype in the SCD Berkeley mouse model *in vivo*. With high titer and enhanced transduction of HSPC at a low multiplicity of infection, this LVV will increase the number of patient doses of vector from production lots to decrease costs and help improve accessibility to gene therapy for SCD.

INTRODUCTION

Sickle cell disease (SCD) is a condition characterized by the production of abnormal hemoglobin (HGB) (sickle HGB [HbS]), caused by a specific genetic mutation in the β-globin gene. This mutation results in the substitution of glutamine for valine at position 6 (E6V) and triggers the polymerization HbS upon deoxygenation. SCD is associated with multiple complications including chronic hemolytic anemia, severe pain episodes (vaso-occlusive events [VOEs]), strokes, and organ damage.[1] SCD affects approximately 300,000–400,000 newborns annually and around 20 million people worldwide, with an estimated 100,000 individuals affected in the United States.[2] Current treatments include blood transfusions and medications aimed at reducing VOEs and hemolysis, which include hydroxyurea,[3] L-glutamine,[4] crizanlizumab,[5] and voxelotor,[6] but these options are non-curative. The only standard of care curative approach is allogeneic hematopoietic stem cell transplantation (HSCT) with a suitably-matched donor. Adverse side effects of allogeneic HSCT include acute and chronic conditioning toxicities, graft failure, and graft-vs-host disease. In addition, many patients lack appropriate donors. In recent years, autologous HSCT/gene therapy for SCD has moved from an attractive concept to clinical reality, with a variety of approaches currently in clinical testing appearing to provide sustained clinical benefits to SCD patients.

The development and manufacturing costs of gene therapy products are significant factors that may pose barriers to the accessibility of curative treatment for patients. The cost of allotransplantation or gene therapy treatment for SCD can vary depending on several factors including the type of therapy used and complications from the treatment, the location of the treatment center, and the cost of the cell product used. With the cost of recently approved gene therapy

Received 13 February 2024; accepted 18 April 2024;
https://doi.org/10.1016/j.omtm.2024.101254.

[11]These authors contributed equally

Correspondence: David A. Williams, Division of Hematology/Oncology, Boston Children's Hospital, Boston, MA 02115, USA.
E-mail: dawilliams@childrens.harvard.edu
Correspondence: Donald B. Kohn, Department of Microbiology, Immunology & Molecular Genetics, David Geffen School of Medicine at University of California, Los Angeles, Los Angeles, CA 90095, USA.
E-mail: dkohn1@mednet.ucla.edu

This is an open access article under the CC BY license (http://creativecommons.org/licenses/by/4.0/).

products being approximately $1–3 million per treatment including the manufacturing of the Medicinal Drug Product as well as the administration of therapy in the setting of a myeloablative transplant with associated medical costs, many individuals will face challenges in accessing approved therapies. To help address these accessibility issues and provide benefit to a larger patient population, it is crucial to develop strategies aimed at decreasing the production costs of complex autologous stem cell gene therapies. Research and development is critical to enhance vector production, increase vector yields, and develop more cost-effective approaches to scale up manufacturing.

One strategy to decrease costs of manufacturing includes engineering smaller lentiviral vectors (LVVs) and enhancing both the efficiency of hematopoietic stem and progenitor cell (HSPC) transduction at lower vector multiplicity of infection (MOI) and enhancing the biological effect of the payload at low MOI in the target cell population. LVVs in current clinical use for the treatment of β-hemoglobinopathies including SCD have relatively large genomes (e.g., 8–9 kb) that include essential enhancer elements from the β-globin locus control region (LCR), to obtain high-level erythroid-specific expression.[7] These vectors are costly to produce and are relatively inefficient in transducing human $CD34^+$ HSPC, in part due to high percentages of incomplete virion genomes, and may require a high MOI due to low expression/integrated vector genome.[8] They have required the use of transduction enhancer compounds to improve their infectivity at clinical scale. Development of refined β-globin LVV with reduced sizes of the LCR based on bioinformatics design has led to significantly improved titers and $CD34^+$ cell infectivity compared with current clinical vectors.[9]

Two successful approaches to genetic therapy for SCD include expressing a modified β-globin gene with anti-sickling characteristics (e.g., T87Q, βAS3), and inducing fetal HGB (HbF) by increasing expression of γ-globin. HbF has potent anti-sickling characteristics and induction via reversing the fetal-adult HGB switch has the additional benefit of concurrently and coordinately decreasing β^S-globin production. Intracellular polymerization of deoxygenated HGB is exquisitely sensitive to the concentration of HbS in the red cell.[10] Thus, a strategy to both increase the level of anti-sickling HGB and decrease the concentration of HbS may prove most efficient in decreasing HbS polymerization and thus cellular sickling phenotypes.

We developed an optimized anti-sickling β-globin LVV (UV1)[9] of minimal size using a bifunctional approach to treating SCD to help address the barriers to accessibility imposed by high vector costs. The first mechanism in this approach incorporates a modified β-globin gene (β^{AS3}-globin).[11] β^{AS3}-globin contains three amino acid substitutions (G16D, E22A, and T87Q) that give this β-globin variant anti-sickling properties similar to fetal γ-globin. These amino acid changes incorporated into the β^{AS3}-globin polypeptide decrease sickle polymerization through disruption of axial and lateral contact with the canonical valine 6 of sickle β-globin and also confer a competitive advantage over the sickle β-globin chain for binding to α-globin chains to form HGB tetramers. Vectors carrying the β^{AS3}-globin transgene corrected hematologic and clinical findings in the Townes Sickle Cell mouse model, and were also shown to transduce SCD patient BM $CD34^+$ cells and induce therapeutic levels of HbA^{AS3}–globin to correct red blood cell (RBC) physiology.[11,12]

The second approach incorporated in this vector utilizes microRNA-adapted short hairpin RNAs (shmiRs)[13] to simultaneously target *BCL11A* and *ZNF410*, two independent repressors of γ-globin expression, to induce HbF. HbF induction is a strategy in current clinical testing to ameliorate SCD phenotypes based on the observation that elevated levels of HbF attenuate clinical severity of SCD.[14–16] The prime example is co-inheritance of mutations causing hereditary persistence of HbF (HPFH) with SCD leads to marked attenuation of SCD phenotypes in comparison to individuals without HPFH.[17] BCL11A was identified as an important repressor of fetal globin expression based on genome-wide association study mapping.[18,19] Further studies showed that generating a *BCL11A* knockout in SCD mouse models corrected the pathogenic defects associated with SCD through increased HbF expression.[20] An LVV expressing a *BCL11A* shmiR (BCH-BB694) only in the erythroid lineage under the control of the β-globin promoter and regulatory elements derived from HS2 and HS3 of the LCR ameliorated the sickle phenotype in mice and induced up to 40% HbF induction in erythroid differentiated SCD $CD34^+$ cells.[21] A Phase I clinical trial with BCH-BB694 showed a sustained increase of HbF levels with a median of 30.5% of all HGB levels in six patients with significant mitigation of sickle phenotype at an average *in vivo* vector copy number (VCN) of approximately 1 copy per diploid genome.[22] In addition to BCL11A, ZNF410 has also been shown to be a repressor of γ-globin expression.[23,24] Combining *BCL11A* and *ZNF410* shmiRs has been shown to increase HbF induction by approximately an additional 10% compared with knockdown of *BCL11A* alone, with enhanced anti-sickling results in SCD erythroid differentiated $CD34^+$ cells.[13] Brusson et al.[25] reported a bifunctional LVV for SCD that expressed the same β^{AS3}-globin gene described here combined with an artificial miR to HbS. They observed a higher level of correction of parameters of SCD by this bifunctional vector compared with one expressing only the β^{AS3}-globin gene.

Here we have combined both approaches in a small, highly efficient vector for treating SCD. The bifunctional vector described here (UV1-DS) maintains high titers of production and high $CD34^+$ cell transduction activity at reduced MOI, which may provide a lower cost of manufacturing for the LVV component of autologous hematopoietic stem cell gene therapy. We demonstrate that differentiation of hematopoietic stem/progenitor cells (HSPCs) transduced with the UV1-DS vector leads to reversal of the sickle cellular phenotype with cellular parameters equivalent to normal red blood cells.

RESULTS

Design and assessment of the UV1-DS vector

Previous studies have shown success in ameliorating the sickle cell phenotype with both *BCL11A* shmiR and β^{AS3}-globin technologies.

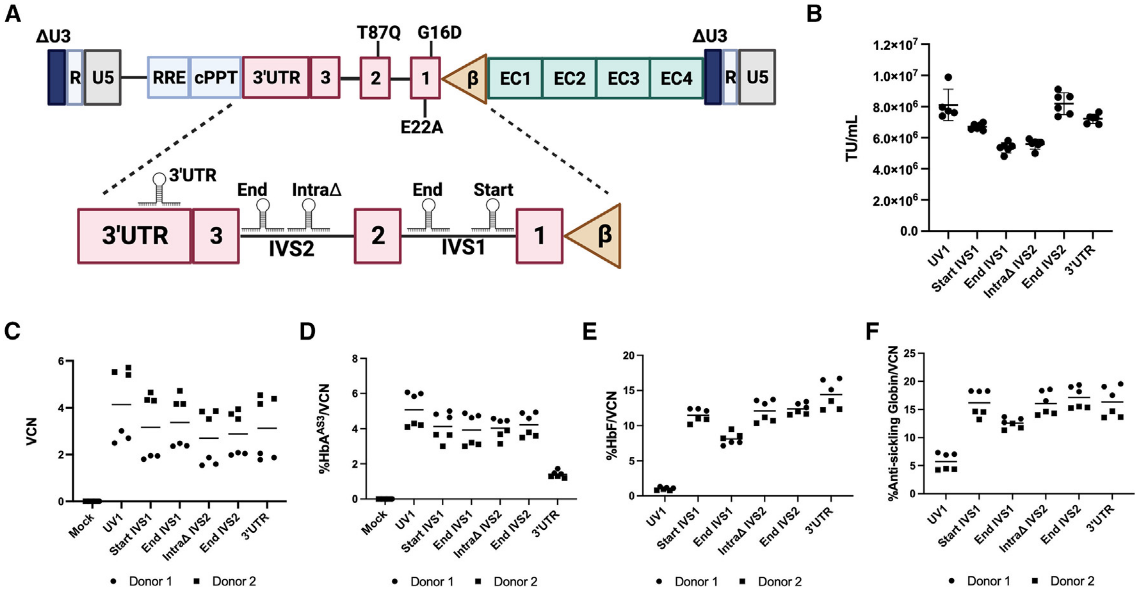

Figure 1. Assessment of the optimal location to incorporate the *BCL11A* shmiR into the UV1 vector

(A) Schematic of UV1-shmiR LVV series and cloning design. The UV1 LVV containing a β^{AS3}-globin cassette in reverse orientation expressed under the control of the β-globin promoter and Encode core (EC) enhancer regions derived and minimized from hypersensitive sites 1, 2, 3, and 4 (HS1, HS2, HS3, and HS4) of the β-globin LCR.[9] Insertion DNA sequence sites in Table S1. (B) Unconcentrated viral titers of vectors in which the *BCL11A* shmiR[27] was introduced into 5 locations including: two sites in intron 1 (Start, End), two sites in IVS2 (IntraΔ, End), and one site in the 3′UTR. Then X axis denotes different vectors and the Y axis shows titer as determined in materials and methods. (C–F) $CD34^+$ HSPCs from two healthy donors were transduced in triplicate with vector constructs at 2×10^7 TU/mL and differentiated *in vitro* under erythroid conditions. Cells were collected at day 14 for (C) VCN analysis measured by ddPCR, and day 18 for (D) HbA^{AS3} and (E) HbF by HPLC, (F) total anti-sickling globin (%HbA^{AS3}-globin + %HbF) quantification measured by HPLC. Error bars represents means ± SD. (A) created with BioRender.

To determine whether combining these technologies could be accomplished with effective packaging and higher titers and improved gene transfer we cloned the *BCL11A* shmiR into the UV1 vector that expresses β^{AS3}-globin. To define the optimal location to incorporate the *BCL11A* shmiR into the UV1 vector, we cloned the *BCL11A* shmiR sequences into five different locations throughout the β^{AS3}-globin cassette. Multiple locations were selected due to the possibility of the shmiR disrupting β^{AS3}-globin RNA processing. The locations consisted of two sites in intron 1 (at the start of IVS1 and the end IVS1), two locations in intron 2 (intraΔ and the end IVS2), and one location in the 3′UTR (Figure 1A). Intron 2 had been previously modified by removing sequence that were detrimental to high titer vector production.[26] Removal of this region also decreased the length of the vector without significantly decreasing β^{AS3}-globin expression. These locations were further screened bioinformatically to avoid mRNA splicing and branchpoint sequences.

The resulting vector plasmids were Sanger sequenced to confirm correct construction and packaged using a HEK293T *PKR* knockout cell line. A host *PKR* response is initiated when transfecting with opposite oriented expression cassettes leading to inhibited synthesis of viral proteins resulting in lower titers.[28] This producer line is designed to yield higher titers from the vectors with reverse orientation expression cassettes and has been shown to increase titers of β^{AS3}-globin vectors by 2- to 5-fold.[28,29] We saw a minimal decrease in the unconcentrated viral titers with any of the five UV1-shmiR vectors in comparison to the UV1 control (Figure 1B). In particular, the shmiR in the end IVS2 position retained a comparable titer to the parental UV1 vector.

To assess the functionality of the vectors, we transduced $CD34^+$ cells at an MOI of 20 from two different healthy donors, each in triplicate, and performed erythroid differentiation. There was a mean VCN of 4.1 ± 2.0 for UV1 and a range of mean VCN from 2.7 ± 1.5 to 3.4 ± 1.4 for all of the UV1-shmiR vectors (Figure 1C).

High-performance liquid chromatography (HPLC) analysis of HGB species in the differentiated erythrocytes demonstrated that each of the UV1-shmiR vectors induced similar mean expression of β^{AS3}-globin per copy (3.9 ± 1.2% to 4.2 ± 0.8%) (Figure 1D), except for the vector with the *BCL11A* shmiR in the 3′UTR location. The UV1-shmiR vectors expressed 8.1 ± 0.9% to 14.4 ± 2.4% fetal globin per vector copy (Figure 1E). Percentages of total anti-sickling hemoglobins were calculated as $Hb\beta^{AS3}$ expression plus HbF expression. All UV1-shmiR combination vectors outperformed UV1 in the total anti-sickling hemoglobins produced.

From these data, we concluded that four out of the five locations performed well for inducing anti-sickling β^{AS3}-globin expression.

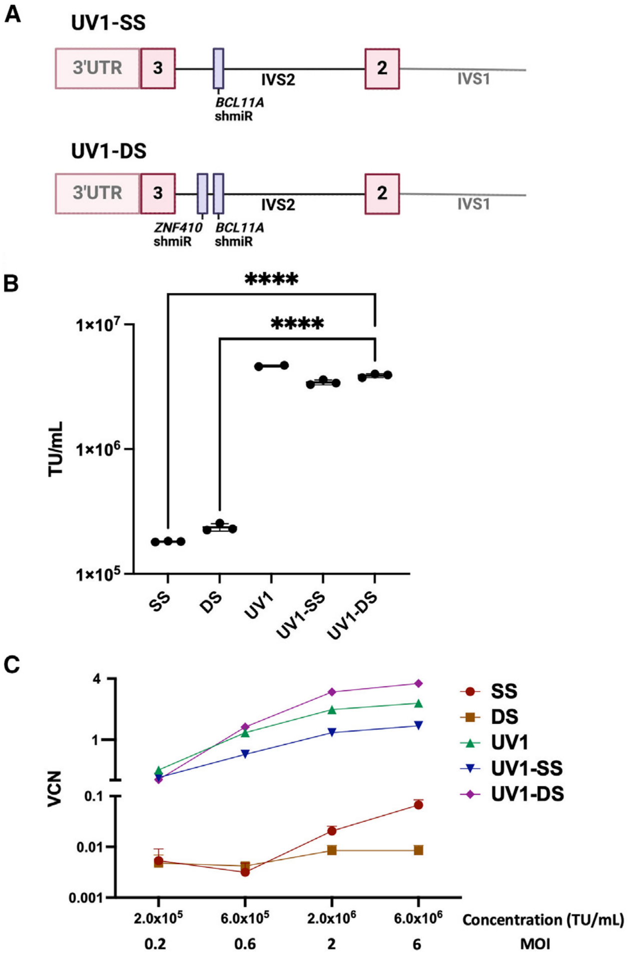

Figure 2. Maps, titers, and gene transfer efficiency of LVVs with SS or DS
(A) Map of **UV1-SS** (single shmiR) LVV containing the *BCL11A* shmiR at the end of IVS2 in the β^{AS3}-globin cassette. Map of **UV1-DS** (double shmiR) LVV containing both the *BCL11A* and *ZNF410* shmiR at the end of IVS2 in the β^{AS3}-globin cassette. Maps show detail of intron 1, site of shmiR insertion, and surrounding β-globin exons 2 and 3. (B) Vectors were packaged as described in Materials and methods and titers were determined on HT-29 cells and quantified with ddPCR. Unconcentrated viral titer shown here. **SS** and **DS** represent vectors with SS or DS, expressed in a β-globin LCR-driven LVV previously described.[13,30] Each point on the plot represents vector packaged and titered from an individual 10-cm plate. n = 3. (C) $CD34^+$ cells from a healthy donor were transduced with constructs at 2×10^5 TU/mL, 6×10^5 TU/mL, 2×10^6 TU/mL, and 6×10^6 TU/mL (MOIs of 0.2, 0.6, 2, and 6) and cultured for 14 days in myeloid differentiation conditions to assess levels of infectivity. VCN was measured by ddPCR. n = 2. Error bars represent means ± SD; Tukey's multiple comparisons test was performed on all arms with selected statistics shown. ****$p < 0.0001$. (A) created with BioRender.

The location at the End-IVS2 position (**UV1-SS**) was selected for further studies as it was the candidate vector that retained a high titer and high anti-sickling HGB expression. At a VCN of 1, UV1-SS led to a mean expression of 17.1% ± 2.4% anti-sickling hemoglobins (HbA^{AS3} plus HbF) compared with 5.7% ± 1.9% for UV1 (Figure 1F).[16]

UV1-DS, a double shmiR (DS) vector in the UV1 backbone incorporating the *ZNF410* shmiR with the *BCL11A* shmiR and β^{AS3}-globin

Incorporating the *ZNF410* shmiR with the *BCL11A* shmiR has been shown to increase fetal globin induction by approximately an additional 10%.[13] A vector was cloned to incorporate both the *BCL11A* and *ZNF410* shmiR at the END IVS2 location, creating a DS vector in the UV1 backbone (**UV1-DS**) (Figure 2A). The performance of five candidate vectors were then compared, including UV1-DS, UV1-SS, UV1, DS[13] (contains *ZNF410* and *BCL11A* shmiR), and single shmiR (SS)[30] (contains *BCL11A* shmiR). The shmiR sequences targeting *BCL11A* and *ZNF410* in the UV1 vectors are the same shmiR sequences in DS and SS. DS and SS will induce HbF expression, but are not in the UV1 backbone and, therefore, do not encode the β-globin gene. Vectors were packaged in a HEK293T *PKR* knockout cell line[8] and unconcentrated and concentrated viral titers were determined through transduction of the HT-29 cell line. Unconcentrated titers across all vectors in the UV1 backbone were comparable with mean titers between 3.4 ± 0.2e+06 TU/mL and 4.6 ± 0.1e+06 TU/mL (Figure 2B). The vectors containing shmiRs, but not in the UV1 backbone, had titers approximately 20-fold lower, with mean titers between 1.8 ± 0.0e+05 and 2.4 ± 0.0e+05 TU/mL (Figure 2B).

Gene transfer efficiency into healthy donor $CD34^+$ cells was analyzed using all five vectors. $CD34^+$ cells were transduced with concentrated viral supernatant at four different MOIs. Transduced $CD34^+$ cells were differentiated using myeloid cytokine stimulation (IL-3, IL-6, and ckit ligand) for 14 days and VCNs were measured using a droplet digital PCR (ddPCR) assay. Myeloid differentiation was used instead of erythroid differentiation, as a myeloid cell VCN has been demonstrated to more closely predict the VCN seen *in vivo* in bone marrow after xenotransplantation of immune-deficient mice.[12] Notably, the VCN of DS and SS plateaued, with increasing vector concentration not resulting in increased gene transfer. Vectors with the UV1 backbone had higher gene transfer to $CD34^+$ cells than the SS and DS vectors across a range of vector concentrations (Figure 2C).

In vitro assessment of the LVV using human SCD patient $CD34^+$ cells

To evaluate the potential therapeutic impact of this series of LVVs in SCD patients, peripheral blood (PB) $CD34^+$ cells from four different SCD donors were transduced with the five vectors at an MOI of 50. These cells were then subsequently differentiated *in vitro* using erythroid cytokines for 18 days after transduction. VCNs were determined by qPCR, anti-sickling HGB expression was determined by HPLC, and sickled cells were enumerated by microscopy after sodium metabisulfite (MBS) treatment. The VCNs generated with this MOI were in an appropriate range for comparison in subsequent analyses, with the VCNs of all experimental arms between an average of 1.3 ± 0.1 to 1.9 ± 0.2 (Figure 3A). To assess the functionality of UV1-DS in retaining the properties of *BCL11A* shmiR and *ZNF410* shmiR, we examined their knockdown efficiency by evaluating mRNA expression levels. As shown in Figures 3B and 3C, UV1 had no impact on the expression of *BCL11A* and *ZNF410*. In contrast, the groups that

included *BCL11A* shmiR (UV1-SS and SS) effectively suppressed *BCL11A* expression without affecting *ZNF410*. Notably, the groups incorporating both *BCL11A* and *ZNF410* shmiRs (UV1-DS and DS) exhibited a significant decrease in the expression of both *BCL11A* and *ZNF410*.

Each of the vectors induced expression of anti-sickling hemoglobins; the UV1-DS vector induced the highest levels of anti-sickling HGB (HbA^{AS3} and HbF) at an average of 57.5% ± 4.6%, while the UV1, SS, UV1-SS, and DS vectors induced anti-sickling globin expression at an average of 26.3% ± 0.1%, 23.5% ± 9.0%, 46.9% ± 0.6%, and 30.7% ± 9.7% respectively (Figure 3B). When normalized to the VCN, the UV1-DS vector induced the highest anti-sickling HGB expression per VCN (30.7% ± 2.5%), which was significantly higher compared with SS (18.1% ± 3.0%) (Figure 3C).

Sodium MBS treatment of transduced enucleated erythroid cells was associated with decreased percentages of sickled erythrocytes with all vectors tested compared with non-transduced ("mock") controls. Cells treated with UV1-DS showed the fewest sickled cells at an average of 14.6% ± 3.4%, while the average sickled cells in mock, UV1, SS, UV1-SS, DS were 58.8% ± 5.0%, 32.7% ± 0.4%, 37.8% ± 4.5%, 19.8% ± 2.9%, and 31.8% ± 6.0%, respectively (Figure 3D). Taken together, these data demonstrate that incorporating a DS vector in the UV1 backbone is feasible, further enhances anti-sickling HGB concentrations, and largely mitigates the cellular phenotype of erythrocyte sickling.

In vivo analysis of PB from Berkeley SCD mouse model

To determine whether the UV1-DS vector can ameliorate characteristic SCD disease cellular phenotypes *in vivo*, we utilized a transplantation model with Berkeley SCD (BERK-SCD) mouse HSPCs as donor cells (Figure S1). We designed a UV1-DS(m) for murine BM cells, replacing the *ZNF410* shmiR with a *Zfp410* shmiR. *Zfp410* shmiR targeted the murine sequence and transduction led to the knock down of *Zfp410* and induction of Hbb-y mRNA expression in mouse erythroid leukemia cells (Figure S2). We conducted a direct comparison of UV1-DS, UV1-SS, UV1, DS, and SS vectors at equivalent VCN after transduction of lin^- $CD45.2^+$ BM cells from BERK-SCD mice. Lin^- $CD45.2^+$ BM cells from BERK-SCD mice were pre-stimulated for 36–40 h and were transduced with vectors at different MOIs to achieve a VCN of 2 (based on prior assays). The transduced cells were injected into lethally irradiated (11 Gy) $CD45.^1$+ BL/6 (B6.SJL-Ptprca Pepcb/BoyJ) recipient animals 24 h after transduction. Two independent experiments were performed.

Engraftment was determined by flow cytometric enumeration of $CD45.2^+$ donor cells; HGB, hematocrit (HCT), and reticulocyte concentrations were measured on PB samples and the frequencies of $CD71^+Ter119^+$ erythroid precursors were measured by flow cytometry. The percentages of sickled RBCS were determined after sodium MBS treatment of blood samples *ex vivo*. PB was collected at weeks 4, 8, 12, and 16 weeks after transplantation (Figure S3).

Engraftment across all experimental arms was greater than 87% and did not significantly differ between different vectors (Figure 4A). The HGB and HCT levels at 16 weeks were not significantly different when recipients of UV1-DS transduced cells were compared with the healthy donor arm (Figures 4B and 4C). The HGB of the UV1-DS recipient mice averaged 11.7 ± 2.3 g/dL, while the HCT of the SS, UV1, and SCD arms averaged 9.8 ± 1.3 g/dL, 8.5 ± 0.5 g/dL, and 7.2 ± 0.4 g/dL, respectively. The HCT of the UV1-DS recipient mice averaged 46.0 ± 7.2%, while the HCT of the SS, UV1, and SCD arms averaged 38.5 ± 3.8%, 36.0 ± 3.1%, and 30.0 ± 1.4%, respectively.

As a sensitive indicator of hemolysis, we measured reticulocyte and erythroid precursor cell populations in the PB. The reticulocyte percentages and the frequency of erythroid precursor cells of the UV1-DS experimental arm (6.5% ± 2.4%, 5.6% ± 1.9%) showed no statistically significant difference ($p = 0.14$, $p = 0.39$) with the healthy control (2.3% ± 0.3%, 1.7% ± 0.6%) (Figures 4D and 4F). The UV1-DS arm showed a significant decrease ($p < 0.01$) in reticulocytes in comparison to the SS arm (6.5% ± 2.4% vs. 13.1% ± 2.9%) (Figure 4D).

Ex vivo quantification of sickled cells in blood harvested from mice at 16 weeks was performed. Red blood cells were exposed to 2% sodium MBS and incubated under hypoxic conditions at 37°C for 30 min and imaged. Mice transplanted with UV1-DS transduced cells showed an average of 10.9% ± 3.3% sickled cells, which was not statistically significantly different ($p = 0.07$) compared with the healthy control arm with an average of 5.2% ± 1.7% abnormally shaped cells (Figure 4E). These values were obtained with an average VCN of 1.5 cdg. Overall, PB analysis suggested that the UV1-DS recipient animals demonstrated some significant improvement in hematologic parameters compared with the SS group, with no significant differences compared with animal transplanted with control cells from healthy donors.

Analysis of bone marrow after gene therapy in the Berkeley SCD mouse model

Animals were sacrificed at 16 weeks for complete analyses, including BM and spleen studies. The average BM VCNs were similar among all of the experimental arms with a median of 1.5 VCN/diploid genome and a range of 0.5 (Figure 5A).

To determine if UV1-DS demonstrated therapeutic levels of anti-sickling HGB expression in BM cells, we measured β^{AS3}-globin and γ-globin RNA transcript levels by RT-qPCR in erythroid cells derived from BM purified by flow cytometry. We confirmed the SS and DS groups demonstrated increased γ-globin, while the UV1 group expressed β^{AS3}-globin and UV1-SS and UV1-DS induced both γ-globin and expressed β^{AS3}-globin (Figure 5B). The total anti-sickling globin mRNA content (γ-globin + β^{AS3}-globin) was determined, and the expression was normalized to the VCN in BM for each mouse. The highest level of anti-sickling globin mRNA expression was seen the UV1-DS group with an average of 29.0% ± 4.2% anti-sickling globin per 1 VCN/dg This expression was significantly higher than the DS

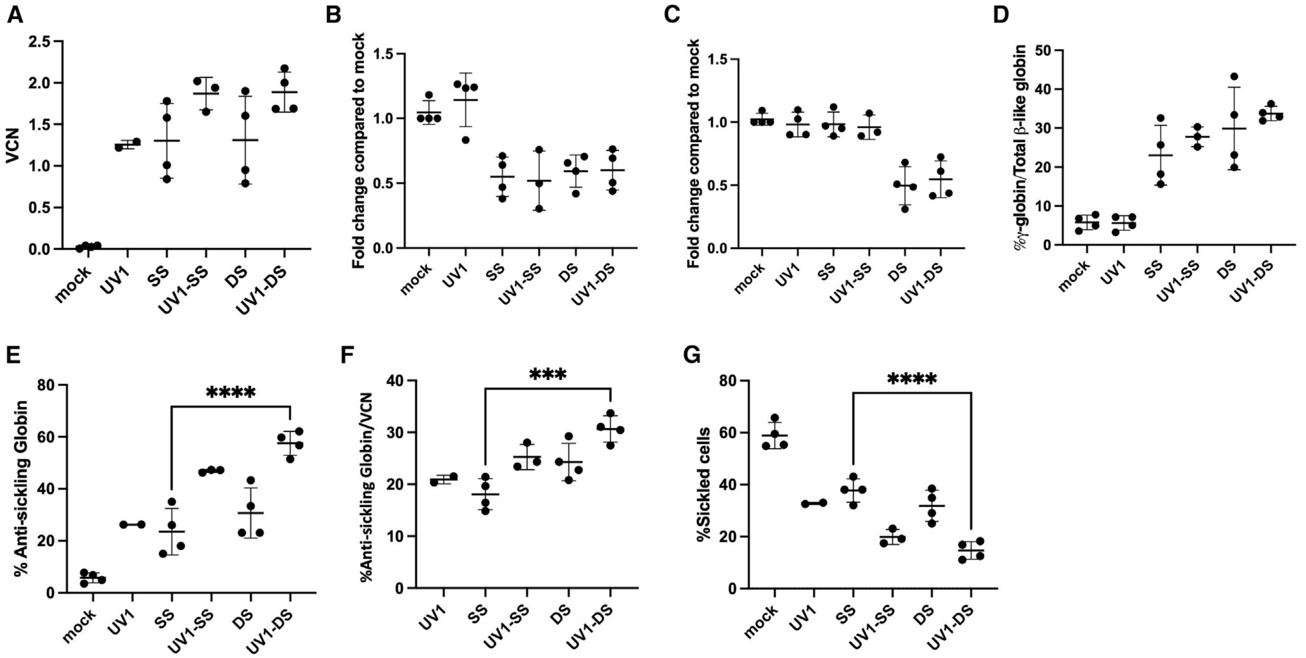

Figure 3. *In vitro* assessment of LVVs using human CD34+ cells derived from SCD patients

(A) Plerixafor-mobilized CD34+ HPSCs from patients with SCD were transduced with vectors at 1×10^7 TU/mL and then differentiated *in vitro* for 18 days in erythroid culture conditions. VCN of transduced cells were determined by qRT-PCR. (B) Expression was measured by qRT-PCR with GAPDH as control on day 11 of differentiation for *BCL11A* and (C) *ZNF410*. (D) Induction of gamma-globin mRNA and (E) anti-sickling globin (HBB^{AS3} + HBB) was determined on day 18 of differentiation by qRT-PCR. (F) Induction of anti-sickling globin (HBB^{AS3} + HBB) normalized to VCN (G) Enucleated red blood cells were enriched by fluorescence-activated cell sorting and treated with MBS to induce sickling. Quantification of % sickled cells of enucleated erythroid cells differentiated from transduced mock vector (control) and various test vectors was assessed by phase contrast microscopy 30 min after MBS treatment. Error bars represent means ± SD; each data point represents data from cells of an individual SCD patient; Tukey's multiple comparisons test was performed on all arms with selected statistics shown. ***$p < 0.001$, ****$p < 0.0001$.

and SS group at 18.4% ± 3.5% and 11.4% ± 2.8%, respectively ($p < 0.0001$) (Figure 5C).

Spleen weights were measured as an indication of compensatory erythroid expansion. The UV1-DS treated group showed an average spleen mass of 0.16 ± 0.05 g and was not significantly different than the healthy control arm of 0.09 ± 0.01 g. UV1-DS-treated spleen mass were significantly lower than the SS ($p < 0.05$) group at 0.25 ± 0.07 g (Figure 5D). Taken together, these data demonstrate that UV1-DS transduced cells led to a robust rescue of all SCD RBC phenotypes examined.

DISCUSSION

Gene therapies for SCD have made excellent advances in the past decade, with approaches using LVVs and CRISPR-Cas9 showing excellent clinical efficacy. A major limitation of gene therapy for SCD is the availability and access to a one-time potentially curative therapy for patients. With the cost of treatment being high ($1–$3 million per treatment) and the future reimbursement strategy for these expensive therapies not being clear, many individuals will face challenges in accessing approved therapies. One major factor is the high costs to produce LVVs that are produced at low titer and require a relatively high MOI for effective transduction of CD34+ HSPCs. The results presented here demonstrate the advantages in combining two technologies not only leading to increased efficacy, but also to increased titers and gene transfer, which may lead to lower production costs providing more accessible therapies.

Brendel et al.[21] previously showed high induction of fetal globin expression per copy number with a *BCL11A* shmiR, while Morgan et al.[9] showed that engineering smaller β^{AS3}-globin vectors can lead to higher titers. We wanted to investigate whether combining shmiR and β^{AS3}-globin technologies could lead to a superior therapy that had the combined benefits of potent anti-sickling globin expression with significantly increased titers and gene transfer. We first showed proof of concept with the incorporation of the *BCL11A* shmiR into the UV1 backbone. shmiRs integrated into the intronic regions of the β^{AS3}-globin cassette had minimal impact on β^{AS3}-globin protein expression, while incorporation into the 3′UTR region led to diminished expression of β^{AS3}-globin. We hypothesize that, when the shmiR is processed out from the transcript in the 3′UTR, the transcript is degraded, thereby decreasing the amount of β^{AS3}-globin expression. Since the other shmiRs are located in intronic regions, the introns will be spliced out before translation and we hypothesize that this is before the shmiR will be processed out, therefore having minimal impact on the β^{AS3}-globin transcript.

With the addition of the *BCL11A* shmiR, we witnessed fetal globin induction leading to an increase of total anti-sickling hemoglobins with the combination vectors. We did see that the incorporation of the shmiR led to a small decrease in gene transfer when transduced at equal transduction units in comparison with UV1. We hypothesized that the incorporation of the shmiR, which increases the length and complexity of the vector, could be leading to an increase of incomplete viral genomic RNAs. This concept has been previously studied, and the data have shown that β-globin LVV viral genomic RNAs can be incomplete and released in vector particles, leading to lower gene transfer in HSPCs.[8] We extracted viral RNA from UV1 and the UV1-shmiR unconcentrated viral supernatants and performed a ddPCR assay to assess the concentration of complete viral RNAs.[8] The data show a lower concentration of complete viral RNA in the UV1-shmiR vectors, which could explain lower gene transfer (Figure S4).

We formulated a hypothesis suggesting that shmiRs present in UV1 would exhibit greater titer and gene transfer than in their original backbones. Upon evaluating the titer and gene transfer of the UV1-DS vector, we found both to be comparable with those of the UV1 control. This indicates that the additional shmiR sequence did not significantly affect titer and gene transfer. Moreover, the variation in backbone sequence of UV1, characterized by its smaller size and different complexity, yielded advantages in terms of higher titer and gene transfer, compared with SS and DS in a longer backbone. Additionally, UV1-DS also showed a 2- to 8-fold increase in titer when packaged and titered head to head with alternative β^{AS3}-globin LVVs[11,31] (Figure S5). The UV1 backbone has a substantial benefit because lower transduction concentrations can be used to achieve the same VCN, meaning lower viral volumes will be needed, leading to less potential toxicity for the cells and reduced cost per patient dose. For example, a 5,00.-fold scale up from 1×10^5 to 5×10^8 for a hypothetical 75-kg patient and a CD34$^+$ cell dose of 6×10^6/kg would require the DS vector produced from 4 L for one patient dose versus 0.2 L for the UV1-DS vector; thus UV1-DS would yield 20-fold more patient doses per vector lot.

A crucial aspect in the development of this therapy involved evaluating whether the combination would result in improved efficacy. Previous research has demonstrated that achieving approximately 20% expression of anti-sickling hemoglobins leads to an improvement in the SCD phenotype.[32] Based on this, our hypothesis was that the UV1-DS vector would exhibit the highest expression of anti-sickling hemoglobins by combining β^{AS3}-globin expression and γ-globin induction, potentially allowing for a lower VCN requirement to ameliorate the disease. To evaluate the efficacy of the UV1-DS vector, we utilized human SCD CD34$^+$ cells and conducted erythroid differentiation. We observed the highest induction of anti-sickling hemoglobins in UV1-DS-treated cells (approximately 30% per VCN), resulting in a significant decrease in the sickling phenotype when compared with the UV1, SS, or DS vectors. Based on this experiment we would expect that human SCD CD34$^+$ cells transduced with UV1-DS at VCN of 1 would achieve therapeutic levels. This finding supports the notion that the combination therapy holds promise for effectively addressing SCD at a lower VCN, which in turn can lead to fewer safety concerns regarding insertional mutagenesis. In contrast, the inclusion of multiple shmiRs may increase the chance of off-target effects. Through sequence homology analysis, the probability of BCL11A and ZNF410 shmiRs having off-target effects seems to be low (data not shown). We previously reported differentially expressed genes after BCL11A and ZNF410 knockdown in erythroid cells[13,21,27] (Figure S6), but were unable to identify any undesirable side effects on cellular erythroid differentiation or cell physiology *in vitro* or *in vivo*.

We then assessed amelioration of hematologic parameters of the sickle phenotype in the BERK SCD mouse model. We excluded mice with less than 97% donor engraftment at week 16 from the analysis to prevent confounding of phenotype correction by residual wild-type erythrocytes. We made the decision to perform *in vivo* experimentation at equal VCN to allow for intrinsic anti-sickling activity of each vector to be determined. However, using different amounts of each vector to achieve a similar VCN obscures the potential advantages of smaller, higher titer vectors to transduce higher percentages of cells and achieve sufficient VCNs to impede sickling. Of note, experimental arms using LVV in the UV1 backbone required less vector volume for transduction to achieve the same VCN as the shmiR only vectors in a longer backbone (SS and DS), which suggests not only less potential cell toxicity when translated clinically, but also decreased the cost of therapy. All hematologic parameters assessed in the PB of recipients treated with UV1-DS showed significant improvements when compared with mock-treated and UV1-treated lineage-negative (lin$^-$) cells. Significant improvements in HCT and reticulocyte percentages were also seen when compared with the SS arm. When assessing BM, there was a significant increase in percentages of anti-sickling hemoglobins per VCN, as well as a significant decrease in the spleen weights of the UV1-DS-treated group in comparison with the UV1 and SS arms. These data emphasize that the UV1-DS vector has potential to be advantageous in a clinical setting in comparison with β^{AS3}-globin and shmiR technologies separately.

We also performed *in vivo* experimentation in the Townes SCD mouse model (Figures S7–S9), but saw minimal to no induction of HbF in mice treated with vectors containing shmiRs. A recent article that has characterized the Townes mouse model by long-range sequencing showed the lack of distal gene-regulatory elements that may be necessary for HbF induction.[33] Several human assay for transposase-accessible chromatin sequencing (ATAC-seq) signals seen in the human β-globin locus were not included the Townes mouse transgenes and are also not conserved in mice. These elements may be involved with HGB switching, a mechanism that does not naturally occur in mice, leading to the potential suboptimal expression of the *HBG1* transgene.[33] In addition, human ATAC-seq peaks for known gamma-globin regulators were also not seen in the Townes mouse transgene including: *HBBP1*[34] *and BGLT3*.[35,33] While *Bcl11a* and *Zfp410* may have

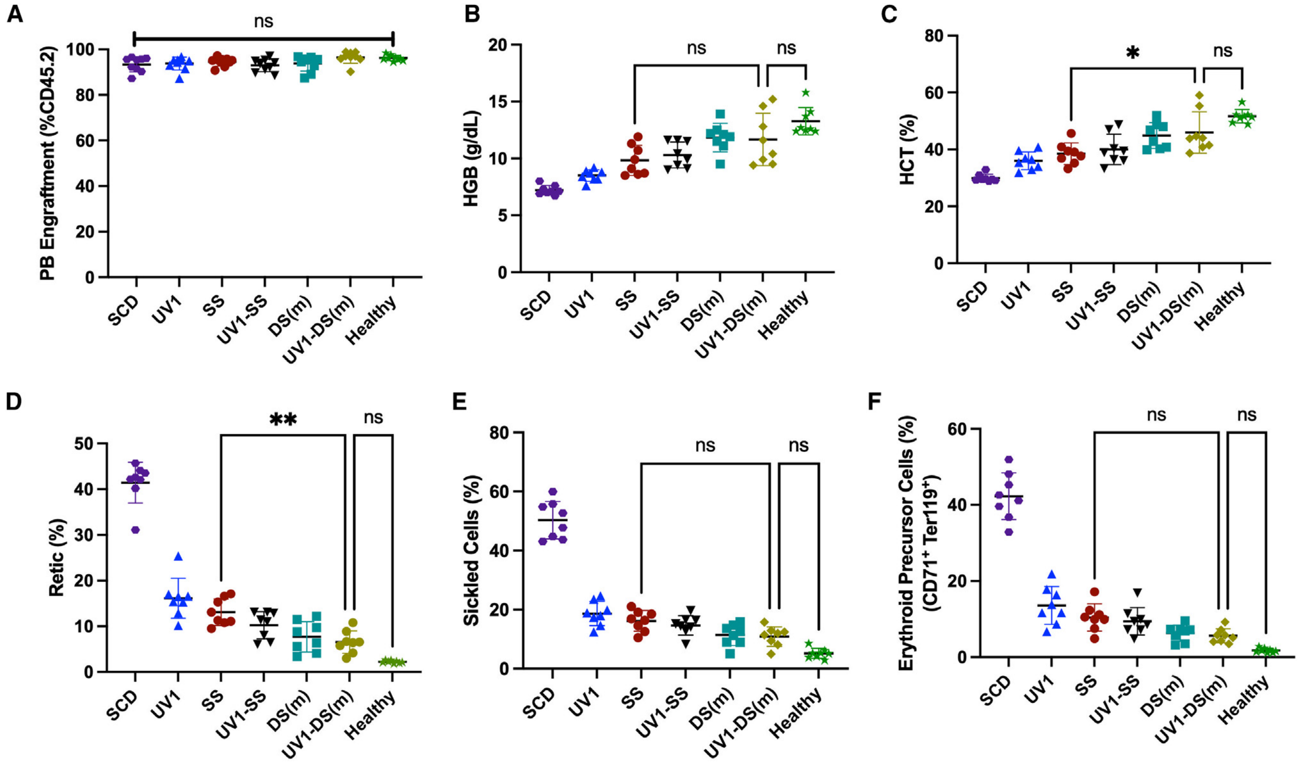

Figure 4. Correction of PB sickle cell hematologic parameters *in vivo* in Berkeley SCD mouse model

lin⁻ bone marrow cells from BERK mice (CD45.2) were transduced with each vector at MOIs adjusted to achieve similar VCN, or mock-transduced as controls, and transplanted into irradiated CD45.1$^+$ BL/6 mouse recipients. Mice were bled at 16 weeks after transplant and PB was analyzed. (A) Engraftment was assessed in PB by flow cytometry (%CD45.2$^+$ cells). (B) HGB (g/dL), (C) HCT, and (D) reticulocyte counts (%) are shown. (E) PB was treated *ex vivo* with MBS for 30 min to induce sickling. Percentage of sickled RBCs from PB sample was quantified. (F) Percentages of CD71$^+$ Ter119$^+$ high erythroid precursor cell population in PB. Error bars represent mean ± SD. Symbols indicate mice transplanted with different shmiR vectors or non-transduced cells (SCD); each data point represents an individual mouse, n = 8, ns, not significant; Tukey's multiple comparisons test was performed on all arms with selected statistics shown. *p < 0.05; **p < 0.01.

been successfully downregulated in our Townes model, we hypothesize that lack of key regulatory elements may be the reason we witnessed minimal HbF induction. Our results suggest that the Townes mouse is not an optimal model when assessing HbF induction by *BCL11A* and *Zfp410* shmiRs.

The incorporation of shmiRs into the UV1 backbone has yielded a vector that not only induces high anti-sickling HGB expression, but also exhibits higher titer and gene transfer capabilities, compared with earlier constructs. It is worth noting that previous clinical trials involving β^{AS3}-globin and *BCL11A* shmiR technology have shown considerable success, but by utilizing the UV1-DS vector, we can capitalize on the remarkable efficacy observed with shmiR technology, while simultaneously maintaining the high titers and gene transfer capabilities associated with the UV1 vector. Consequently, UV1-DS holds the potential for significant advantages in terms of efficacy, clinical scale production, and cost reduction for autologous gene therapy. These promising features make UV1-DS an exciting prospect for further exploration and potential implementation in future therapeutic approaches.

MATERIALS AND METHODS

Cloning and vector production

The UV1 vector, *BCL11A* shmiR, and *ZNF410* shmiR, *Zfp410* shmiR have been described previously.[9,13,21,27] To introduce the BCL11A shmiR, five sets of reverse-oriented primers with extended homology sequence were used to PCR amplify the UV1 plasmid backbone. The *BCL11A* shmiR oligos (Integrated DNA Technologies, San Diego, CA, USA) were combined and an oligo duplex was generated. With homology between the shmiR sequences and linearized plasmids, the shmiR sequences were joined using the NEBuilder HiFi DNA Assembly kit (New England Biolabs, Ipswich, MA, USA). All plasmids were sequence verified by Sanger sequencing (Laragen Inc, Culver City, CA, USA).

HEK293T *PKR* knockout cells[8] were plated on 10-cm plates at a density of 1 × 10^7 cells/mL and vectors were packaged through transient transfection, using third-generation lentiviral packaging plasmids.[36] Raw viral supernatant was collected 3 days after transfection, samples were used for titer determination, and the remainder of the viral supernatants were concentrated through ultracentrifugation. Titers

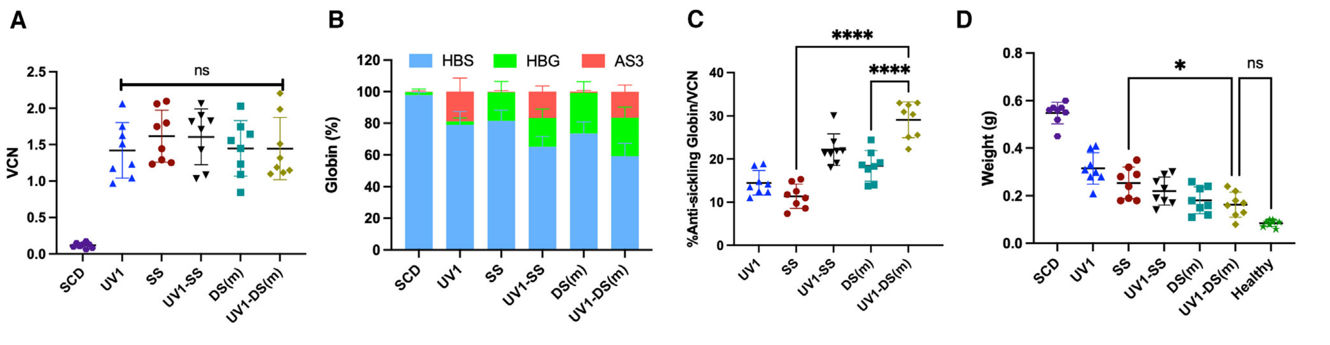

Figure 5. Anti-sickling HGB induction in bone marrow *in vivo* in the Berkeley mouse model

Mice were euthanized at 16 weeks after transplant and whole bone marrow (BM) and spleen was harvested and analyzed individually. (A) VCN in BM was determined by qPCR. (B) Percentages of globin mRNA expression of erythroid cells in BM determined by qRT-PCR. (C) Anti-sickling globin mRNA expression in erythroid cells in BM adjusted for VCN. (D) Spleen weights. Error bars represent mean ± SD. Each data point represents an individual mouse. ns, not significant; Tukey's multiple comparisons test was performed on all arms with selected statistics shown. $*p < 0.05$, $****p < 0.0001$.

were determined by performing transduction of HT-29 human colorectal carcinoma cell line at multiple dilutions of both raw and concentrated viral supernatant. Three days after transduction, cells were harvested and titers were calculated through VCN determination by ddPCR assay using primers and probes for HIV-1 PSI (forward 5′-AAGTAGTGTGTGCCCGTCTG-3′, reverse 5′-CCTCTGGTTTCCCTTTCGCT-3′, 5′-56-FAM-AGCTCTCTC-ZEN-GACGCAGGACTCGGC-3IABkFQ-3′) and the Human Syndecan 4 gene (SCD4) as a reference (forward 5′-CAGGGTCTGGGAGCCAAGT-3′, reverse 5′-GCACAGTGCTGGACATTGACA-3′, 5′-5HEX-CCCACCGAA-ZEN-CCCAAGAAACTAGAGGAGAAT-3IABkFQ-3′).

PB healthy CD34$^+$ transduction and erythroid differentiation

PB CD34$^+$ samples were obtained from healthy donors by plerixafor and granulocyte colony-stimulating factor (G-CSF) mobilization. Cells were thawed and plated at 1 × 10^6 cells/mL on non-tissue culture treated 96 well plates pre-coated with retronectin (20 μg/mL, Takara Shuzo, Otsu, Japan). Cells were pre-stimulated for 24 h in X-Vivo 15 medium (Lonza, Basel, Switzerland) supplemented with 1× glutamine, penicillin, and streptomycin (Gemini Bio-Products, Sacramento, CA, USA), human Flt-3 ligand (50 ng/mL), human stem cell factor (SCF) (50 ng/mL), human thrombopoietin (TPO) (50 ng/mL), and human IL-3 (20 ng/mL) (cytokines: PeproTech, Rocky Hill, NJ, USA). CD34$^+$ cells were transduced with concentrated viral supernatants at a transduction concentration of 2 × 10^7 TU/mL (MOI of 20), without additional transduction enhancers. At 24 h after transduction, the cells were washed and plated under erythroid culture conditions. At days 2–7 after transduction, the cells were cultured in erythroid differentiation base medium (EDM) consisting of Iscove modified Dulbecco's medium (Lonza), 1× glutamine, penicillin, and streptomycin (Gemini Bio-Products), Holo-human transferrin (330 μg/mL) (Sigma-Aldrich, Burlington, MA), recombinant human insulin (10 μg/mL) (Sigma-Aldrich), heparin (2 IU/mL) (Sigma-Aldrich), 5% human solvent detergent pooled plasma AB (Octapharma USA Inc., Paramus, NJ, USA) supplemented with hydrocortisone (1 μM) (Sigma-Aldrich), human IL-3 (5 ng/mL) (PeproTech), human SCF (100 ng/mL) (PeproTech), and erythropoietin (3 IU/mL) (Sigma-Aldrich). At days 8–10 after transduction, the cells were cultured in EDM supplemented with 3 IU/mL erythropoietin. At days 11–21 after transduction, the cells were cultured in EDM without added cytokines. Cells were collected 14 days after transduction and ddPCR assays were used to analyze VCN and mRNA expression for γ-globin and β^{AS3}-globin. At day 21, cells were collected for protein analysis using HPLC to assess adult HGB (HbA), HbF, and β^{AS3}-globin (Hbβ^{AS3}).

Myeloid dose response

PB CD34$^+$ samples were obtained from healthy donors from plerixafor and G-CSF mobilization. Cells were thawed and plated at 1 × 10^6 cells/mL on non-tissue culture-treated 96-well plates pre-coated with retronectin (20 μg/mL; Takara Shuzo). Cells were pre-stimulated for 24 h in X-Vivo 15 medium (Lonza) supplemented with 1× glutamine, penicillin, and streptomycin (Gemini Bio-Products), human Flt-3 ligand (50 ng/mL), human SCF (50 ng/mL), human TPO (50 ng/mL), and human IL-3 (20 ng/mL) (cytokines: PeproTech). CD34$^+$ cells were transduced with concentrated viral supernatants at transduction concentrations of 2 × 10^5 TU/mL, 6 × 10^5 TU/mL, 2 × 10^6 TU/mL, and 6 × 10^6 TU/mL with transduction enhancer Poloxamer 338 (1 mg/mL) (BASF, Ludwigshafen, Germany). At 24 h after transduction, cells were washed and plated under myeloid culture conditions. Cells were cultured for 2 weeks after transduction in basal bone marrow media consisting of Iscove-modified Dulbecco's medium (Lonza) 1× glutamine, penicillin, and streptomycin (Gemini Bio-Products), 20% fetal bovine serum, and 0.52% BSA (Sigma-Aldrich) supplemented with human SCF (25 ng/mL), human IL-3 (5 ng/mL), and human IL-6 (10 ng/mL).

Transduction of human SCD patient CD34$^+$ cells

SCD patient CD34$^+$ HSPCs were isolated from unmobilized PB following receiving Boston Children's Hospital institutional review board approval and informed patient consent. The SCD CD34$^+$ HSPCs were enriched using the Miltenyi CD34 Microbead kit (Miltenyi Biotec, Auburn, CA, USA). CD34$^+$ cells were prestimulated for 36–40 h at 1 × 10^6 cells/mL in Stem Cell Growth Medium

(CellGenix, Portsmouth, NH, USA) supplemented with SCF, FMS-like tyrosinekinase 3 ligand, and TPO, all from Peprotech. Cells were then enumerated and transduced with the vector at an MOI as indicated in presence of LentiBOOST enhancer (SIRION Biotech, Gräfelfing, Germany) for 24 h before downstream processing.

In vitro erythroid differentiation of SCD $CD34^+$ cells

The *in vitro* erythroid differentiation protocol used is based on a three phase protocol adapted from Giarratana et al.[37] The cells were cultured in erythroid differentiation medium (EDM) consisting of Iscove modified Dulbecco's medium (Cellgro, Manassas, VA, USA) supplemented with 1% L-glutamine (Thermo Fisher Scientific, Waltham, MA, USA), and 1% penicillin-streptomycin (Thermo Fisher Scientific), 330 mg/mL holo-human transferrin (Sigma-Aldrich), 10 mg/mL recombinant human insulin (Sigma-Aldrich), 2 IU/mL heparin (Sigma-Aldrich), 5% human solvent detergent pooled plasma AB (Rhode Island Blood Center, Providence, RI, USA), and 3 IU/mL erythropoietin (Amgen, Thousand Oaks, CA, USA). During the first phase of expansion (days 0–7), $CD34^+$ cells were cultured in EDM in the presence of 10^6 mol/L hydrocortisone (Sigma-Aldrich), 100 ng/mL SCF (Peprotech), 5 ng/mL IL-3 (R&D Systems, Minneapolis, MN, USA), as EDM-1. In the second phase (days 7–11), the cells were resuspended in EDM supplemented with SCF, as EDM-2. For the third phase (days 11–18), the cells were cultured in EDM without additional supplements, as EDM-3.

In vitro sickling assay

At the completion of erythroid differentiation, enucleated RBCs were sorted, with the use of Hoechst 33342 (5 mg/mL; Invitrogen, Waltham, MA, USA), and subjected to an *in vitro* sickling assay. Sickling was induced by adding 500 mL freshly prepared 2% sodium MBS (Sigma-Aldrich) solution prepared in PBS into enucleated cells resuspended with 500 mL EDM-3 in a 24-well plate, followed by incubation at 37°C for 30 min. Live cell images were acquired using a Nikon Eclipse Ti inverted microscope (Nikon, Tokyo, Japan). More than 500 cells were counted for each sample; cells with an irregular structure, protruding spikes, or sickle shape were counted as sickling cells.

In vivo experiment in the SCD mouse model

Lin^- mouse BM cells were isolated by flushing femurs, tibias, and iliac crests of 6- to 8-week-old CD45.2 C57BL/6 or CD45.2 Berkeley SCD mice (BERK-SCD, JAX stock #003342) followed by lineage depletion using the Mouse Lineage Cell Depletion Kit (Miltenyi Biotec, Bergisch Gladbach, Germany). Lin− cells were pre-stimulated at 1×10^6 cells/mL in Stem Cell Growth Medium (CellGenix) supplemented with mouse SCF (100 ng/mL), hTPO (100 ng/mL), mouse IL-3 (mIL-3) (20 ng/mL), and hFlt3-L (100 ng/mL), all from Peprotech. Following a 36- 40-h pre-stimulation, cells were transduced at a density of 1×10^6 cells/mL in the presence of LentiBOOST enhancer, and transduced cells (without sorting) were transplanted by retro-orbital injection into lethally irradiated (7 + 4 Gy, split dose) CD45.1 recipients (B6.SJL-Ptprca Pepcb/BoyJ, Jax Strain #002014) 24 h after transduction. PB samples were collected at weeks 4, 8, 12, and 16 to measure engraftment by flow cytometry (CD45.2/CD45.1), determine RBC indices, and quantitate sickled cells. At week 16, mice were euthanized, and BM cells were used to measure engraftment by flow cytometry (CD45.2/CD45.1), VCN, and mRNA expression, spleens were collected to weigh. All animal experiments were approved by the Boston Children's Hospital's Institutional Animal Care and Use Committee.

VCN assay for the BERK mouse studies

Genomic DNA was extracted using the QIAGEN DNeasy protocol. VCN was assessed by qRT-PCR, performed with the use of TaqMan Fast Advanced Master Mix (Applied Biosystems, Waltham, MA, USA). VCN was calculated by using primers and probes HIV-1 PSI (forward 5′-CAGGACTCGGCTTGCTGAAG-3′, reverse 5′-TCCCCCGCTTAATACTGACG-3′, probe FAM-50-CGCACGGCAAGAGGCGAGG-3′) as a target and the human glycosyltransferase Like Domain Containing 1 gene (GTDC1) as an internal reference standard (forward 5′-GAAGTTCAGGTTAATTAGCTGCTG-3′, reverse 5′-TGGCACCTTAACATTTGGTTCTG-3′, probe VIC-5′-ACGAACTTCTTGGAGTTGTTTGCT-3′). Standard curves were obtained by serial dilutions of a plasmid containing one copy of PSI and GTDC1 sequences. The number of PSI and GTDC1 copies in test samples was extrapolated from the standard curves.

DATA AND CODE AVAILABILITY

Data available from Dr. Donald B. Kohn upon request.

SUPPLEMENTAL INFORMATION

Supplemental information can be found online at https://doi.org/10.1016/j.omtm.2024.101254.

ACKNOWLEDGMENTS

This work was supported by the Bill and Melinda Gates Foundation (INV-050202). Training grants provided support to K.L.H. (NIH TL1 DK132768 and U2C DK129496). We thank Yu Zhou for bioinformatics analysis assistance.

AUTHOR CONTRIBUTIONS

K.L.H., B.L., D.B.K., and D.A.W. designed experiments. K.L.H. and B.L. executed and analyzed experiments. D.B., B.C.F, K.T., and K.O. helped execute portions of experiments. D.B.K., D.A.W., R.P.H., B.C.F., and C.B. advised experiments. K.L.H., B.L., D.B.K., and D.A.W. wrote the manuscript.

DECLARATION OF INTERESTS

The authors declare a patent related to this work (Application Number: USSN 63/319,152). D.A.W. has received research funding from bluebird bio research in hemoglobinopathies. Boston Children's Hospital has licensed certain intellectual property (IP) relevant to hemoglobinopathies to bluebird bio. The bifunctional anti-sickling LVVs described here are the subject of patent applied for by University of California, Los Angeles and Boston Children's Hospital.

REFERENCES

1. CDC (2020). Complications and Treatments of Sickle Cell Disease | CDC (Centers for Disease Control and Prevention). https://www.cdc.gov/ncbddd/sicklecell/treatments.html.
2. Hassell, K.L. (2010). Population Estimates of Sickle Cell Disease in the U.S. Am. J. Prev. Med. *38*, S512–S521. https://doi.org/10.1016/j.amepre.2009.12.022.
3. Charache, S., Terrin, M.L., Moore, R.D., Dover, G.J., Barton, F.B., Eckert, S.V., McMahon, R.P., and Bonds, D.R. (1995). Effect of hydroxyurea on the frequency of painful crises in sickle cell anemia. Investigators of the Multicenter Study of Hydroxyurea in Sickle Cell Anemia. N. Engl. J. Med. *332*, 1317–1322. https://doi.org/10.1056/NEJM199505183322001.
4. Niihara, Y., Miller, S.T., Kanter, J., Lanzkron, S., Smith, W.R., Hsu, L.L., Gordeuk, V.R., Viswanathan, K., Sarnaik, S., Osunkwo, I., et al. (2018). A Phase 3 Trial of l-Glutamine in Sickle Cell Disease. N. Engl. J. Med. *379*, 226–235. https://doi.org/10.1056/NEJMoa1715971.
5. Ataga, K.I., Kutlar, A., Kanter, J., Liles, D., Cancado, R., Friedrisch, J., Guthrie, T.H., Knight-Madden, J., Alvarez, O.A., Gordeuk, V.R., et al. (2017). Crizanlizumab for the Prevention of Pain Crises in Sickle Cell Disease. N. Engl. J. Med. *376*, 429–439. https://doi.org/10.1056/NEJMoa1611770.
6. Vichinsky, E., Hoppe, C.C., Ataga, K.I., Ware, R.E., Nduba, V., El-Beshlawy, A., Hassab, H., Achebe, M.M., Alkindi, S., Brown, R.C., et al. (2019). A Phase 3 Randomized Trial of Voxelotor in Sickle Cell Disease. N. Engl. J. Med. *381*, 509–519. https://doi.org/10.1056/NEJMoa1903212.
7. May, C., Rivella, S., Callegari, J., Heller, G., Gaensler, K.M., Luzzatto, L., and Sadelain, M. (2000). Therapeutic haemoglobin synthesis in beta-thalassaemic mice expressing lentivirus-encoded human beta-globin. Nature *406*, 82–86. https://doi.org/10.1038/35017565.
8. Han, J., Tam, K., Ma, F., Tam, C., Aleshe, B., Wang, X., Quintos, J.P., Morselli, M., Pellegrini, M., Hollis, R.P., and Kohn, D.B. (2021). β-Globin Lentiviral Vectors Have Reduced Titers due to Incomplete Vector RNA Genomes and Lowered Virion Production. Stem Cell Rep. *16*, 198–211. https://doi.org/10.1016/j.stemcr.2020.10.007.
9. Morgan, R.A., Unti, M.J., Aleshe, B., Brown, D., Osborne, K.S., Koziol, C., Ayoub, P.G., Smith, O.B., O'Brien, R., Tam, C., et al. (2020). Improved Titer and Gene Transfer by Lentiviral Vectors Using Novel, Small β-Globin Locus Control Region Elements. Mol. Ther. *28*, 328–340. https://doi.org/10.1016/j.ymthe.2019.09.020.
10. Seakins, M., Gibbs, W.N., Milner, P.F., and Bertles, J.F. (1973). Erythrocyte Hb-S Concentration AN IMPORTANT FACTOR IN THE LOW OXYGEN AFFINITY OF BLOOD IN SICKLE CELL ANEMIA. J. Clin. Invest. 52, 422–432.
11. Levasseur, D.N., Ryan, T.M., Pawlik, K.M., and Townes, T.M. (2003). Correction of a mouse model of sickle cell disease: lentiviral/antisickling beta-globin gene transduction of unmobilized, purified hematopoietic stem cells. Blood *102*, 4312–4319. https://doi.org/10.1182/blood-2003-04-1251.
12. Romero, Z., Urbinati, F., Geiger, S., Cooper, A.R., Wherley, J., Kaufman, M.L., Hollis, R.P., de Assin, R.R., Senadheera, S., Sahagian, A., et al. (2013). β-globin gene transfer to human bone marrow for sickle cell disease. J. Clin. Invest. *67930*. https://doi.org/10.1172/JCI67930.
13. Liu, B., Brendel, C., Vinjamur, D.S., Zhou, Y., Harris, C., McGuinness, M., Manis, J.P., Bauer, D.E., Xu, H., and Williams, D.A. (2022). Development of a double shmiR lentivirus effectively targeting both BCL11A and ZNF410 for enhanced induction of fetal hemoglobin to treat β-hemoglobinopathies. Mol. Ther. *30*, 2693–2708. https://doi.org/10.1016/j.ymthe.2022.05.002.
14. Platt, O.S., Thorington, B.D., Brambilla, D.J., Milner, P.F., Rosse, W.F., Vichinsky, E., and Kinney, T.R. (1991). Pain in Sickle Cell Disease. N. Engl. J. Med. *325*, 11–16. https://doi.org/10.1056/NEJM199107043250103.
15. Castro, O., Brambilla, D.J., Thorington, B., Reindorf, C.A., Scott, R.B., Gillette, P., Vera, J.C., and Levy, P.S. (1994). The acute chest syndrome in sickle cell disease: incidence and risk factors. The Cooperative Study of Sickle Cell Disease. Blood *84*, 643–649. https://doi.org/10.1182/blood.V84.2.643.643.
16. Platt, O.S., Brambilla, D.J., Rosse, W.F., Milner, P.F., Castro, O., Steinberg, M.H., and Klug, P.P. (1994). Mortality In Sickle Cell Disease – Life Expectancy and Risk Factors for Early Death. N. Engl. J. Med. *330*, 1639–1644. https://doi.org/10.1056/NEJM199406093302303.
17. Murray, N., Serjeant, B.E., and Serjeant, G.R. (1988). Sickle cell-hereditary persistence of fetal haemoglobin and its differentiation from other sickle cell syndromes. Br. J. Haematol. *69*, 89–92. https://doi.org/10.1111/j.1365-2141.1988.tb07607.x.
18. Uda, M., Galanello, R., Sanna, S., Lettre, G., Sankaran, V.G., Chen, W., Usala, G., Busonero, F., Maschio, A., Albai, G., et al. (2008). Genome-wide association study shows BCL11A associated with persistent fetal hemoglobin and amelioration of the phenotype of beta-thalassemia. Proc. Natl. Acad. Sci. USA *105*, 1620–1625. https://doi.org/10.1073/pnas.0711566105.
19. Menzel, S., Garner, C., Gut, I., Matsuda, F., Yamaguchi, M., Heath, S., Foglio, M., Zelenika, D., Boland, A., Rooks, H., et al. (2007). A QTL influencing F cell production maps to a gene encoding a zinc-finger protein on chromosome 2p15. Nat. Genet. *39*, 1197–1199. https://doi.org/10.1038/ng2108.
20. Xu, J., Peng, C., Sankaran, V.G., Shao, Z., Esrick, E.B., Chong, B.G., Ippolito, G.C., Fujiwara, Y., Ebert, B.L., Tucker, P.W., and Orkin, S.H. (2011). Correction of sickle cell disease in adult mice by interference with fetal hemoglobin silencing. Science *334*, 993–996. https://doi.org/10.1126/science.1211053.
21. Brendel, C., Negre, O., Rothe, M., Guda, S., Parsons, G., Harris, C., McGuinness, M., Abriss, D., Tsytsykova, A., Klatt, D., et al. (2020). Preclinical Evaluation of a Novel Lentiviral Vector Driving Lineage-Specific BCL11A Knockdown for Sickle Cell Gene Therapy. Mol. Ther. Methods Clin. Dev. *17*, 589–600. https://doi.org/10.1016/j.omtm.2020.03.015.
22. Esrick, E.B., Lehmann, L.E., Biffi, A., Achebe, M., Brendel, C., Ciuculescu, M.F., Daley, H., MacKinnon, B., Morris, E., Federico, A., et al. (2021). Post-Transcriptional Genetic Silencing of BCL11A to Treat Sickle Cell Disease. N. Engl. J. Med. *384*, 205–215. https://doi.org/10.1056/NEJMoa2029392.
23. Vinjamur, D.S., Yao, Q., Cole, M.A., McGuckin, C., Ren, C., Zeng, J., Hossain, M., Luk, K., Wolfe, S.A., Pinello, L., and Bauer, D.E. (2021). ZNF410 represses fetal globin by singular control of CHD4. Nat. Genet. *53*, 719–728. https://doi.org/10.1038/s41588-021-00843-w.
24. Lan, X., Ren, R., Feng, R., Ly, L.C., Lan, Y., Zhang, Z., Aboreden, N., Qin, K., Horton, J.R., Grevet, J.D., et al. (2021). ZNF410 Uniquely Activates the NuRD Component CHD4 to Silence Fetal Hemoglobin Expression. Mol. Cell *81*, 239–254.e8. https://doi.org/10.1016/j.molcel.2020.11.006.
25. Brusson, M., Chalumeau, A., Martinucci, P., Romano, O., Felix, T., Poletti, V., Scaramuzza, S., Ramadier, S., Masson, C., Ferrari, G., et al. (2023). Novel lentiviral vectors for gene therapy of sickle cell disease combining gene addition and gene silencing strategies. Mol. Ther. Nucleic Acids *32*, 229–246. https://doi.org/10.1016/j.omtn.2023.03.012.
26. Sadelain, M., Wang, C.H., Antoniou, M., Grosveld, F., and Mulligan, R.C. (1995). Generation of a high-titer retroviral vector capable of expressing high levels of the human beta-globin gene. Proc. Natl. Acad. Sci. USA *92*, 6728–6732. https://doi.org/10.1073/pnas.92.15.6728.
27. Guda, S., Brendel, C., Renella, R., Du, P., Bauer, D.E., Canver, M.C., Grenier, J.K., Grimson, A.W., Kamran, S.C., Thornton, J., et al. (2015). miRNA-embedded shRNAs for Lineage-specific BCL11A Knockdown and Hemoglobin F Induction. Mol. Ther. *23*, 1465–1474. https://doi.org/10.1038/mt.2015.113.
28. Hu, P., Bi, Y., Ma, H., Suwanmanee, T., Zeithaml, B., Fry, N.J., Kohn, D.B., and Kafri, T. (2018). Superior lentiviral vectors designed for BSL-0 environment abolish vector mobilization. Gene Ther. *25*, 454–472. https://doi.org/10.1038/s41434-018-0039-2.
29. Han, J., Tam, K., Tam, C., Hollis, R.P., and Kohn, D.B. (2021). Improved lentiviral vector titers from a multi-gene knockout packaging line. Mol. Ther. Oncolytics *23*, 582–592. https://doi.org/10.1016/j.omto.2021.11.012.
30. Brendel, C., Guda, S., Renella, R., Bauer, D.E., Canver, M.C., Kim, Y.-J., Heeney, M.M., Klatt, D., Fogel, J., Milsom, M.D., et al. (2016). Lineage-specific BCL11A knockdown circumvents toxicities and reverses sickle phenotype. J. Clin. Invest. *126*, 3868–3878. https://doi.org/10.1172/JCI87885.
31. Poletti, V., Urbinati, F., Charrier, S., Corre, G., Hollis, R.P., Campo Fernandez, B., Martin, S., Rothe, M., Schambach, A., Kohn, D.B., and Mavilio, F. (2018). Pre-clinical Development of a Lentiviral Vector Expressing the Anti-sickling βAS3 Globin for

Gene Therapy for Sickle Cell Disease. Mol. Ther. Methods Clin. Dev. *11*, 167–179. https://doi.org/10.1016/j.omtm.2018.10.014.

32. Abraham, A., Hsieh, M., Eapen, M., Fitzhugh, C., Carreras, J., Keesler, D., Guilcher, G., Kamani, N., Walters, M.C., Boelens, J.J., et al. (2017). Relationship between Mixed Donor–Recipient Chimerism and Disease Recurrence after Hematopoietic Cell Transplantation for Sickle Cell Disease. Biol. Blood Marrow Transplant. *23*, 2178–2183. https://doi.org/10.1016/j.bbmt.2017.08.038.

33. Woodard, K.J., Doerfler, P.A., Mayberry, K.D., Sharma, A., Levine, R., Yen, J., Valentine, V., Palmer, L.E., Valentine, M., and Weiss, M.J. (2022). Limitations of mouse models for sickle cell disease conferred by their human globin transgene configurations. Dis. Model. Mech. *15*, dmm049463. https://doi.org/10.1242/dmm.049463.

34. Huang, P., Keller, C.A., Giardine, B., Grevet, J.D., Davies, J.O.J., Hughes, J.R., Kurita, R., Nakamura, Y., Hardison, R.C., and Blobel, G.A. (2017). Comparative analysis of three-dimensional chromosomal architecture identifies a novel fetal hemoglobin regulatory element. Genes Dev. *31*, 1704–1713. https://doi.org/10.1101/gad.303461.117.

35. Ivaldi, M.S., Diaz, L.F., Chakalova, L., Lee, J., Krivega, I., and Dean, A. (2018). Fetal γ-globin genes are regulated by the BGLT3 long noncoding RNA locus. Blood *132*, 1963–1973. https://doi.org/10.1182/blood-2018-07-862003.

36. Dull, T., Zufferey, R., Kelly, M., Mandel, R.J., Nguyen, M., Trono, D., and Naldini, L. (1998). A Third-Generation Lentivirus Vector with a Conditional Packaging. System *72*, 8463–8471. https://doi.org/10.1128/jvi.72.11.8463-8471.1998.

37. Giarratana, M.-C., Kobari, L., Lapillonne, H., Chalmers, D., Kiger, L., Cynober, T., Marden, M.C., Wajcman, H., and Douay, L. (2005). Ex vivo generation of fully mature human red blood cells from hematopoietic stem cells. Nat. Biotechnol. *23*, 69–74. https://doi.org/10.1038/nbt1047.

OMTM, Volume *32*

Supplemental information

A novel high-titer, bifunctional lentiviral vector for autologous hematopoietic stem cell gene therapy of sickle cell disease

Kevyn L. Hart, Boya Liu, Devin Brown, Beatriz Campo-Fernandez, Kevin Tam, Katherine Orr, Roger P. Hollis, Christian Brendel, David A. Williams, and Donald B. Kohn

The Townes SCD mouse model shows poor induction of HbF.

We performed a study to assess the activity of the UV1-SS and UV1-DS vectors in the Townes mouse SCD murine model, which reproduce several hematologic and pathophysiologic aspects of clinical disease.[1,2] We first cloned fetal-globin positive control vectors that only express gamma-globin, by knocking out the β^{AS3}-globin open reading frame in the UV1-SS and UV1-DS vectors, creating UV1-SS (No AS3) and UV1-DS (No AS3). To generate the controls all methionine codons (ATG) in the β^{AS3}-globin open reading frame were mutated. Site-directed mutagenesis was used to change the ATG start codon as well as a downstream methionine codon in exon 2 (Figure S7A). The single or double shmiR^{s} were left unchanged.

We validated the fetal globin positive controls by performing an erythroid differentiation using the parental HUDEP-2 cell line. The cells were transduced with vectors at 3.0×10^{5} TU/mL without transduction enhancers to aim for a VCN of 1-2. The cells were differentiated into erythrocytes and were collected on day 7 for VCN analysis and on day 12 for protein. Protein was analyzed by HPLC (Figure S7B, S7C). The HPLC data shows that UV1-SS (No AS3) and UV1-DS (No AS3) do not express Hbβ^{AS3}, while HbF is induced, suggesting that β^{AS3}-globin has been knocked out and will be appropriate controls in the UV1 backbone.

The control vectors were packaged and concentrated virus was generated through ultra-centrifugation. Titer was determined for raw and concentrated supernatant through HT-29 transduction (Figure S7D). A myeloid dose response with Lin- cells was performed to determine which concentration to use for each vector (UV1, UV1-SS, UV1-DS, UV1-SS (No AS3), UV1-DS (No AS3)) to obtain a VCN around 4 for Townes mouse studies Figure S7E).

Lin- Townes mouse BM cells were pre-stimulated at 1×10^{6} cells/mL and transduced 24 hours later. 24 hours post transduction the cells were transplanted by retro-orbital injection into lethally irradiated B6 CD45.1 recipients. PB, BM, and spleen were collected at week 16 for analysis.

There was a small induction of HbF in red blood cells in mice treated with vectors containing the single or double shmiR^{s} after intracellular staining of HbF and flow cytometry analysis (Figure S8B). However, there were not significant differences with the hematologic indices or spleen weights in untransduced SCD recipients (Figure S8C).

These data demonstrate that the Townes mouse model does not induce HbF expression to a functional level when knocking down *Bcl11a* and *Zfp410* with shmiR^{s}, compared to the degree of HbF induction achieved in human HSPC. Due to minimal induction of HbF, we did not see correction of the erythroid manifestations of SCD in the Townes mouse model. Woodard et al.[3] observed a similar blunted induction of HbF in Townes mouse HSPC that were treated with CRISPR/Cas9 nuclease to disrupt gamma-globin gene promoter sites.

Table S1: *BCL11A* shmiR insertion sequences in the β^{AS3}-globin cassette.

ShmiR Location	DNA Insertion Site: shmiR Sequence + 20 Base Flanking Sequences
Start-IVS1	caagacaggtttaaggagac**GATCTCACTTCCCCACAGAAGCTCTTGGCCTGGCCTC<br>CTGCAGTGCCACGCTGCGCGATCGAGTGTTGAATAACTCCATGTGGTAGAG<br>TTATTCAACACTCGATCGCGCAGTGCGGCACATGCTTACCAGCTCTAGGCC<br>AGGGCAGATGGGATATGACGAATGGACTGCCAGCTGGATACAAGGATGCT<br>CACC**caatagaaactgggcatgtg
End-IVS1	actctctctgcctattggtc**GATCTCACTTCCCCACAGAAGCTCTTGGCCTGGCCTCCT<br>GCAGTGCCACGCTGCGCGATCGAGTGTTGAATAACTCCATGTGGTAGAGTT<br>ATTCAACACTCGATCGCGCAGTGCGGCACATGCTTACCAGCTCTAGGCCAG<br>GGCAGATGGGATATGACGAATGGACTGCCAGCTGGATACAAGGATGCTCA<br>CC**tattttcccacccttaggct
IntraΔ-IVS2	gaaggggagaagtaacaggg**GATCTCACTTCCCCACAGAAGCTCTTGGCCTGGCCT<br>CCTGCAGTGCCACGCTGCGCGATCGAGTGTTGAATAACTCCATGTGGTAGA<br>GTTATTCAACACTCGATCGCGCAGTGCGGCACATGCTTACCAGCTCTAGGC<br>CAGGGCAGATGGGATATGACGAATGGACTGCCAGCTGGATACAAGGATGC<br>TCACC**tatttctgcatataaattgt
End-IVS2	tttgctaatcatgttcatac**GATCTCACTTCCCCACAGAAGCTCTTGGCCTGGCCTCCTG<br>CAGTGCCACGCTGCGCGATCGAGTGTTGAATAACTCCATGTGGTAGAGTTA<br>TTCAACACTCGATCGCGCAGTGCGGCACATGCTTACCAGCTCTAGGCCAGG<br>GCAGATGGGATATGACGAATGGACTGCCAGCTGGATACAAGGATGCTCAC<br>C**ctcttatcttcctcccacag
3'UTR	tccaactactaaactggggg**GATCTCACTTCCCCACAGAAGCTCTTGGCCTGGCCTCC<br>TGCAGTGCCACGCTGCGCGATCGAGTGTTGAATAACTCCATGTGGTAGAGT<br>TATTCAACACTCGATCGCGCAGTGCGGCACATGCTTACCAGCTCTAGGCCA<br>GGGCAGATGGGATATGACGAATGGACTGCCAGCTGGATACAAGGATGCTC<br>ACC**atattatgaagggccttgag

Table S2: Tukey's multiple comparisons test of vector titers (Figure 2B).

Tukey's multiple comparisons test	Mean Diff.	95.00% CI of diff.	Below threshold?	Summary	Adjusted P Value
SS vs. DS	-55000	-339596 to 229596	No	ns	0.9624
SS vs. UV1	-4453333	-4771522 to -4135145	Yes	****	<0.0001
SS vs. UV1-SS	-3241667	-3526263 to -2957070	Yes	****	<0.0001
SS vs. UV1-DS	-3705000	-3989596 to -3420404	Yes	****	<0.0001
DS vs. UV1	-4398333	-4716522 to -4080145	Yes	****	<0.0001
DS vs. UV1-SS	-3186667	-3471263 to -2902070	Yes	****	<0.0001
DS vs. UV1-DS	-3650000	-3934596 to -3365404	Yes	****	<0.0001
UV1 vs. UV1-SS	1211667	893478 to 1529855	Yes	****	<0.0001
UV1 vs. UV1-DS	748333	430145 to 1066522	Yes	***	0.0002
UV1-SS vs. UV1-DS	-463333	-747930 to -178737	Yes	**	0.0027

Table S3: Tukey's multiple comparisons test of anti-sickling globin induction in human SCD CD34+ HSPCs (Figure 3B).

Tukey's multiple comparisons test	Mean Diff.	95.00% CI of diff.	Below threshold?	Summary	Adjusted P Value
mock vs. UV1	-20.47	-38.20 to -2.734	Yes	*	0.0195
mock vs. SS	-17.72	-32.20 to -3.239	Yes	*	0.0126
mock vs. UV1-SS	-41.12	-56.76 to -25.48	Yes	****	<0.0001
mock vs. DS	-24.94	-39.42 to -10.46	Yes	***	0.0006
mock vs. UV1-DS	-51.73	-66.21 to -37.25	Yes	****	<0.0001
UV1 vs. SS	2.750	-14.98 to 20.48	No	ns	0.9952
UV1 vs. UV1-SS	-20.65	-39.34 to -1.957	Yes	*	0.0264
UV1 vs. DS	-4.467	-22.20 to 13.27	No	ns	0.9595
UV1 vs. UV1-DS	-31.26	-49.00 to -13.53	Yes	***	0.0005
SS vs. UV1-SS	-23.40	-39.04 to -7.760	Yes	**	0.0023
SS vs. DS	-7.216	-21.70 to 7.264	No	ns	0.5994
SS vs. UV1-DS	-34.01	-48.49 to -19.53	Yes	****	<0.0001
UV1-SS vs. DS	16.18	0.5434 to 31.82	Yes	*	0.0405
UV1-SS vs. UV1-DS	-10.61	-26.25 to 5.030	No	ns	0.2914
DS vs. UV1-DS	-26.79	-41.27 to -12.31	Yes	***	0.0003

Table S4: Tukey's multiple comparisons test of anti-sickling globin induction normalized to VCN in human SCD CD34+ HSPCs (Figure 3C).

Tukey's multiple comparisons test	Mean Diff.	95.00% CI of diff.	Below threshold?	Summary	Adjusted P Value
UV1 vs. SS	2.820	-5.048 to 10.69	No	ns	0.7819
UV1 vs. UV1-SS	-4.329	-12.62 to 3.966	No	ns	0.4890
UV1 vs. DS	-3.368	-11.24 to 4.501	No	ns	0.6596
UV1 vs. UV1-DS	-9.740	-17.61 to -1.871	Yes	*	0.0136
SS vs. UV1-SS	-7.149	-14.09 to -0.2093	Yes	*	0.0425
SS vs. DS	-6.188	-12.61 to 0.2364	No	ns	0.0610
SS vs. UV1-DS	-12.56	-18.98 to -6.135	Yes	***	0.0003
UV1-SS vs. DS	0.9604	-5.979 to 7.900	No	ns	0.9911
UV1-SS vs. UV1-DS	-5.411	-12.35 to 1.528	No	ns	0.1584
DS vs. UV1-DS	-6.372	-12.80 to 0.05328	No	ns	0.0523

Table S5: Tukey's multiple comparisons test of sickled cells quantification in human SCD CD34+ HSPCs (Figure 3D).

Tukey's multiple comparisons test	Mean Diff.	95.00% CI of diff.	Below threshold?	Summary	Adjusted P Value
mock vs. UV1	26.08	13.57 to 38.58	Yes	****	<0.0001
mock vs. SS	21.08	10.87 to 31.28	Yes	****	<0.0001
mock vs. UV1-SS	38.99	27.97 to 50.02	Yes	****	<0.0001
mock vs. DS	27.00	16.79 to 37.21	Yes	****	<0.0001
mock vs. UV1-DS	44.20	33.99 to 54.41	Yes	****	<0.0001
UV1 vs. SS	-5.000	-17.50 to 7.502	No	ns	0.7810
UV1 vs. UV1-SS	12.92	-0.2612 to 26.09	No	ns	0.0563
UV1 vs. DS	0.9250	-11.58 to 13.43	No	ns	0.9999
UV1 vs. UV1-DS	18.13	5.623 to 30.63	Yes	**	0.0031
SS vs. UV1-SS	17.92	6.891 to 28.94	Yes	**	0.0011
SS vs. DS	5.925	-4.283 to 16.13	No	ns	0.4463
SS vs. UV1-DS	23.13	12.92 to 33.33	Yes	****	<0.0001
UV1-SS vs. DS	-11.99	-23.02 to -0.9662	Yes	*	0.0294
UV1-SS vs. UV1-DS	5.208	-5.817 to 16.23	No	ns	0.6491
DS vs. UV1-DS	17.20	6.992 to 27.41	Yes	***	0.0007

Table S6: Tukey's multiple comparisons test of engraftment of lineage negative bone marrow from BERK mouse (CD45.2) (Figure 4A).

Tukey's multiple comparisons test	Mean Diff.	95.00% CI of diff.	Below threshold?	Summary	Adjusted P Value
SCD vs. UV1	-0.3799	-4.213 to 3.453	No	ns	>0.9999
SCD vs. SS	-1.178	-5.011 to 2.655	No	ns	0.9642
SCD vs. UV1-SS	0.4108	-3.422 to 4.244	No	ns	0.9999
SCD vs. DS	-0.3919	-4.225 to 3.441	No	ns	>0.9999
SCD vs. UV1-DS	-3.110	-6.943 to 0.7231	No	ns	0.1860
SCD vs. Healthy	-2.831	-6.664 to 1.002	No	ns	0.2824
UV1 vs. SS	-0.7985	-4.632 to 3.035	No	ns	0.9952
UV1 vs. UV1-SS	0.7908	-3.042 to 4.624	No	ns	0.9955
UV1 vs. DS	-0.01198	-3.845 to 3.821	No	ns	>0.9999
UV1 vs. UV1-DS	-2.730	-6.563 to 1.103	No	ns	0.3237
UV1 vs. Healthy	-2.451	-6.284 to 1.382	No	ns	0.4539
SS vs. UV1-SS	1.589	-2.244 to 5.422	No	ns	0.8634
SS vs. DS	0.7865	-3.047 to 4.620	No	ns	0.9956
SS vs. UV1-DS	-1.932	-5.765 to 1.902	No	ns	0.7192
SS vs. Healthy	-1.652	-5.485 to 2.181	No	ns	0.8406
UV1-SS vs. DS	-0.8028	-4.636 to 3.030	No	ns	0.9951
UV1-SS vs. UV1-DS	-3.521	-7.354 to 0.3122	No	ns	0.0916
UV1-SS vs. Healthy	-3.242	-7.075 to 0.5915	No	ns	0.1500
DS vs. UV1-DS	-2.718	-6.551 to 1.115	No	ns	0.3288
DS vs. Healthy	-2.439	-6.272 to 1.394	No	ns	0.4599
UV1-DS vs. Healthy	0.2792	-3.554 to 4.112	No	ns	>0.9999

Table S7: Tukey's multiple comparisons test of hemoglobin in BERK SCD mouse model (Figure 4B).

Tukey's multiple comparisons test	Mean Diff.	95.00% CI of diff.	Below threshold?	Summary	Adjusted P Value
SCD vs. UV1	-1.288	-3.280 to 0.7048	No	ns	0.4360
SCD vs. SS	-2.625	-4.617 to -0.6327	Yes	**	0.0032
SCD vs. UV1-SS	-3.100	-5.092 to -1.108	Yes	***	0.0003
SCD vs. DS	-4.625	-6.617 to -2.633	Yes	****	<0.0001
SCD vs. UV1-DS	-4.463	-6.455 to -2.470	Yes	****	<0.0001
SCD vs. Healthy	-6.063	-8.055 to -4.070	Yes	****	<0.0001
UV1 vs. SS	-1.338	-3.330 to 0.6548	No	ns	0.3898
UV1 vs. UV1-SS	-1.813	-3.805 to 0.1798	No	ns	0.0968
UV1 vs. DS	-3.338	-5.330 to -1.345	Yes	****	<0.0001
UV1 vs. UV1-DS	-3.175	-5.167 to -1.183	Yes	***	0.0002
UV1 vs. Healthy	-4.775	-6.767 to -2.783	Yes	****	<0.0001
SS vs. UV1-SS	-0.4750	-2.467 to 1.517	No	ns	0.9898
SS vs. DS	-2.000	-3.992 to -0.007737	Yes	*	0.0485
SS vs. UV1-DS	-1.838	-3.830 to 0.1548	No	ns	0.0886
SS vs. Healthy	-3.438	-5.430 to -1.445	Yes	****	<0.0001
UV1-SS vs. DS	-1.525	-3.517 to 0.4673	No	ns	0.2406
UV1-SS vs. UV1-DS	-1.363	-3.355 to 0.6298	No	ns	0.3675
UV1-SS vs. Healthy	-2.963	-4.955 to -0.9702	Yes	***	0.0006
DS vs. UV1-DS	0.1625	-1.830 to 2.155	No	ns	>0.9999
DS vs. Healthy	-1.438	-3.430 to 0.5548	No	ns	0.3050
UV1-DS vs. Healthy	-1.600	-3.592 to 0.3923	No	ns	0.1934

Table S8: Tukey's multiple comparisons test of hematocrit in BERK SCD mouse model (Figure 4C).

Tukey's multiple comparisons test	Mean Diff.	95.00% CI of diff.	Below threshold?	Summary	Adjusted P Value
SCD vs. UV1	-6.050	-12.75 to 0.6488	No	ns	0.1013
SCD vs. SS	-8.550	-15.25 to -1.851	Yes	**	0.0048
SCD vs. UV1-SS	-10.06	-16.76 to -3.364	Yes	***	0.0005
SCD vs. DS	-14.94	-21.64 to -8.239	Yes	****	<0.0001
SCD vs. UV1-DS	-16.01	-22.71 to -9.314	Yes	****	<0.0001
SCD vs. Healthy	-21.74	-28.44 to -15.04	Yes	****	<0.0001
UV1 vs. SS	-2.500	-9.199 to 4.199	No	ns	0.9099
UV1 vs. UV1-SS	-4.013	-10.71 to 2.686	No	ns	0.5277
UV1 vs. DS	-8.888	-15.59 to -2.189	Yes	**	0.0030
UV1 vs. UV1-DS	-9.963	-16.66 to -3.264	Yes	***	0.0006
UV1 vs. Healthy	-15.69	-22.39 to -8.989	Yes	****	<0.0001
SS vs. UV1-SS	-1.513	-8.211 to 5.186	No	ns	0.9923
SS vs. DS	-6.388	-13.09 to 0.3113	No	ns	0.0708
SS vs. UV1-DS	-7.463	-14.16 to -0.7637	Yes	*	0.0200
SS vs. Healthy	-13.19	-19.89 to -6.489	Yes	****	<0.0001
UV1-SS vs. DS	-4.875	-11.57 to 1.824	No	ns	0.2953
UV1-SS vs. UV1-DS	-5.950	-12.65 to 0.7488	No	ns	0.1122
UV1-SS vs. Healthy	-11.68	-18.37 to -4.976	Yes	****	<0.0001
DS vs. UV1-DS	-1.075	-7.774 to 5.624	No	ns	0.9988
DS vs. Healthy	-6.800	-13.50 to -0.1012	Yes	*	0.0445
UV1-DS vs. Healthy	-5.725	-12.42 to 0.9738	No	ns	0.1402

Table S9: Tukey’s multiple comparisons test of reticulocytes in BERK SCD mouse model (Figure 4D).

Tukey's multiple comparisons test	Mean Diff.	95.00% CI of diff.	Below threshold?	Summary	Adjusted P Value
SCD vs. UV1	25.28	20.29 to 30.26	Yes	****	<0.0001
SCD vs. SS	28.34	23.36 to 33.32	Yes	****	<0.0001
SCD vs. UV1-SS	31.18	26.20 to 36.16	Yes	****	<0.0001
SCD vs. DS	33.73	28.75 to 38.71	Yes	****	<0.0001
SCD vs. UV1-DS	34.89	29.91 to 39.87	Yes	****	<0.0001
SCD vs. Healthy	39.15	34.17 to 44.13	Yes	****	<0.0001
UV1 vs. SS	3.063	-1.920 to 8.045	No	ns	0.4967
UV1 vs. UV1-SS	5.906	0.9240 to 10.89	Yes	*	0.0108
UV1 vs. DS	8.454	3.471 to 13.44	Yes	****	<0.0001
UV1 vs. UV1-DS	9.615	4.633 to 14.60	Yes	****	<0.0001
UV1 vs. Healthy	13.88	8.895 to 18.86	Yes	****	<0.0001
SS vs. UV1-SS	2.844	-2.139 to 7.826	No	ns	0.5839
SS vs. DS	5.391	0.4090 to 10.37	Yes	*	0.0261
SS vs. UV1-DS	6.553	1.570 to 11.53	Yes	**	0.0033
SS vs. Healthy	10.82	5.833 to 15.80	Yes	****	<0.0001
UV1-SS vs. DS	2.548	-2.435 to 7.530	No	ns	0.7004
UV1-SS vs. UV1-DS	3.709	-1.274 to 8.691	No	ns	0.2703
UV1-SS vs. Healthy	7.971	2.989 to 12.95	Yes	***	0.0002
DS vs. UV1-DS	1.161	-3.821 to 6.144	No	ns	0.9909
DS vs. Healthy	5.424	0.4415 to 10.41	Yes	*	0.0247
UV1-DS vs. Healthy	4.263	-0.7198 to 9.245	No	ns	0.1394

Table S10: Tukey's multiple comparisons test of sickled cells in BERK SCD mouse model (Figure 4E).

Tukey's multiple comparisons test	Mean Diff.	95.00% CI of diff.	Below threshold?	Summary	Adjusted P Value
SCD vs. UV1	31.67	25.67 to 37.67	Yes	****	<0.0001
SCD vs. SS	34.13	28.13 to 40.13	Yes	****	<0.0001
SCD vs. UV1-SS	35.67	29.67 to 41.67	Yes	****	<0.0001
SCD vs. DS	38.91	32.91 to 44.91	Yes	****	<0.0001
SCD vs. UV1-DS	39.44	33.44 to 45.44	Yes	****	<0.0001
SCD vs. Healthy	45.14	39.14 to 51.14	Yes	****	<0.0001
UV1 vs. SS	2.459	-3.541 to 8.459	No	ns	0.8664
UV1 vs. UV1-SS	3.996	-2.004 to 9.995	No	ns	0.3995
UV1 vs. DS	7.242	1.242 to 13.24	Yes	**	0.0089
UV1 vs. UV1-DS	7.771	1.771 to 13.77	Yes	**	0.0040
UV1 vs. Healthy	13.47	7.466 to 19.47	Yes	****	<0.0001
SS vs. UV1-SS	1.536	-4.464 to 7.536	No	ns	0.9852
SS vs. DS	4.782	-1.218 to 10.78	No	ns	0.2004
SS vs. UV1-DS	5.312	-0.6879 to 11.31	No	ns	0.1144
SS vs. Healthy	11.01	5.007 to 17.01	Yes	****	<0.0001
UV1-SS vs. DS	3.246	-2.754 to 9.246	No	ns	0.6430
UV1-SS vs. UV1-DS	3.776	-2.224 to 9.776	No	ns	0.4683
UV1-SS vs. Healthy	9.471	3.471 to 15.47	Yes	***	0.0002
DS vs. UV1-DS	0.5296	-5.470 to 6.530	No	ns	>0.9999
DS vs. Healthy	6.225	0.2249 to 12.22	Yes	*	0.0373
UV1-DS vs. Healthy	5.695	-0.3048 to 11.69	No	ns	0.0731

Table S11: Tukey's multiple comparisons test of erythroid precursor cells in BERK SCD mouse model (Figure 4F).

Tukey's multiple comparisons test	Mean Diff.	95.00% CI of diff.	Below threshold?	Summary	Adjusted P Value
SCD vs. UV1	28.70	23.00 to 34.40	Yes	****	<0.0001
SCD vs. SS	31.85	26.15 to 37.55	Yes	****	<0.0001
SCD vs. UV1-SS	32.87	27.17 to 38.57	Yes	****	<0.0001
SCD vs. DS	35.97	30.27 to 41.67	Yes	****	<0.0001
SCD vs. UV1-DS	36.67	30.97 to 42.37	Yes	****	<0.0001
SCD vs. Healthy	40.50	34.80 to 46.20	Yes	****	<0.0001
UV1 vs. SS	3.154	-2.546 to 8.854	No	ns	0.6188
UV1 vs. UV1-SS	4.171	-1.529 to 9.871	No	ns	0.2891
UV1 vs. DS	7.273	1.572 to 12.97	Yes	**	0.0048
UV1 vs. UV1-DS	7.969	2.269 to 13.67	Yes	**	0.0015
UV1 vs. Healthy	11.80	6.101 to 17.50	Yes	****	<0.0001
SS vs. UV1-SS	1.018	-4.683 to 6.718	No	ns	0.9979
SS vs. DS	4.119	-1.581 to 9.819	No	ns	0.3034
SS vs. UV1-DS	4.815	-0.8851 to 10.52	No	ns	0.1494
SS vs. Healthy	8.648	2.947 to 14.35	Yes	***	0.0005
UV1-SS vs. DS	3.101	-2.599 to 8.801	No	ns	0.6370
UV1-SS vs. UV1-DS	3.798	-1.903 to 9.498	No	ns	0.3990
UV1-SS vs. Healthy	7.630	1.930 to 13.33	Yes	**	0.0027
DS vs. UV1-DS	0.6962	-5.004 to 6.396	No	ns	0.9998
DS vs. Healthy	4.529	-1.171 to 10.23	No	ns	0.2035
UV1-DS vs. Healthy	3.833	-1.868 to 9.533	No	ns	0.3880

Table S12: Tukey's multiple comparisons test of VCN in BERK SCD mouse model (Figure 5A).

Tukey's multiple comparisons test	Mean Diff.	95.00% CI of diff.	Below threshold?	Summary	Adjusted P Value
UV1 vs. SS	3.099	-1.859 to 8.056	No	ns	0.3914
UV1 vs. UV1-SS	-7.673	-12.63 to -2.715	Yes	***	0.0008
UV1 vs. DS	-3.921	-8.879 to 1.036	No	ns	0.1775
UV1 vs. UV1-DS	-14.60	-19.55 to -9.637	Yes	****	<0.0001
SS vs. UV1-SS	-10.77	-15.73 to -5.814	Yes	****	<0.0001
SS vs. DS	-7.020	-11.98 to -2.062	Yes	**	0.0022
SS vs. UV1-DS	-17.69	-22.65 to -12.74	Yes	****	<0.0001
UV1-SS vs. DS	3.751	-1.206 to 8.709	No	ns	0.2127
UV1-SS vs. UV1-DS	-6.923	-11.88 to -1.965	Yes	**	0.0026
DS vs. UV1-DS	-10.67	-15.63 to -5.716	Yes	****	<0.0001

Table S13: Tukey's multiple comparisons test of anti-sickling globin induction normalized to VCN in BERK SCD mouse model (Figure 5C).

Tukey's multiple comparisons test	Mean Diff.	95.00% CI of diff.	Below threshold?	Summary	Adjusted P Value
UV1 vs. SS	3.099	-1.859 to 8.056	No	ns	0.3914
UV1 vs. UV1-SS	-7.673	-12.63 to -2.715	Yes	***	0.0008
UV1 vs. DS	-3.921	-8.879 to 1.036	No	ns	0.1775
UV1 vs. UV1-DS	-14.60	-19.55 to -9.637	Yes	****	<0.0001
SS vs. UV1-SS	-10.77	-15.73 to -5.814	Yes	****	<0.0001
SS vs. DS	-7.020	-11.98 to -2.062	Yes	**	0.0022
SS vs. UV1-DS	-17.69	-22.65 to -12.74	Yes	****	<0.0001
UV1-SS vs. DS	3.751	-1.206 to 8.709	No	ns	0.2127
UV1-SS vs. UV1-DS	-6.923	-11.88 to -1.965	Yes	**	0.0026
DS vs. UV1-DS	-10.67	-15.63 to -5.716	Yes	****	<0.0001

Table S14: Tukey's multiple comparisons test of spleen mass in BERK SCD mouse model (Figure 5C).

Tukey's multiple comparisons test	Mean Diff.	95.00% CI of diff.	Below threshold?	Summary	Adjusted P Value
SCD vs. UV1	0.2325	0.1495 to 0.3155	Yes	****	<0.0001
SCD vs. SS	0.2938	0.2108 to 0.3767	Yes	****	<0.0001
SCD vs. UV1-SS	0.3275	0.2445 to 0.4105	Yes	****	<0.0001
SCD vs. DS	0.3663	0.2833 to 0.4492	Yes	****	<0.0001
SCD vs. UV1-DS	0.3850	0.3020 to 0.4680	Yes	****	<0.0001
SCD vs. Healthy	0.4625	0.3795 to 0.5455	Yes	****	<0.0001
UV1 vs. SS	0.06125	-0.02172 to 0.1442	No	ns	0.2794
UV1 vs. UV1-SS	0.09500	0.01203 to 0.1780	Yes	*	0.0154
UV1 vs. DS	0.1338	0.05078 to 0.2167	Yes	***	0.0002
UV1 vs. UV1-DS	0.1525	0.06953 to 0.2355	Yes	****	<0.0001
UV1 vs. Healthy	0.2300	0.1470 to 0.3130	Yes	****	<0.0001
SS vs. UV1-SS	0.03375	-0.04922 to 0.1167	No	ns	0.8705
SS vs. DS	0.07250	-0.01047 to 0.1555	No	ns	0.1236
SS vs. UV1-DS	0.09125	0.008283 to 0.1742	Yes	*	0.0225
SS vs. Healthy	0.1688	0.08578 to 0.2517	Yes	****	<0.0001
UV1-SS vs. DS	0.03875	-0.04422 to 0.1217	No	ns	0.7799
UV1-SS vs. UV1-DS	0.05750	-0.02547 to 0.1405	No	ns	0.3518
UV1-SS vs. Healthy	0.1350	0.05203 to 0.2180	Yes	***	0.0001
DS vs. UV1-DS	0.01875	-0.06422 to 0.1017	No	ns	0.9923
DS vs. Healthy	0.09625	0.01328 to 0.1792	Yes	*	0.0135
UV1-DS vs. Healthy	0.07750	-0.005467 to 0.1605	No	ns	0.0815

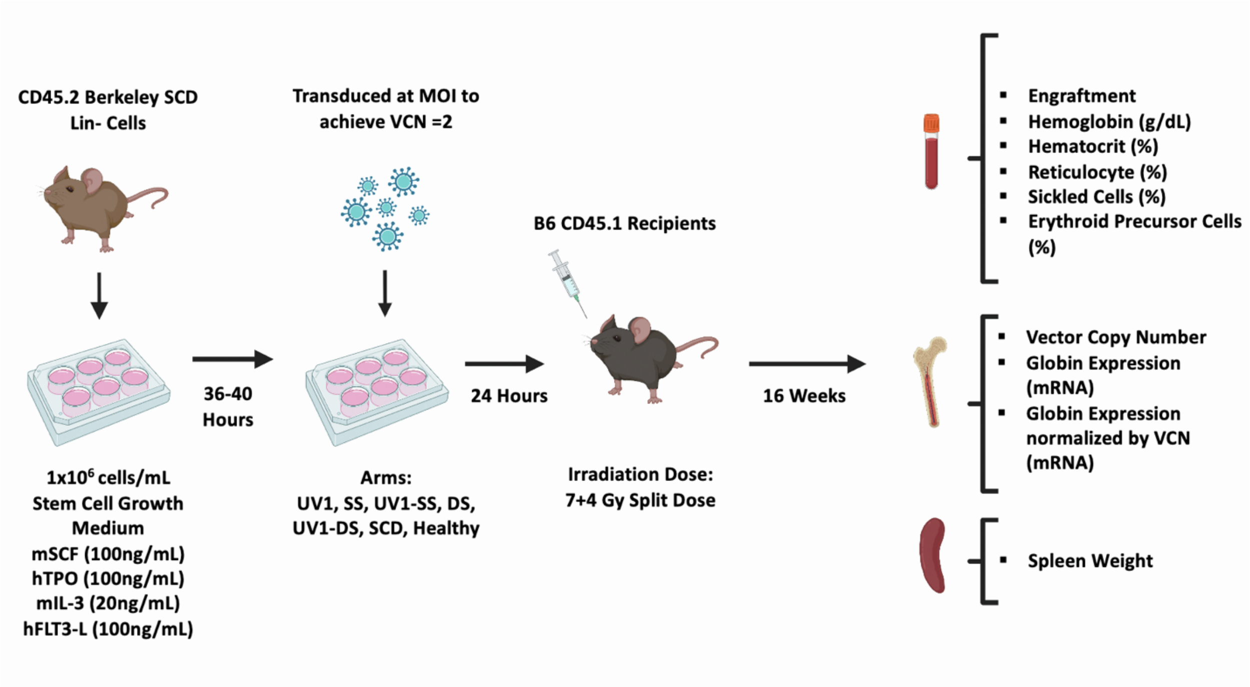

Figure S1. Schematic of experimental plan for the *in vivo* Berkeley SCD mouse model. Image created with BioRender.

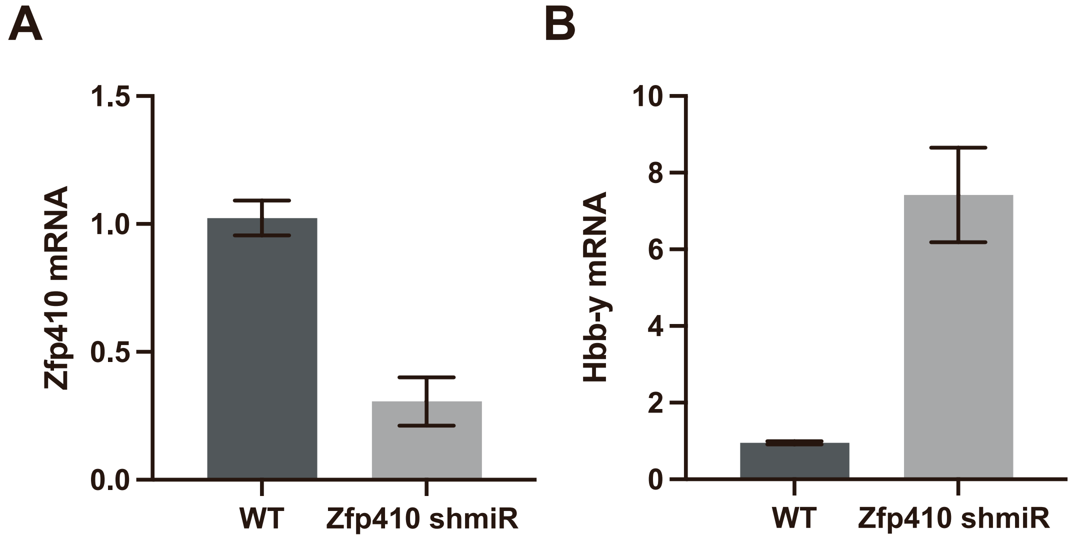

Figure S2. Efficient knockdown of Zfp410 by Zfp410 shmiR vector leads to high Hbb-y induction in erythroid differentiated MEL cells *in vitro*. Zfp410 and Hbb-y mRNA expression as measured by RT–qPCR with Gapdh as control. Data represent mean ± SD.

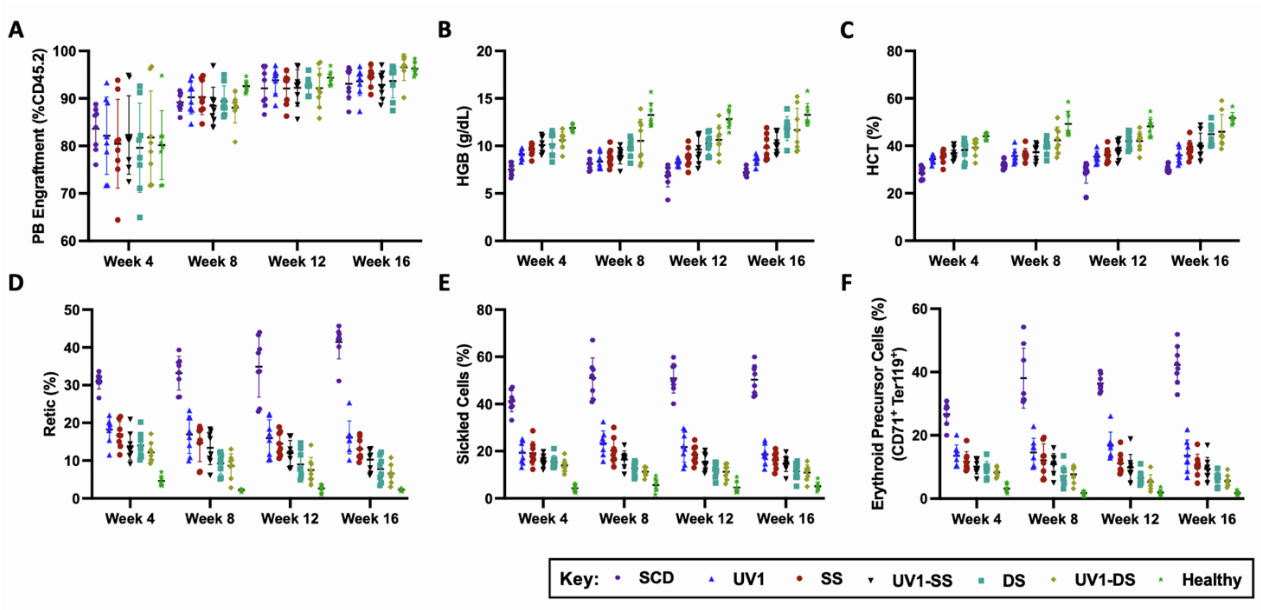

Figure S3. Correction of peripheral blood sickle cell hematologic parameters *in vivo* in Berkeley SCD mouse model (Weeks 4, 8, 12, 16). Lineage-negative (lin-) bone marrow cells from BERK mice (CD45.2) were transduced with each vector or mock-transduced as control and transplanted into irradiated CD45.1+ BL/6 mouse recipients. Mice were bled at 4, 8, 12, and 16 weeks after transplant and peripheral blood (PB) was analyzed. (A) Engraftment was assessed in PB by flow cytometry (%CD45.2+ cells). (B) Hemoglobin (g/dL) , (C) hematocrit and (D) reticulocyte counts (%) are shown. (E) PB was treated with sodium metabisulfite for 30 min to induce sickling. Percentage of sickled RBCs from PB sample was quantified. (F) Percentages of CD71+ Ter119+ high erythroid precursor cell population in PB. Error bars represent mean ± SD. Symbols indicate mice transplanted with different shmiR vectors or non-transduced cells (SCD); each data point represents an individual mouse, N=8

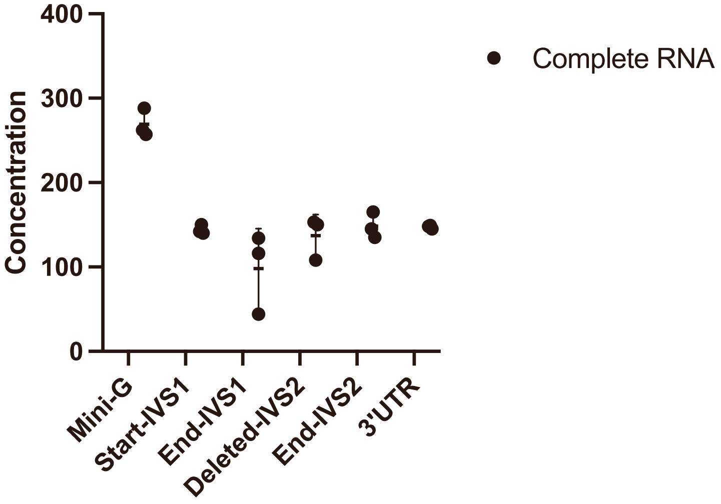

Figure S4: Assessment of complete viral RNA from unconcentrated viral supernatant. Vectors were packaged with an HEK293T *PKR* knock-out cell line. Unconcentrated viral supernatants were collected, and viral RNA was extracted and quantified with ddPCR. Each point on the plot represents a vector packaged from an individual 10cm plate. Error bars represent mean ± SD.

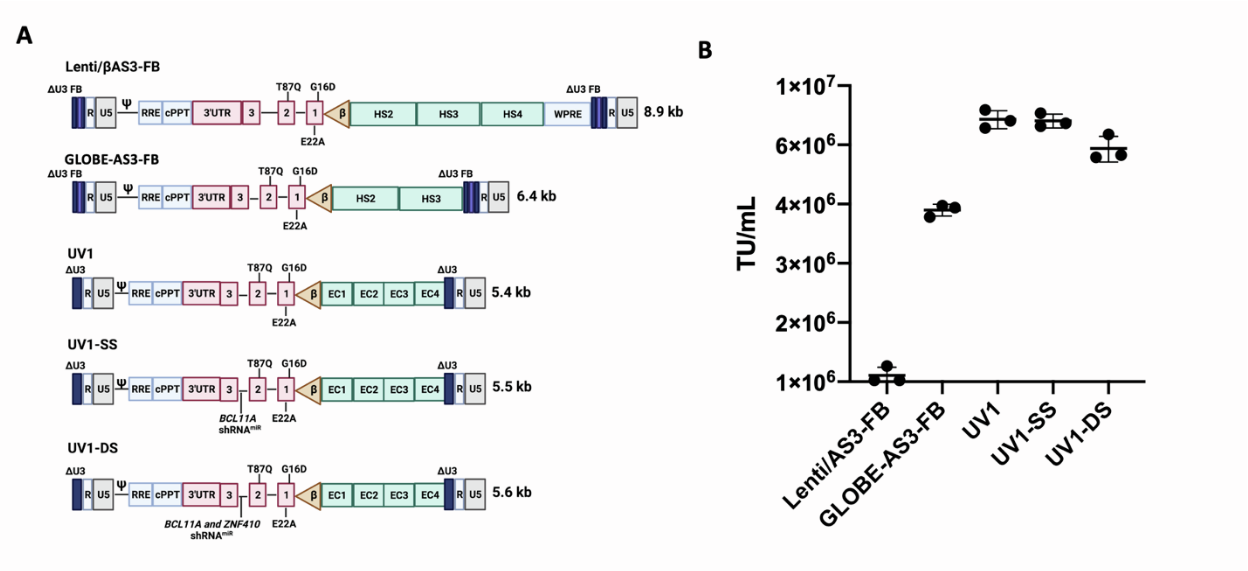

Figure S5: UV1-shmiR vectors maintain high titers compared to other β^{AS3}-globin LVs. (A) Schematics of β^{AS3}-globin LVs: Lenti/β^{AS3}-FB[2] (8.9kb), GLOBE-AS3-FB[4] (6.4kb), UV1[5] (5.4kb), UV1-SS (5.5kb), UV1-DS (5.6kb). Vectors were packaged with an HEK293T *PKR* knock-out cell line and titers were determined by HT-29 cell line transduction, using unconcentrated viral supernatant, and quantified with ddPCR. Each point on the plot represents vector packaged and titered from an individual 10cm plate. Error bars represent mean ± SD. Image created with BioRender.

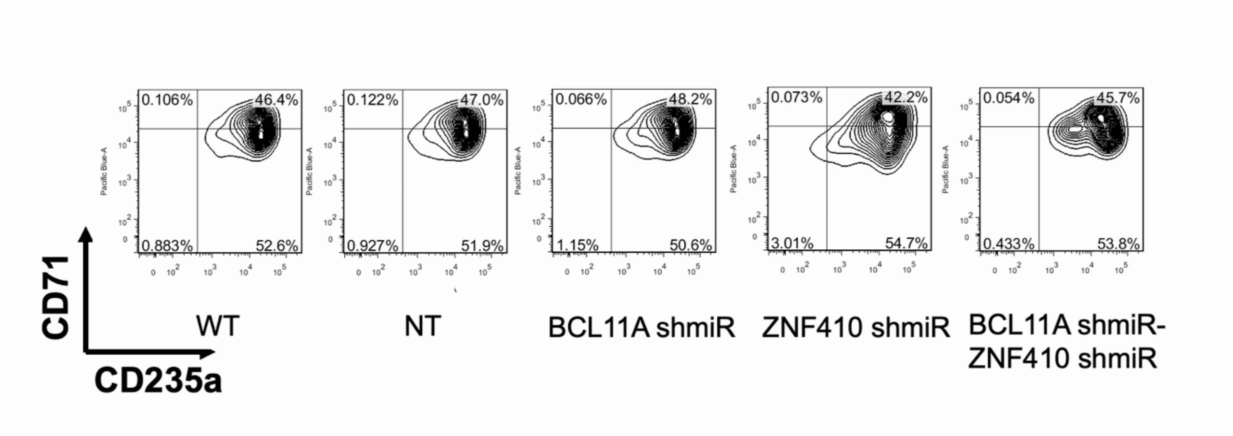

Figure S6. Differentiation status of erythroid cells after 18 days in culture using CD71 and CD235a, WT is wild type cells, NT is CD34+ cells transduced with lentivirus contain non-target shmiR, BCL11A shmiR is CD34+ cells transduced with lentivirus contain BCL11A shmiR, ZNF410 shmiR is CD34+ cells transduced with lentivirus contain ZNF410 shmiR, BCL11A shmiR-ZNF410 shmiR is CD34+ cells transduced with lentivirus contain BCL11A shmiR and ZNF410 shmiR.

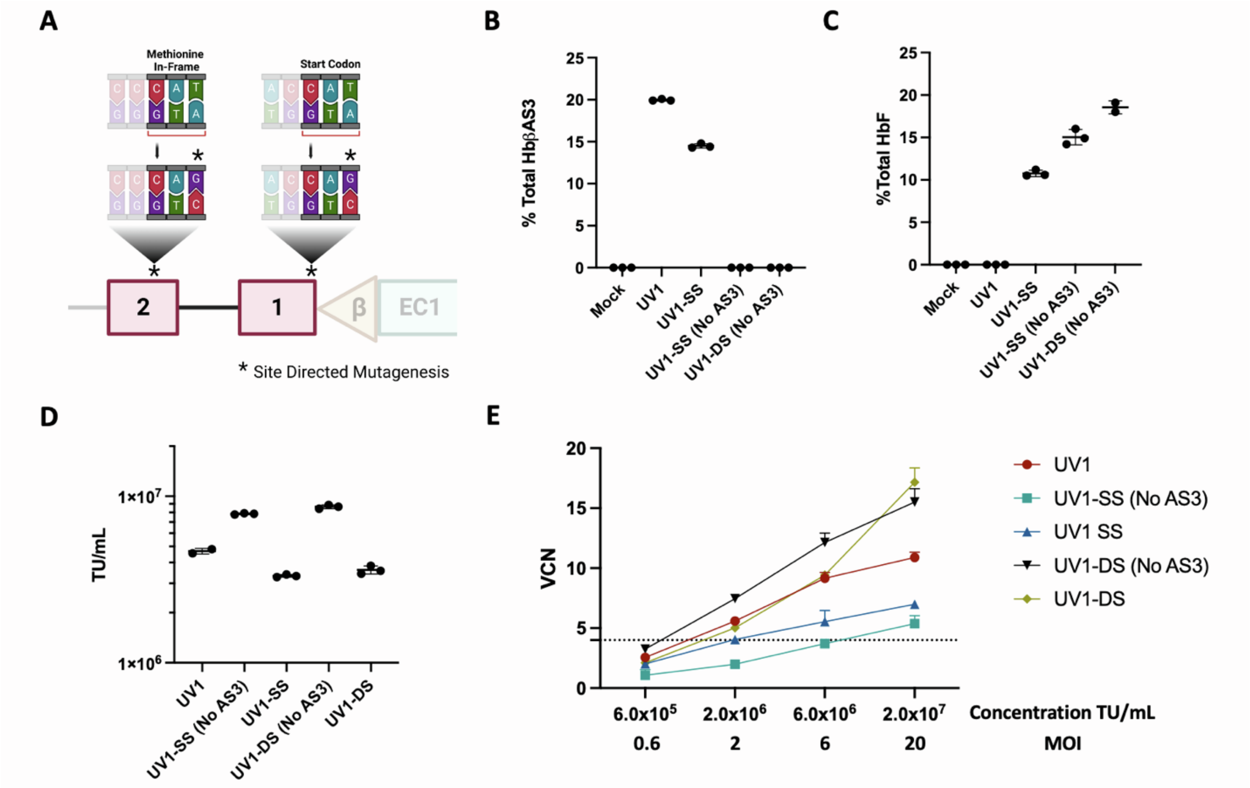

Figure S7. Fetal globin positive control vector design, titer, and gene transfer. (A) Site-directed mutagenesis strategy to change methionine codons (ATG) in the β^{AS3}-globin open reading frame (B) HUDEP-2 parental cells were transduced with vectors at 3.0×10^5 TU/mL and then subjected to erythroid differentiation *in vitro* for 12 days and protein was assessed by HPLC. Induction of HbB^{AS3} (C) Induction of HbF (D) Vectors were packaged with an HEK293T *PKR* knock-out cell line and titers were determined by HT-29 cell line transduction, using raw viral supernatant, and quantified with ddPCR. Each point on the plot represents vector packaged and titered from an individual 10cm plate. (E) Lin- cells from Townes mice were transduced with constructs at 6×10^5 TU/mL, 2×10^6 TU/mL, 6×10^6 TU/mL and 2×10^7 TU/mL (MOI: 0.6, 2, 6, and 20) and cultured for 14 days under myeloid differentiation conditions to assess levels of infectivity. Vector copy number (VCN) was measured by ddPCR. Error bars represent mean ± SD. Image created with BioRender.

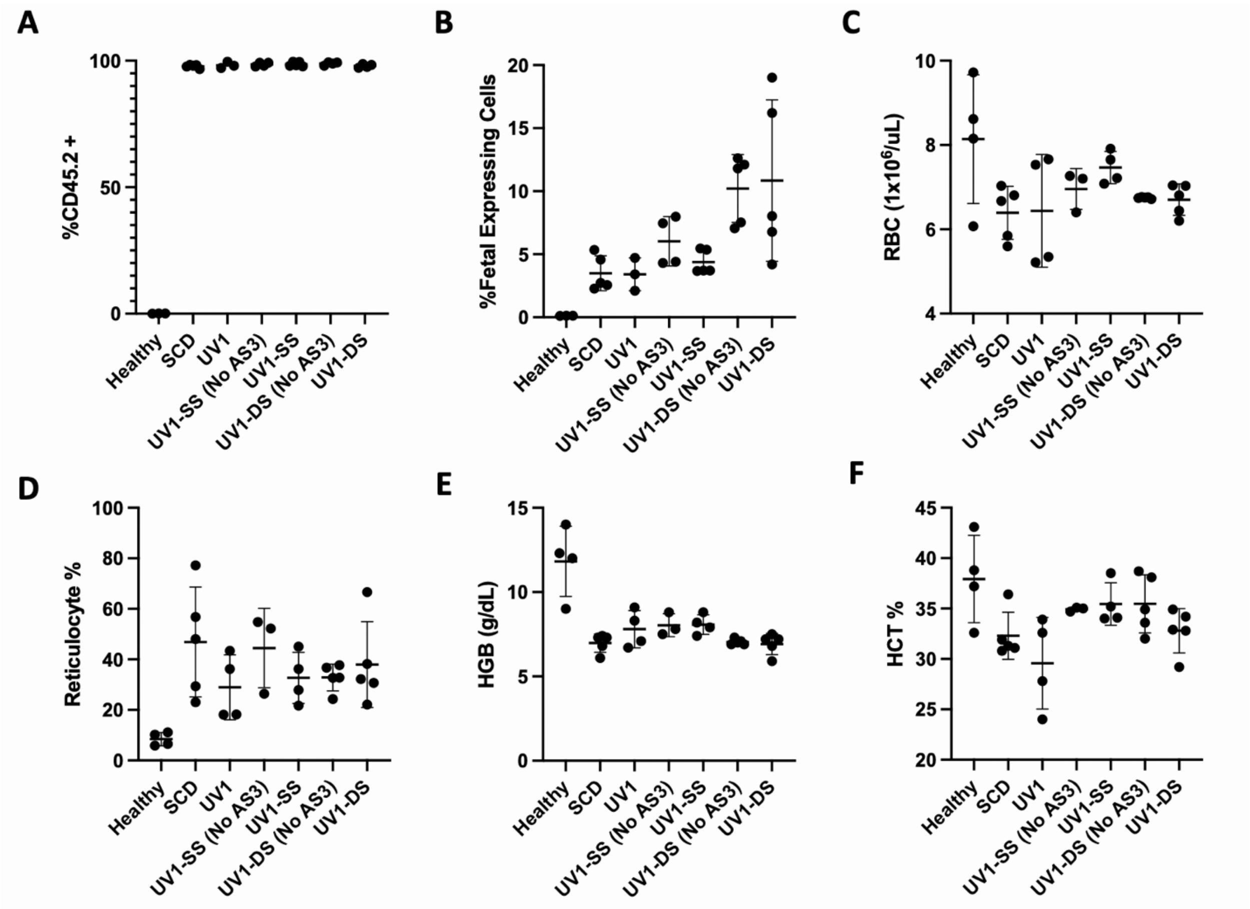

Figure S8. Peripheral blood sickle cell disease erythroid cell parameters *in vivo* in Townes SCD mouse model. Lineage negative (lin-) bone marrow cells from Townes mice (CD45.2) were transduced with each vector or mock-transduced as control and transplanted into irradiated B6 CD45.1+ (Pep Boy) mouse recipients. Mice were bled at 16 weeks after transplant and PB was analyzed. (A) Engraftment was assessed in PB by flow cytometry (%CD45.2+ cells). (B) Percentage of fetal globin expressing cells was assessed by intracellular staining and flow cytometry. (C) Red blood cell counts ($1x10^6$/uL) (D) Reticulocyte counts (%). (E) Hemoglobin (g/dL). (F) Hematocrit. Error bars represent mean ± SD. Each data point represents an individual mouse.

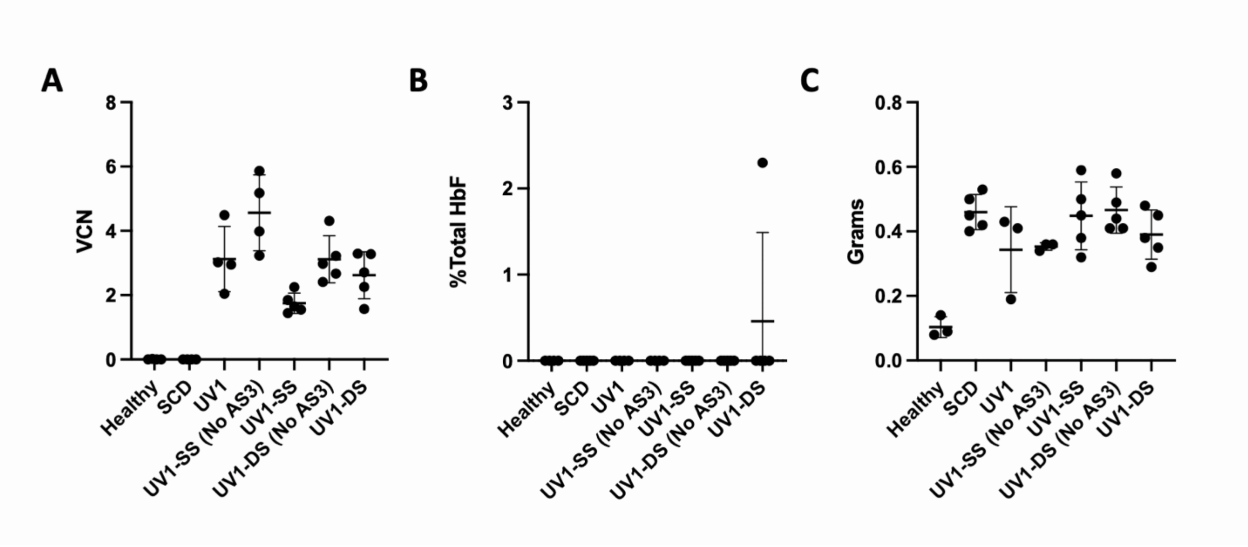

Figure S9. Vector Copy Number, HbF Expression by HPLC, and Spleen Weight

Mice were euthanized at 16 weeks after transplant and whole bone marrow (BM) and spleen was harvested and analyzed individually. (A) VCN in BM was determined by ddPCR. (B) Percentages of HbF expression was determined by HPLC. (C) Spleen weights. Error bars represent mean ± SD. Each data point represents an individual mouse.

References

1. Ryan, T.M., Ciavatta, D.J., and Townes, T.M. (1997). Knockout-Transgenic Mouse Model of Sickle Cell Disease. Science *278*, 873–876. 10.1126/science.278.5339.873.

2. Levasseur, D.N., Ryan, T.M., Pawlik, K.M., and Townes, T.M. (2003). Correction of a mouse model of sickle cell disease: lentiviral/antisickling beta-globin gene transduction of unmobilized, purified hematopoietic stem cells. Blood *102*, 4312–4319. 10.1182/blood-2003-04-1251.

3. Woodard, K.J., Doerfler, P.A., Mayberry, K.D., Sharma, A., Levine, R., Yen, J., Valentine, V., Palmer, L.E., Valentine, M., and Weiss, M.J. (2022). Limitations of mouse models for sickle cell disease conferred by their human globin transgene configurations. Disease Models & Mechanisms *15*, dmm049463. 10.1242/dmm.049463.

4. Poletti, V., Urbinati, F., Charrier, S., Corre, G., Hollis, R.P., Campo Fernandez, B., Martin, S., Rothe, M., Schambach, A., Kohn, D.B., et al. (2018). Pre-clinical Development of a Lentiviral Vector Expressing the Anti-sickling βAS3 Globin for Gene Therapy for Sickle Cell Disease. Mol Ther Methods Clin Dev *11*, 167–179. 10.1016/j.omtm.2018.10.014.

5. Morgan, R.A., Unti, M.J., Aleshe, B., Brown, D., Osborne, K.S., Koziol, C., Ayoub, P.G., Smith, O.B., O'Brien, R., Tam, C., et al. (2020). Improved Titer and Gene Transfer by Lentiviral Vectors Using Novel, Small β-Globin Locus Control Region Elements. Molecular Therapy *28*, 328–340. 10.1016/j.ymthe.2019.09.020.
